# Silylium-Ion-Promoted Formation of Methylenecyclobutenes by Formal (2 + 2) Cycloaddition of Allenylsilanes and Internal Alkynes

**DOI:** 10.1021/acs.joc.5c01473

**Published:** 2025-07-15

**Authors:** Lei Deng, Honghua Zuo, Hendrik F. T. Klare, Martin Oestreich

**Affiliations:** Institut für Chemie, Technische Universität Berlin, Straße des 17. Juni 115, 10623 Berlin, Germany

## Abstract

A (2 + 2) cycloaddition of propargylsilanes and internal alkynes to afford densely substituted methylenecyclobutenes is reported. The propargylsilane is generated in situ by rapid silylium-ion-catalyzed isomerization of the corresponding allenylsilane through the intermediacy of a β,β*′*-bis­(silicon)-stabilized vinyl cation. The stepwise cycloaddition is initiated by a silylium ion, and the (2 + 2) pathway hinges on the steric demand of the α-substituent of the allenylsilane (R = 2° alkyl). Smaller groups (R = 1° alkyl) lead to the known formation of the (3 + 2) annulation product.

## Graphical abstract

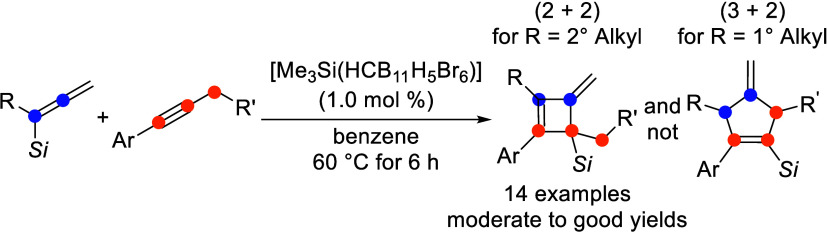

Vinyl cations, once considered elusive, emerged as versatile intermediates in synthesis over the past decades.^1–6^ Our laboratory recently showed that β-silicon-stabilized vinyl cations,^7–9^ catalytically generated from internal alkynes **1** and counteranion-stabilized silylium ions,^10^ offer a new entry into synthetically useful transformations.^11–14^ An intriguing example is the silylium-ion-promoted (3 + 2) cycloaddition of aryl-substituted alkynes **1** with allenylsilanes **2** to give 4-methylenecyclopentenes **8** (Scheme 1, right).^13^ In this reaction, the allenylsilane **2** is rapidly isomerized into the corresponding propargylsilane **3** through the intermediacy of β,β*′*-bis­(silicon)-stabilized vinyl cation **4**, the actual silylium-ion carrier. The aforementioned vinyl cation **5** forms by silylium-ion transfer from **4** to the C–C triple bond of **1**, followed by its addition across the propargylsilane **3**. The resulting vinyl cation **6** with a conjugated double bond is the key intermediate, subsequently engaging in a [1,5]-hydride shift (**6** → **7**) and a conrotatory 4π-electrocyclization of **7** (not shown). We found that this pathway is only possible when the R group initially introduced with the allenylsilane **2** is small, i.e., a 1° alkyl or an aryl substituent. However, when R is a 2° alkyl residue the (3 + 2) annulation is redirected into a (2 + 2) cycloaddition, affording methylenecyclobutenes **10** rather than the five-membered ring products **8** (Scheme 1, left). We believe that a larger R group increases the dihedral angle between the two alkene units in **6** in such a way (gray box) that the [1,5]-hydride shift is energetically disfavored. Instead, intermediate **6** undergoes a cationic cyclization to give *nonclassical* cyclobutenyl cation **9** ^15^ (depicted as a classical carbenium ion) and eventually methylenecyclobutenes **10** after loss of the silylium-ion promoter.

**Scheme 1:**
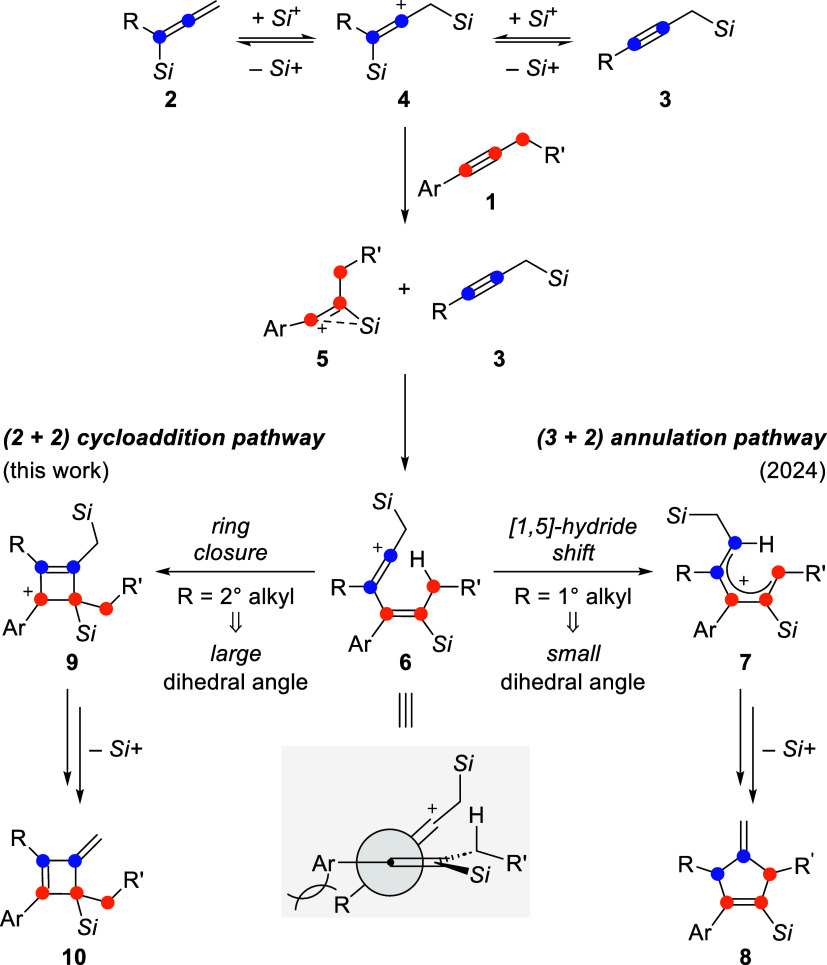
Dichotomy of the Silylium-Ion-Promoted Cycloaddition of Allenylsilanes and Internal Alkynes^a^

a Ar = aryl; any of the R groups = aryl and/or alkyl; *S_i_* = triorganosilyl. The counteranion is omitted for clarity.

Related (2 + 2) cycloaddition reactions involving vinyl cations emerging from protonation of alkynes with strong Brønsted acids are known, and the seminal work of Griesbaum^16^ and Olah^17^ is particularly noteworthy. Additional contributions include Hanack’s studies on vinyl cations generated by leaving-group abstraction and their (2 + 2) cycloadditions,^18^ as well as Mayr’s demonstration of methylenecyclobutene formation from allenyl cation-alkene cycloadditions.^19^ Pertinent to the present study and different from the above literature precedent, Krossing described a cyclizative dimerization of but-2-yne by the action of a silylium-ion-like reagent.^15^ However, the silylium-ion-promoted method disclosed here provides a novel access to densely substituted cyclobutene rings with an *exo*-methylene group. In contrast to the above-mentioned stoichiometric reactions, catalytic amounts of the silylium-ion initiator are sufficient, and the catalytic cycle is subsequently maintained by the self-regeneration of the silylium-ion promoter.^13^ Furthermore, transition-metal-catalyzed (2 + 2) cycloadditions have emerged as a powerful strategy for constructing such motifs, enabling efficient and regioselective synthesis of methylenecyclobutenes from allenes and alkynes^20,21^ or from two alkynes^22^ under mild conditions with broad substrate scope.

Based on the consideration outlined in Scheme 1, we selected α-cyclopentyl-substituted allenylsilane **2a** and 1-phenylprop-1-yne (**1a**) as model substrates (Table 1). The desired (2 + 2) cycloaddition product **10aa** was obtained in 60% yield after 12 h when using 1.0 mol % of [Me_3_Si­(HCB_11_H_5_Br_6_)] as the initiator in benzene at 60 °C (entry 1). A screening of different arene solvents showed that benzene is superior to all of them (entries 2–6). Other counteranion-stabilized silylium ions such as [Et_3_Si­(HCB_11_H_5_Br_6_)] and [*i*Pr_3_Si­(HCB_11_H_5_Br_6_)] brought about somewhat lower yields of 43% and 55%, respectively (not shown). Toluene-stabilized [Et_3_Si­(toluene)]­[B­(C_6_F_5_)_4_] led to a substantially diminished yield (entry 7; cf. entry 2 with toluene as a solvent). As for the temperature, both a lower and a higher temperature than 60 °C led to reduced yields (entries 8 and 9). To further boost the reaction efficiency, the reaction time (entries 10 and 11) and amount of the internal alkyne **1a** were probed (entries 12–14). The highest yield of **10aa** was obtained using 1.10 equiv of **1a** after 6 h at 60 °C (entry 12).

**Table 1:**
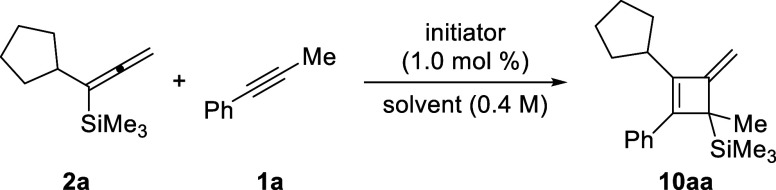
Optimization of Reaction Conditions^a^

entry	initiator	solvent	equiv of **1a**	time (h)	temp (°C)	yield (%)^b^
1	[Me_3_Si(HCB_11_H_5_Br_6_)]	PhH	1.25	12	60	60
2	[Me_3_Si(HCB_11_H_5_Br_6_)]	toluene	1.25	12	60	31
3	[Me_3_Si(HCB_11_H_5_Br_6_)]	PhF	1.25	12	60	47
4	[Me_3_Si(HCB_11_H_5_Br_6_)]	PhCl	1.25	12	60	51
5	[Me_3_Si(HCB_11_H_5_Br_6_)]	PhCF_3_	1.25	12	60	7
6	[Me_3_Si(HCB_11_H_5_Br_6_)]	*p*-xylene	1.25	12	60	25
7	[Et_3_Si(toluene)][B(C_6_F_5_)_4_]	PhH	1.25	12	60	15
8	[Me_3_Si(HCB_11_H_5_Br_6_)]	PhH	1.25	12	RT	31
9	[Me_3_Si(HCB_11_H_5_Br_6_)]	PhH	1.25	12	80	46
10	[Me_3_Si(HCB_11_H_5_Br_6_)]	PhH	1.25	4	60	30
11	[Me_3_Si(HCB_11_H_5_Br_6_)]	PhH	1.25	6	60	60
12	[Me_3_Si(HCB_11_H_5_Br_6_)]	PhH	1.10	6	60	62 (55)^c^
13	[Me_3_Si(HCB_11_H_5_Br_6_)]	PhH	1.25	6	60	60
14	[Me_3_Si(HCB_11_H_5_Br_6_)]	PhH	1.50	6	60	33

a All reactions were performed on a 0.20 mmol scale under an argon atmosphere in 0.5 mL of the indicated solvent.

b Yields were determined by ^1^H NMR spectroscopy using CH_2_Br_2_ as an internal standard.

c Isolated yield after flash chromatography on silica gel in parentheses.

Having established the optimal conditions, we examined the generality of this (2 + 2) cycloaddition reaction (Scheme 2). A 53% isolated yield was obtained for the model reaction on a 1.0 mmol scale. Variation of the alkyne component **1** by changing the *para*-substituent of the aryl group revealed a strong electronic effect. While halogen atoms enhanced the yield (**10ba**–**da**), yields were low with methyl (**10ea**) and phenyl (**10fa**). An electron-withdrawing group such as trifluoromethyl did not lead to any product formation of **10ga**; the allenylsilane **2a** underwent partial isomerization to the propargylsilane **3a** (not shown). The same trend was seen with *ortho*- and *meta*-substituted derivatives **10ha**–**ja** and **10ka**–**ma**, respectively. A thienyl instead of a phenyl group did not afford any product **10na**, only the starting materials were detected by ^1^H NMR spectroscopy. We speculate that the thienyl group cannot sufficiently stabilize the cyclobutenyl cation intermediate **9** (cf. Scheme 1, left); this could also explain the reaction outcome with the (trifluoromethyl)­phenyl group (*vide supra*). Any deviation from the methyl group in **1** (R′ = H) completely thwarted the cycloaddition as shown for **1o** and **1p**. We then examined a series of allenylsilanes **2b**–**e** bearing different branched alkyl groups in the α-position. Cycloalkyl groups other than cyclopentyl furnished the cycloadducts **10ab** (R = cyclohexyl) and **10ac** (R = cycloheptyl) in good and moderate yield, respectively. An acyclic secondary alkyl group such as isopropyl was tolerated to give **10ad**; in this case, the formation of the (3 + 2) annulated product from the competing pathway was seen in minor quantities (not shown). However, a tertiary group was too bulky, and **10ae** did not form; again only isomerization of the allenylsilane was observed.

**Scheme 2:**
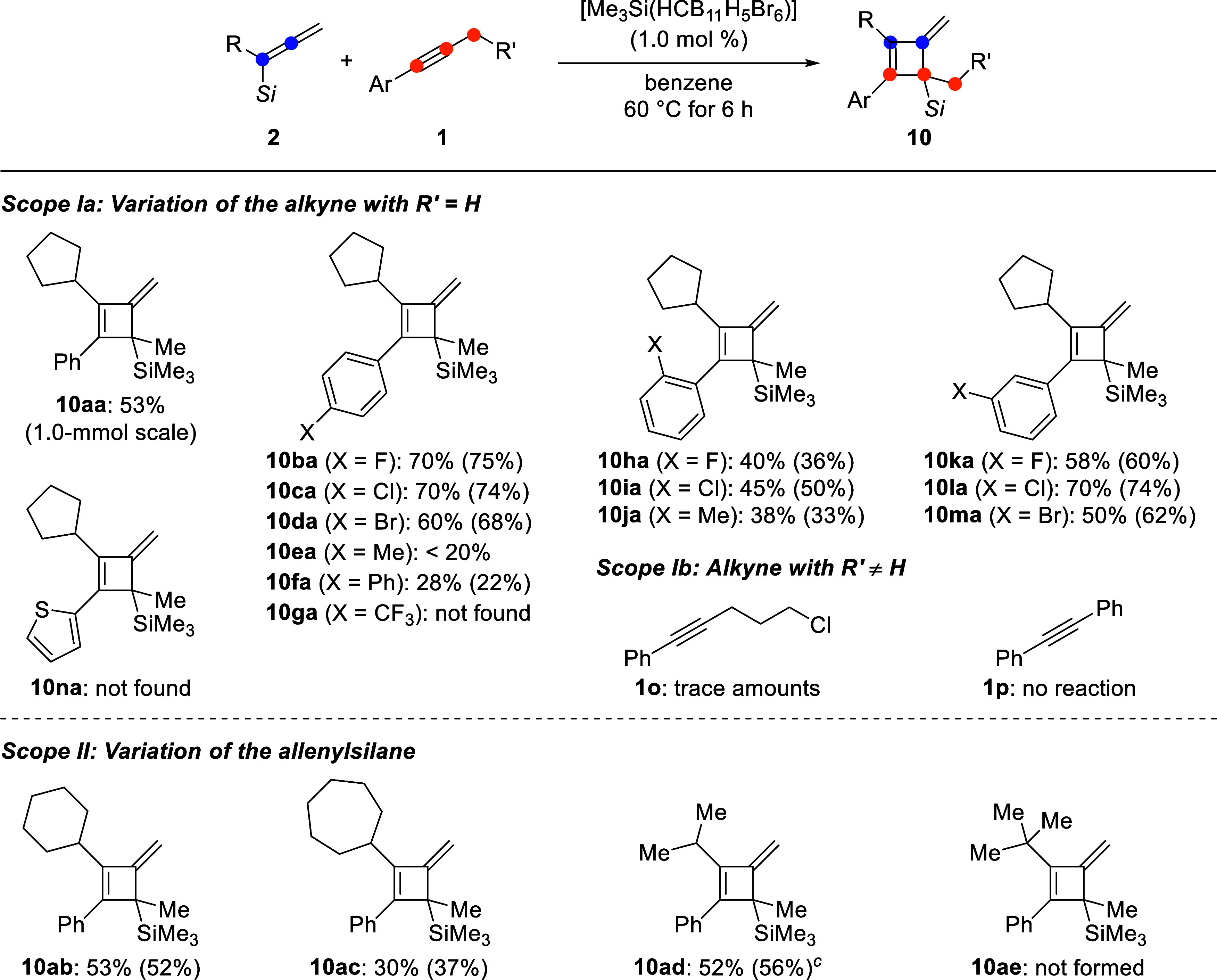
Substrate Scope of the (2 + 2) Cycloaddition^b^ ^c^

a All reactions were performed with the indicated allenylsilane **2** (0.20 mmol) and alkyne **1** (0.22 mmol, 1.10 equiv) as well as the initiator [Me_3_Si­(HCB_11_H_5_Br_6_)] (2.0 μmol, 1.0 mol %) under argon atmosphere in benzene (0.5 mL) at 60 °C for 6 h.
b Isolated yields refer to analytically pure material after flash chromatography on silica gel; yields in parentheses were determined by ^1^H NMR spectroscopy using CH_2_Br_2_ as an internal standard.
c The (3 + 2) cycloaddition product was formed in less than 15% yield, as estimated by ^1^H NMR spectroscopic analysis.

To summarize, we added an alternative reaction pathway to the silylium-ion-promoted (formal) cycloaddition chemistry of α-substituted allenylsilanes and internal alkynes. The steric bulk of the substitutent in the α-position of the allenylsilane determines the reaction outcome: small (1°) alkyl groups lead to the known (3 + 2) annulation^13^ and branched (2°) alkyl groups to the presented (2 + 2) cycloaddition. Highly substituted methylenecyclobutenes become accessible in the latter case. The common theme of these reactions^13,14^ is the intermediacy of a β-silicon-stabilized vinyl cation, highlighting again the great synthetic potential of silylium-ion catalysis.

## Supplementary Material



## Data Availability

The data underlying this study are available in the published article and its Supporting Information.

## Notes

General procedures, experimental details, and characterization and spectral data for all new compounds (PDF)
