# Supplementary material for: Silylium-Ion-Promoted Formation of Methylenecyclobutenes by Formal (2 + 2) Cycloaddition of Allenylsilanes and Internal Alkynes
Source: J Org Chem. 2025 Jul 15;90(29):10555–8. doi: 10.1021/acs.joc.5c01473 (PMC12305637; doi:10.1021/acs.joc.5c01473)
Supplement: Supplementary file 1 [file jo5c01473_si_001.pdf]

Silylium-Ion-Promoted Formation of Methylenecyclobutenes by Formal (2 + 2) Cycloaddition of Allenylsilanes and Internal Alkynes

Lei Deng, Honghua Zuo, Hendrik F. T. Klare, and Martin Oestreich*

Institut für Chemie, Technische Universität Berlin,

Straße des 17. Juni 115, 10623 Berlin, Germany

martin.oestreich@tu-berlin.de

Supporting Information

Table of Contents

1 General Information S3

2 Optimization of the Reaction Conditions[a]...................................... S4

3 Experimental Details for the Substrate Synthesis............................ S7

3.1 Synthesis of Allenylsilanes (GP 1) S7

3.2 Characterization Data for Allenylsilanes **2** S8

3.3 Synthesis of Internal Alkynes.. S10

4 Experimental Details for the Silylium-Ion-Promoted Synthesis of Methylenecyclobutenes from Allenylsilanes and Internal Alkynes...... S11

4.1 General Procedure for the (2 + 2) Cycloaddition (GP 2)................ S11

4.2 Characterization Data for Cycloaddtion Products **10** S11

5 NMR Spectra.. S22

6 References.. S79

1 General Information

All reactions were performed in flame-dried glassware using an MBraun glovebox (O_2 < 0.5 ppm, H_2O < 1.0 ppm) or conventional Schlenk techniques under a static pressure of argon (glovebox) or nitrogen (fume hood) unless otherwise stated. All given elevated temperatures refer to external oil bath temperatures. Standard solvents and reagents were obtained from commercial suppliers and used as received unless otherwise stated. Glassware was dried overnight at 150 °C or flame dried using a heat gun. All plastic syringes and needles used in the glovebox were dried overnight at 60 °C. Liquids and solutions were transferred with syringes. Technical grade solvents for extraction and chromatography were distilled prior to use. Tetrahydrofuran (THF) was dried over sodium and freshly distilled prior to use. Dry benzene (C_6H_6) and *n*-pentane were obtained from an MBraun solvent purification system (SPS-800), degassed by three freeze-pump-thaw cycles, and stored in a glovebox over thermally activated 4 Å molecular sieves. Dichloromethane (CH_2Cl_2), toluene (C_7H_8), fluorobenzene (C_6H_5F), chlorobenzene (C_6H_5Cl), bromobenzene (C_6H_5Br), and 1,2-dichlorobenzene (1,2-$C_6H_4Cl_2$) were dried over CaH_2, distilled, degassed by three freeze-pump-thaw cycles, and stored in a glovebox over thermally activated 4 Å molecular sieves. Silylium carborates [$Me_3Si(HCB_{11}H_5Br_6)$], [$Et_3Si(HCB_{11}H_5Br_6)$], and [*i*$Pr_3Si(HCB_{11}H_5Br_6)$ were synthesized according to reported procedures.[S1] Thin-layer chromatography (TLC) was performed on Macherey-Nagel Alugram® Xtra SIL G/UV254 silica gel 60 pre-coated aluminum-backed plates (200 µm layer thickness). Product spots were visualized under UV light (λ_{max} = 254 nm) and with a ceric ammonium molybdate stain. Column chromatography was performed on Grace 60 (40–63 µm, 230–400 mesh, ASTM) silica gel. ^{1}H, ^{13}C, ^{19}F, and ^{29}Si NMR spectra were recorded in $CDCl_3$ on a Bruker AV400, AV500 instrument, respectively. Chemical shifts are reported in parts per million (ppm) and are referenced to the residual solvent resonance as the internal standard ($CHCl_3$: δ = 7.26 ppm for ^{1}H NMR and $CDCl_3$: δ = 77.20 ppm for ^{13}C NMR). ^{19}F and ^{29}Si NMR spectra are referenced in compliance with the unified scale for NMR chemical shifts as recommended by the IUPAC stating the chemical shift relative to CCl_3F and TMS, respectively.[S2] Data are reported as follows: chemical shift, multiplicity (s = singlet, d = doublet, t = triplet, q = quartet, sept = septet, m = multiplet, br = broad), coupling constants (Hz), and integration. Infrared (IR) spectra were recorded on an Agilent Technologies Cary 630, and the signals are reported in wavenumbers (cm^{-1}). High resolution mass spectra (HRMS) were obtained from the Center for Mass Spectrometry at the Institut für Chemie, Technische Universität Berlin on a Thermo Fisher Scientific LTQ Orbitrap XL apparatus using APCI, ESI or LIFDI techniques with a linear ion trap analyzer.

2 Optimization of the Reaction Conditions[a,b]

Table S1. Effect of the initiator

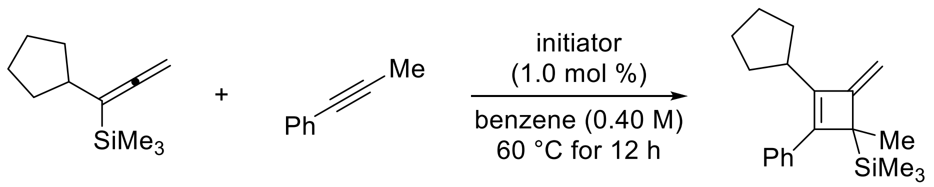

2a **1a** (1.25 equiv) **10aa**

entry	initiator	yield (%)
1	[$Me_3Si(HCB_{11}H_5Br_6)$]	60
2	[$Et_3Si(HCB_{11}H_5Br_6)$]	43
3	[$i Pr_3Si(HCB_{11}H_5Br_6)$]	55
4	[Et_3Si(toluene)][$B(C_6F_5)_4$]	15
5	$AlCl_3$	3
6	TfOH	–
7	[$Me_3Si(HCB_{11}H_5Br_6)$][c]	57
8	[$Me_3Si(HCB_{11}H_5Br_6)$][d]	6

[a]All reactions were performed on a 0.20 mmol scale under an argon atmosphere. [b]Yields were determined by ^1H NMR spectroscopy using CH_2Br_2 as an internal standard. [c]2.0 mol % of [$Me_3Si(HCB_{11}H_5Br_6)$] was used. [d]0.5 mol % of [$Me_3Si(HCB_{11}H_5Br_6)$] was used.

Table S2. Effect of the solvent

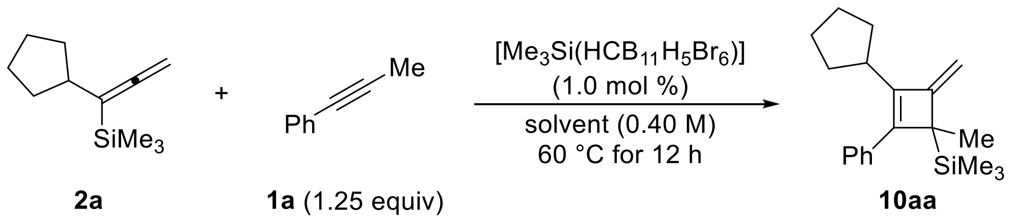

entry	solvent	yield (%)
1	PhH	60
2	toluene	31
3	PhCl	51
4	PhF	47
5	PhBr	–
6	$PhCF_3$	7
7	DCM	5
8	Hexane	–
9	*p*-xylene	25
10	PhH[e]	51

[a]All reactions were performed on a 0.20 mmol scale under an argon atmosphere. [b]Yields were determined by ^{1}H NMR spectroscopy using CH_2Br_2 as an internal standard. [e]PhH (0.30 mL) was used.

Table S3. Effect of the temperature

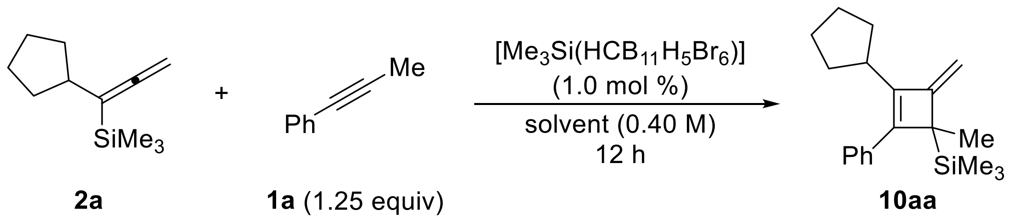

entry	temperature	yield (%)
1	r. t.	31
2	40 °C	29
3	60 °C	60
4	80 °C	46

[a]All reactions were performed on a 0.20 mmol scale under an argon atmosphere. [b]Yields were determined by ^{1}H NMR spectroscopy using CH_2Br_2 as an internal standard.

Table S4. Effect of the reaction time

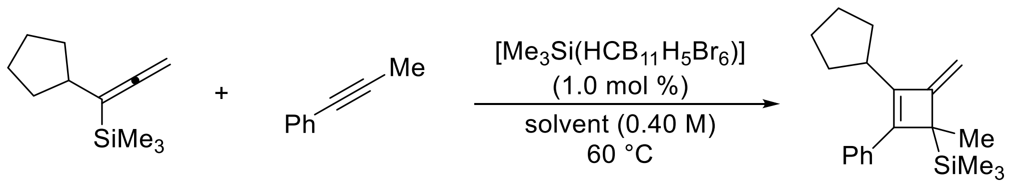

2a **1a** (1.25 equiv) **10aa**

entry	time	yield (%)
1	4 h	30
2	6 h	60
3	12 h	60
4	14 h	60

[a]All reactions were performed on a 0.20 mmol scale under an argon atmosphere. [b]Yields were determined by ^{1}H NMR spectroscopy using CH_2Br_2 as an internal standard.

Table S5. Effect of the substrate ratio

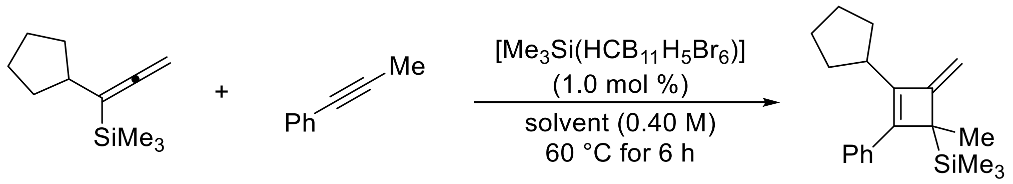

2a **1a** (1.25 equiv) **10aa**

entry	2a (equiv)	1a (equiv)	yield (%)
1	1.10	1.0	62 (55)[f]
2	1.25	1.0	60
3	1.50	1.0	33
4	2.00	1.0	49
5	1.00	2.0	5

[a]All reactions were performed on a 0.20 mmol scale under an argon atmosphere. [b]Yields were determined by ^{1}H NMR spectroscopy using CH_2Br_2 as an internal standard. [f]Isolated yield after flash chromatography on silica gel is given in parentheses.

3 Experimental Details for the Substrate Synthesis

3.1 Synthesis of Allenylsilanes (GP 1)

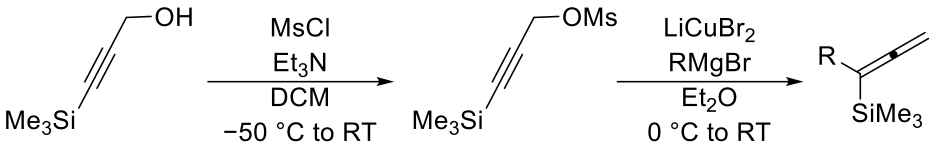

S1 **S2** **2**

Step 1: According to a reported procedure,[S3-6] propargyl alcohol **S1** (10.0 mmol, 1.0 equiv) and Et_3N (15.0 mmol, 1.52 g, 1.5 equiv) are dissolved in CH_2Cl_2 (60 mL) and cooled to −50 °C. Then, MsCl (12.0 mmol, 1.37 g, 1.2 equiv) is added dropwise to this solution. After stirring for 30 min, the resulting mixture is gradually warmed to room temperature and stirred for additional 2 h. Upon completion, the reaction mixture is poured into water (50 mL), and the aqueous phase is extracted with CH_2Cl_2 (3 × 20 mL). The combined organic phases are dried over Na_2SO_4 and concentrated under reduced pressure to afford pure mesylate **S2** as a light-yellow oil, which is directly used in the next step without further purification.

Step 2: A solution of CuBr (12.0 mmol, 1.72 g, 1.2 equiv) and LiBr (12.0 mmol, 1.04 g, 1.2 equiv) in Et_2O (20 mL) is cooled to 0 °C. Freshly prepared RMgBr (12.0 mmol, 1.2 equiv) is then slowly added to this mixture. After stirring at 0 °C for 30 min, mesylate **S2** (10 mmol, 1.0 equiv, dissolved in 5.0 mL of Et_2O) is added, and the resulting mixture is gradually warmed to room temperature and stirred for additional 12 h. Upon completion, the reaction mixture is quenched by the addition of saturated NH_4Cl solution (20 mL), and the aqueous phase is extracted with Et_2O (3 × 15 mL). The combined organic phases are dried over Na_2SO_4 and carefully concentrated under reduced pressure (40 °C, 750 mbar). Purification of the residue by flash column chromatography on silica gel using *n*-pentane as the eluent affords the corresponding allenylsilane **2** as a colorless oil.

3.2 Characterization Data for Allenylsilanes 2

(1-Cyclopentylpropa-1,2-dien-1-yl)trimethylsilane (2a)

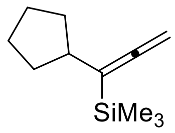

2a

$C_{11}H_{20}Si$

M = 180.37 g/mol

Prepared from Cyclopentylmagnesium bromide according to **GP 1**. Flash column chromatography on silica gel using *n*-pentane as the eluent afforded substrate **2a** as a colorless oil (1.30 g, 72% yield).

R*f* = 0.8 (cyclohexane, stained with $KMnO_4$).

^{1}H NMR (500 MHz, $CDCl_3$, 298 K): *δ* = 4.35 (d, *J* = 2.6 Hz, 2H), 2.31 (quint, *J* = 8.2 Hz, 1H), 1.82–1.76 (m, 2H), 1.71–1.63 (m, 2H), 1.57–1.50 (m, 2H), 1.46–1.39 (m, 2H), 0.11 (s, 9H) ppm.

^{13}C{^{1}H} NMR (126 MHz, $CDCl_3$, 298 K): *δ* = 207.4, 99.5, 69.8, 39.5, 33.5, 25.1, −0.9 ppm.

^{29}Si DEPT NMR (99 MHz, $CDCl_3$, 298 K, optimized for *J* = 7.0 Hz): *δ* = −5.1 ppm.

IR (ATR): ṽ = 2951, 2866, 1925, 1627, 1450, 1403, 1315, 1246, 1017, 862, 833, 800, 751, 690 cm^{-1}.

HRMS (APCI): calculated for $C_{11}H_{21}Si^+$ [M+H]$^+$: 181.1413; Found: 181.1403.

(1-Cyclohexylpropa-1,2-dien-1-yl)trimethylsilane (2b)

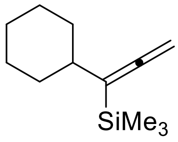

2b

$C_{12}H_{22}Si$

M = 194.39 g/mol

Prepared from Cyclohexylmagnesium bromide according to **GP 1**. Flash column chromatography on silica gel using *n*-pentane as the eluent afforded substrate **2b** as a colorless oil (1.36 g, 70% yield).

R*f* = 0.8 (cyclohexane, stained with $KMnO_4$).

^{1}H NMR (500 MHz, $CDCl_3$, 298 K): *δ* = 4.34 (d, *J* = 2.0 Hz, 2H), 1.75–1.72 (m, 5H), 1.28−1.21 (m, 2H), 1.21–1.15 (m, 4H), 0.10 (s, 9H) ppm.

$^{13}C\{^{1}H\}$ NMR (126 MHz, $CDCl_3$, 298 K): δ = 208.4, 100.7, 69.8, 43.7, 38.1, 34.0, 30.4, 27.1, 26.9, 26.4, −0.9 ppm.

^{29}Si DEPT NMR (99 MHz, $CDCl_3$, 298 K, optimized for J = 7.0 Hz): δ = −5.3 ppm.

IR (ATR): ṽ = 2922, 2851, 1921, 1624, 1447, 1403, 1246, 1023, 980, 846, 834, 801, 753, 690 cm^{-1}.

HRMS (APCI): calculated for $C_{12}H_{23}Si^+$ $[M+H]^+$: 195.1564; Found: 195.1562.

(1-Cycloheptylpropa-1,2-dien-1-yl)trimethylsilane (2c)

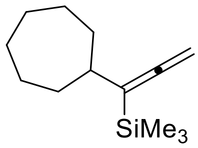

2C
$C_{13}H_{24}Si$
M = 208.42 g/mol

Prepared from Cycloheptylmagnesium bromide according to **GP 1**. Flash column chromatography on silica gel using *n*-pentane as the eluent afforded substrate **2c** as a colorless oil (1.35 g, 65% yield).

R_f = 0.8 (cyclohexane, stained with $KMnO_4$).

^{1}H NMR (500 MHz, $CDCl_3$, 298 K): δ = 4.34 (d, J = 1.8 Hz, 2H), 2.01–1.96 (m, 1H), 1.81–1.76 (m, 2H), 1.69–1.65 (m, 3H), 1.59–1.56 (m, 3H), 1.43–1.41 (m, 3H), 1.28–1.20 (m, 2H), 0.10 (s, 9H) ppm.

$^{13}C\{^{1}H\}$ NMR (126 MHz, $CDCl_3$, 298 K): δ = 208.2, 101.8, 69.9, 46.6, 40.1, 35.8, 31.6, 28.3, 28.0, 26.7, −1.0 ppm.

^{29}Si DEPT NMR (99 MHz, $CDCl_3$, 298 K, optimized for J = 7.0 Hz): δ = −5.2 ppm.

IR (ATR): ṽ = 2917, 2852, 1922, 1622, 1458, 1246, 1035, 987, 834, 802, 753, 690 cm^{-1}.

HRMS (APCI): calculated for $C_{13}H_{25}Si^+$ $[M+H]^+$: 209.1720; Found: 209.1718.

Trimethyl(4-methylpenta-1,2-dien-3-yl)silane (2d)

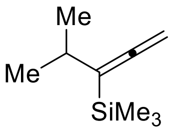

2d
$C_9H_{18}Si$
M = 154.33 g/mol

Prepared from isopropylmagnesium bromide according to **GP 1**. Flash column chromatography on silica gel using *n*-pentane as the eluent afforded substrate **2d** as a colorless oil (1.03 g, 67% yield).

R*f* = 0.8 (cyclohexane, stained with $KMnO_4$).

^{1}H NMR (500 MHz, $CDCl_3$, 298 K): δ = 4.36 (d, J = 2.1 Hz, 2H), 2.23–2.17 (m, 1H), 1.05 (d, J = 6.7 Hz, 6H), 0.11 (s, 9H) ppm.

^{13}C{^{1}H} NMR (126 MHz, $CDCl_3$, 298 K): δ = 207.9, 101.8, 70.3, 28.4, 23.6, −0.9 ppm.

^{29}Si DEPT NMR (99 MHz, $CDCl_3$, 298 K, optimized for J = 7.0 Hz): δ = −5.3 ppm.

IR (ATR): $\tilde{\nu}$ = 2958, 2859, 1921, 1627, 1592, 1456, 1380, 1247, 1167, 1057, 988, 848, 831, 803, 752, 690 cm^{-1}.

HRMS (APCI): calculated for $C_9H_{19}Si^+$ $[M+H]^+$: 155.1251; Found: 155.1251.

3.3 Synthesis of Internal Alkynes

All internal alkynes were already prepared following our earlier work, in which their synthesis and characterization were fully described.[S6]

4 Experimental Details for the Silylium-Ion-Promoted Synthesis of Methylenecyclobutenes from Allenylsilanes and Internal Alkynes

4.1 General Procedure for the (2 + 2) Cycloaddition (GP 2)

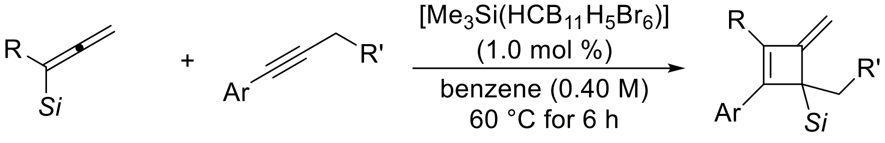

2 **1** **10**

In an argon-filled glovebox, allenylsilane **2** (0.20 mmol, 1.0 equiv) and alkyne **1** (0.22 mmol, 1.10 equiv) are dissolved in benzene (0.5 mL). After stirring for 1 min, silylium carborate [$Me_3Si(HCB_{11}H_5Br_6)$] (1.4 mg, 2.0 µmmol, 1.0 mol %) is added, and the resulting reaction mixture is stirred for additional 6 h at 60 °C. Upon completion of the reaction, the reaction mixture is removed from the glovebox, and all volatiles are evaporated under reduced pressure. CH_2Br_2 (34.8 mg, 0.20 mmol, 1.0 equiv) is subsequently added as an internal standard, and $CDCl_3$ (0.5 mL) is used as the NMR solvent to determine the yield by ^{1}H NMR spectroscopy. Purification by flash column chromatography on silica gel using *n*-pentane as the eluent affords the (2 + 2) cycloaddition product **10**.

4.2 Characterization Data for Cycloaddtion Products 10

(3-Cyclopentyl-1-methyl-4-methylene-2-phenylcyclobut-2-en-1-yl)trimethylsilane (10aa)

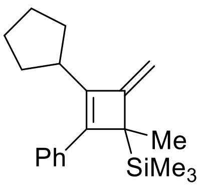

10aa

$C_{20}H_{28}Si$

M = 296.53 g/mol

Prepared from **2a** (36.0 mg, 0.20 mmol) and **1a** (25.6 mg, 0.22 mmol) according to **GP 2**. Flash column chromatography on silica gel using *n*-pentane afforded product **10aa** as a colorless oil (32.6 mg, 55% yield). The reaction on a 1.0 mmol scale afforded **10aa** (157.2 mg, 53% yield).

R*f* = 0.8 (cyclohexane, UV).

^{1}H NMR (500 MHz, $CDCl_3$, 298 K): δ = 7.39 (d, J = 6.8 Hz, 2H), 7.33 (t, J = 6.8 Hz, 2H), 7.25–7.20 (m, 1H), 4.76 (s, 1H), 4.53 (s, 1H), 3.04 (quint, J = 9.8, 9.2 Hz, 1H), 1.94–1.88 (m, 2H), 1.81–1.71 (m, 4H), 1.65–1.60 (m, 2H), 1.51 (s, 3H), −0.02 (s, 9H) ppm.

^{13}C{^{1}H} NMR (126 MHz, $CDCl_3$, 298 K): δ = 154.5, 150.5, 142.9, 136.1, 128.4, 127.4, 127.3, 96.5, 44.5, 38.3, 32.0, 31.3, 26.4, 26.4, 18.9, −2.1 ppm.

^{29}Si DEPT NMR (99 MHz, $CDCl_3$, 298 K, optimized for J = 7.0 Hz): δ = 3.2 ppm.

IR (ATR): ṽ = 3078, 2948, 2865, 2091, 1657, 1591, 1447, 1245, 1071, 866, 833, 760, 747, 691 cm^{-1}.

HRMS (APCI): calculated for $C_{20}H_{29}Si^+$ [M+H]$^+$: 297.2033; Found: 297.2025.

(3-Cyclopentyl-2-(4-fluorophenyl)-1-methyl-4-methylenecyclobut-2-en-1-yl)trimethylsilane (10ba)

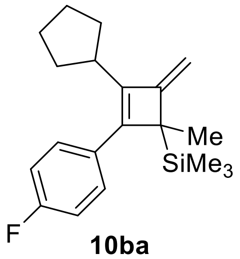

10ba
$C_{20}H_{27}FSi$
M = 314.52 g/mol

Prepared from **2a** (36.0 mg, 0.20 mmol) and **1b** (29.5 mg, 0.22 mmol) according to **GP 2**. Flash column chromatography on silica gel using *n*-pentane afforded product **10aa** as a colorless oil (44.0 mg, 70% yield).

R*f* = 0.70 (cyclohexane, UV).

^{1}H NMR (500 MHz, $CDCl_3$, 298 K): δ = 7.35 (t, J = 7.1 Hz, 2H), 7.06–6.98 (m, 2H), 4.76 (s, 1H), 4.52 (s, 1H), 2.98 (quint, J = 9.4, 9.0 Hz, 1H), 1.93–1.87 (m, 2H), 1.80–1.78 (m, 4H), 1.62 (s, 2H), 1.49 (s, 3H), −0.03 (s, 9H) ppm.

^{13}C{^{1}H} NMR (126 MHz, $CDCl_3$, 298 K): δ = 162.1 (d, $J_{C,F}$ = 245.3 Hz), 154.3, 149.5, 142.5, 132.3 (d, $J_{C,F}$ = 3.4 Hz), 129.1 (d, $J_{C,F}$ = 7.8 Hz), 115.5 (d, J = 21.4 Hz), 96.6, 77.5, 77.2, 76.9, 44.5, 38.3, 32.0, 31.3, 26.4, 26.4, 18.8, −2.1 ppm.

^{19}F NMR (471 MHz, $CDCl_3$, 298 K) δ = −114.0 ppm.

^{29}Si DEPT NMR (99 MHz, $CDCl_3$, 298 K, optimized for J = 7.0 Hz): δ = 3.2 ppm.

IR (ATR): ṽ = 3078, 2951, 2867, 1668, 1598, 1503, 1450, 1407, 1228, 1155, 1051, 833, 737, 688 cm^{-1}.

HRMS (APCI): calculated for $C_{20}H_{28}FSi^+$ $[M+H]^+$: 315.1939; Found: 315.1933.

(2-(4-Chlorophenyl)-3-cyclopentyl-1-methyl-4-methylenecyclobut-2-en-1-yl)trimethylsilane (10ca)

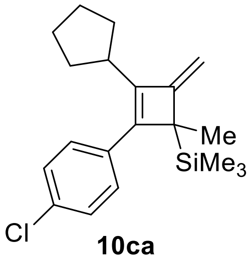

10ca
$C_{20}H_{27}ClSi$
M = 330.97 g/mol

Prepared from **2a** (36.0 mg, 0.20 mmol) and **1c** (33.0 mg, 0.22 mmol) according to **GP 2**. Flash column chromatography on silica gel using *n*-pentane afforded product **10ca** as a colorless oil (46.3 mg, 70% yield).

$\mathbf{R}_f$ = 0.70 (cyclohexane, UV).

^{1}H NMR (500 MHz, $CDCl_3$, 298 K): δ = 7.32 (s, 4H), 4.81 (s, 1H), 4.57 (s, 1H), 3.01 (quint, J = 8.0 Hz, 1H), 1.95–1.90 (m, 2H), 1.79 (s, 4H), 1.64 (s, 2H), 1.51 (s, 3H), 0.00 (s, 9H) ppm.

^{13}C{^{1}H} NMR (126 MHz, $CDCl_3$, 298 K): δ = 154.2, 149.3, 143.5, 134.5, 133.0, 128.7, 128.6, 97.1, 44.5, 38.4, 32.0, 31.3, 26.4, 26.4, 18.8, −2.1 ppm.

^{29}Si DEPT NMR (99 MHz, $CDCl_3$, 298 K, optimized for J = 7.0 Hz): δ = 3.3 ppm.

IR (ATR): ṽ = 3078, 2947, 2864, 1658, 1589, 1486, 1449, 1398, 1245, 1092, 1011, 828, 748, 687 cm^{-1}.

HRMS (APCI): calculated for $C_{20}H_{28}ClSi^+$ $[M+H]^+$: 331.1643; Found: 331.1638.

(2-(4-Bromophenyl)-3-cyclopentyl-1-methyl-4-methylenecyclobut-2-en-1-yl)trimethylsilane (10da)

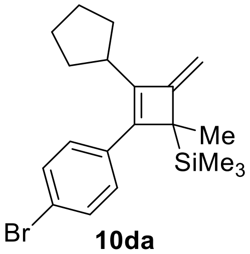

10da
$C_{20}H_{27}BrSi$
M = 374.43 g/mol

Prepared from **2a** (36.0 mg, 0.20 mmol) and **1d** (42.7 mg, 0.22 mmol) according to **GP 2**. Flash column chromatography on silica gel using *n*-pentane afforded product **10da** as a colorless oil (44.9 mg, 60% yield).

R*f* = 0.70 (cyclohexane, UV).

^{1}H NMR (500 MHz, $CDCl_3$, 298 K): δ = 7.44 (d, J = 8.9 Hz, 2H), 7.23 (d, J = 8.7 Hz, 2H), 4.78 (s, 1H), 4.54 (s, 1H), 2.97 (quint, J = 8.4 Hz, 1H), 1.92–1.85 (m, 2H), 1.76 (s, 4H), 1.62 (s, 2H), 1.47 (s, 3H), −0.03 (s, 9H) ppm.

^{13}C{^{1}H} NMR (126 MHz, $CDCl_3$, 298 K): δ = 154.2, 149.3, 143.7, 134.9, 131.6, 128.9, 121.2, 97.2, 44.4, 38.4, 32.0, 31.3, 26.4, 26.3, 18.8, −2.1 ppm.

^{29}Si DEPT NMR (99 MHz, $CDCl_3$, 298 K, optimized for J = 7.0 Hz): δ = 3.3 ppm.

IR (ATR): ṽ = 3078, 2948, 2865, 1658, 1583, 1482, 1450, 1394, 1245, 1071, 1008, 828, 734, 685 cm^{-1}.

HRMS (APCI): calculated for $C_{20}H_{28}{}^{79}BrSi^+$ [M+H]$^+$: 375.1138; Found: 375.1130, $C_{20}H_{28}{}^{81}BrSi^+$ [M+H]$^+$: 377.1118; Found: 377.1109.

(2-([1,1'-Biphenyl]-4-yl)-3-cyclopentyl-1-methyl-4-methylenecyclobut-2-en-1-yl)trimethylsilane (10fa)

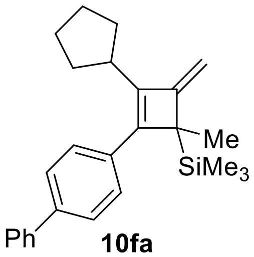

10fa
$C_{26}H_{32}Si$
M = 372.63 g/mol

Prepared from **2a** (36.0 mg, 0.20 mmol) and **1f** (42.3 mg, 0.22 mmol) according to **GP 2**. Flash column chromatography on silica gel using *n*-pentane afforded product **10fa** as a colorless oil (20.9 mg, 28% yield).

R*f* = 0.80 (cyclohexane, UV).

^{1}H NMR (500 MHz, $CDCl_3$, 298 K): δ = 7.61 (d, J = 7.6 Hz, 2H), 7.57 (d, J = 8.2 Hz, 2H), 7.48–7.42 (m, 4H), 7.34 (t, J = 7.4 Hz, 1H), 4.78 (s, 1H), 4.54 (s, 1H), 3.09 (quint, J = 8.2 Hz, 1H), 1.97–1.90 (m, 2H), 1.81–1.72 (m, 4H), 1.67–1.63 (m, 2H), 1.53 (s, 4H), 0.01 (s, 9H) ppm.

^{13}C{^{1}H} NMR (126 MHz, $CDCl_3$, 298 K): δ = 154.5, 150.0, 143.1, 140.9, 139.9, 135.0, 129.0, 127.8, 127.5, 127.1, 127.1, 96.7, 44.3, 38.5, 32.1, 31.3, 26.4, 26.4, 18.8, −2.0 ppm.

^{29}Si DEPT NMR (99 MHz, $CDCl_3$, 298 K, optimized for J = 7.0 Hz): δ = 3.3 ppm.

IR (ATR): ṽ = 3078, 2948, 2865, 1656, 1599, 1485, 1448, 1402, 1245, 1111, 1006, 906, 836, 729, 694 cm^{-1}.

HRMS (APCI): calculated for $C_{26}H_{33}Si^+$ [M+H]$^+$: 373.2346; Found: 373.2339.

(3-Cyclopentyl-2-(2-fluorophenyl)-1-methyl-4-methylenecyclobut-2-en-1-yl)trimethylsilane (10ha)

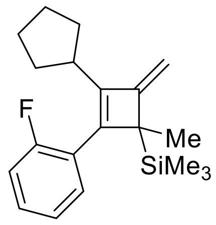

10ha
$C_{20}H_{27}FSi$
M = 314.52 g/mol

Prepared from **2a** (36.0 mg, 0.20 mmol) and **1h** (29.5 mg, 0.22 mmol) according to **GP 2**. Flash column chromatography on silica gel using *n*-pentane afforded product **10ha** as a colorless oil (25.2 mg, 40% yield).

R*f* = 0.70 (cyclohexane, UV).

^{1}H NMR (500 MHz, $CDCl_3$, 298 K): δ = 7.30–7.27 (m, 1H), 7.25–7.19 (m, 1H), 7.11–7.02 (m, 2H), 4.76 (s, 1H), 4.53 (s, 1H), 2.77 (quint, J = 7.9, 7.4 Hz, 1H), 1.93–1.87 (m, 1H), 1.80–1.78 (m, 1H), 1.74–1.66 (m, 4H), 1.64–1.57 (m, 2H), 1.48 (d, J = 1.9 Hz, 3H), −0.09 (s, 9H) ppm.

$^{13}C\{^1H\}$ NMR (126 MHz, $CDCl_3$, 298 K): δ = 159.4 (d, $J_{C,F}$ = 248.5 Hz), 154.3, 146.1, 145.3, 129.7 (d, $J_{C,F}$ = 4.3 Hz), 129.1 (d, $J_{C,F}$ = 8.0 Hz), 124.0 (d, $J_{C,F}$ = 17.0 Hz), 123.8 (d, $J_{C,F}$ = 3.5 Hz), 116.0 (d, $J_{C,F}$ = 22.9 Hz), 45.8, 38.6 (d, $J_{C,F}$ = 3.4 Hz), 31.7, 31.4, 26.3, 19.2 (d, $J_{C,F}$ = 3.8 Hz), -2.7 ppm.

^{19}F NMR (471 MHz, $CDCl_3$, 298 K) δ = −109.7 ppm.

^{29}Si DEPT NMR (99 MHz, $CDCl_3$, 298 K, optimized for J = 7.0 Hz): δ = 3.3 ppm.

IR (ATR): ṽ = 3077, 2952, 2867, 1612, 1487, 1247, 1110, 1033, 838, 757, 690 cm^{-1}.

HRMS (APCI): calculated for $C_{20}H_{27}FSi^+$ $[M+H]^+$: 315.1939; Found: 315.1932.

(2-(2-Chlorophenyl)-3-cyclopentyl-1-methyl-4-methylenecyclobut-2-en-1-yl)trimethylsilane (10ia)

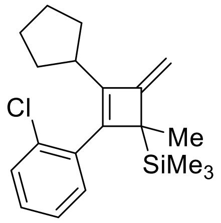

10ia
$C_{20}H_{27}ClSi$
M = 330.97 g/mol

Prepared from **2a** (36.0 mg, 0.20 mmol) and **1i** (33.0 mg, 0.22 mmol) according to **GP 2**. Flash column chromatography on silica gel using *n*-pentane afforded product **10ia** as a colorless oil (29.8 mg, 45% yield).

R_f = 0.70 (cyclohexane, UV).

^1H NMR (500 MHz, $CDCl_3$, 298 K): δ =7.44–7.42 (m, 1H), 7.31–7.29 (m, 1H), 7.27–7.21 (m, 2H), 4.80 (s, 1H), 4.58 (s, 1H), 2.70 (quint, J = 8.6 Hz, 1H), 1.97–1.92 (dm, 1H), 1.86–1.80 (m, 1H), 1.70–1.61 (m, 4H), 1.58–1.55 (m, 2H), 1.51 (s, 3H), 0.00 (s, 9H) ppm.

$^{13}C\{^1H\}$ NMR (126 MHz, $CDCl_3$, 298 K): δ = 154.3, 148.2, 146.3, 135.6, 132.7, 130.0, 129.6, 128.6, 126.2, 96.3, 47.4, 39.0, 31.4, 30.7, 26.1, 19.5, −2.6 ppm.

^{29}Si DEPT NMR (99 MHz, $CDCl_3$, 298 K, optimized for J = 7.0 Hz): δ = 3.1 ppm.

IR (ATR): ṽ = 3069, 2949, 2865, 1669, 1450, 1246, 1057, 833, 742, 707 cm^{-1}.

HRMS (APCI): calculated for $C_{20}H_{28}ClSi^+$ $[M+H]^+$: 331.1643; Found: 331.1638.

(3-Cyclopentyl-1-methyl-4-methylene-2-(o-tolyl)cyclobut-2-en-1-yl)trimethylsilane (10ja)

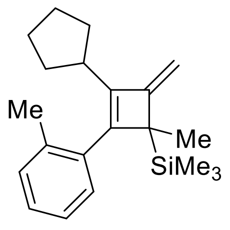

10ja
$C_{21}H_{30}Si$
M = 310.56 g/mol

Prepared from **2a** (36.0 mg, 0.20 mmol) and **1j** (28.6 mg, 0.22 mmol) according to **GP 2**. Flash column chromatography on silica gel using *n*-pentane afforded product **10ja** as a colorless oil (23.6 mg, 38% yield).

R_f = 0.80 (cyclohexane, UV).

^{1}H NMR (500 MHz, $CDCl_3$, 298 K): δ = 7.21–7.11 (m, 4H), 4.70 (s, 1H), 4.49 (s, 1H), 2.53 (quint, J = 8.4 Hz, 1H), 2.34 (s, 3H), 1.83–1.73 (m, 2H), 1.66–1.56 (m, 4H), 1.53–1.48 (m, 2H), 1.41 (s, 3H), −0.03 (s, 9H) ppm.

^{13}C{^{1}H} NMR (126 MHz, $CDCl_3$, 298 K): δ = 154.4, 151.6, 144.5, 136.0, 135.8, 130.3, 128.1, 127.3, 125.2, 95.3, 47.5, 38.5, 31.4, 30.9, 26.0, 21.0, 19.4, −2.4 ppm.

^{29}Si DEPT NMR (99 MHz, $CDCl_3$, 298 K, optimized for J = 7.0 Hz): δ = 2.8 ppm.

IR (ATR): ṽ = 3068, 2951, 2866, 1668, 1450, 1246, 1047, 906, 835, 729 cm^{-1}.

HRMS (APCI): calculated for $C_{21}H_{31}Si^+$ [M+H]$^+$: 311.2190; Found: 311.2183.

(3-Cyclopentyl-2-(3-fluorophenyl)-1-methyl-4-methylenecyclobut-2-en-1-yl)trimethylsilane (10ka)

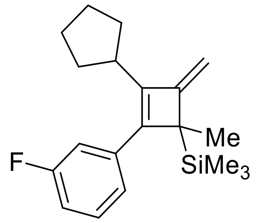

10ka
$C_{20}H_{27}FSi$
M = 314.52 g/mol

Prepared from **2a** (36.0 mg, 0.20 mmol) and **1k** (29.5 mg, 0.22 mmol) according to **GP 2**. Flash column chromatography on silica gel using *n*-pentane afforded product **10ka** as a colorless oil (36.5 mg, 58% yield).

R*f* = 0.70 (cyclohexane, UV).

^{1}H NMR (500 MHz, $CDCl_3$, 298 K): δ = 7.31–7.27 (m, 1H), 7.14 (d, J = 7.8 Hz, 1H), 7.06 (d, J = 9.7 Hz, 1H), 6.92 (t, J = 8.6 Hz, 1H), 4.80 (s, 1H), 4.56 (s, 1H), 3.01 (quint, J = 8.6 Hz, 1H), 1.94–1.89 (m, 3H), 1.77 (s, 4H), 1.63 (s, 2H), 1.49 (s, 3H), −0.02 (s, 9H) ppm.

^{13}C{^{1}H} NMR (126 MHz, $CDCl_3$, 298 K): δ = 162.9 (d, $J_{C,F}$ = 245.2 Hz), 154.2, 149.3, 144.1, 138.1 (d, $J_{C,F}$ = 8.2 Hz), 129.9 (d, $J_{C,F}$ = 8.3 Hz), 123.1 (d, $J_{C,F}$ = 3.1 Hz), 114.0 (d, $J_{C,F}$ = 22.3 Hz), 97.5, 44.6, 38.3, 32.0, 31.3, 26.4, 18.8, −2.1 ppm.

^{19}F NMR (471 MHz, $CDCl_3$, 298 K) δ = −113.2 ppm.

^{29}Si DEPT NMR (99 MHz, $CDCl_3$, 298 K, optimized for J = 7.0 Hz): δ = 3.3 ppm.

IR (ATR): ṽ = 3077, 2948, 2863, 1658, 1606, 1576, 1480, 1438, 1245, 1170, 1089, 833, 781, 748, 688 cm^{-1}.

HRMS (APCI): calculated for $C_{20}H_{28}FSi^+$ [M+H]$^+$: 315.1939; Found: 315.1932.

(2-(3-Chlorophenyl)-3-cyclopentyl-1-methyl-4-methylenecyclobut-2-en-1-yl)trimethylsilane (10la)

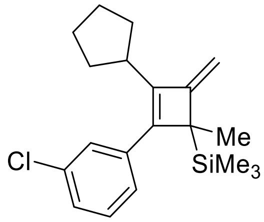

10la

$C_{20}H_{27}ClSi$

M = 330.97 g/mol

Prepared from **2a** (36.0 mg, 0.20 mmol) and **1l** (33.0 mg, 0.22 mmol) according to **GP 2**. Flash column chromatography on silica gel using *n*-pentane afforded product **10la** as a colorless oil (46.3 mg, 70% yield).

R*f* = 0.70 (cyclohexane, UV).

^{1}H NMR (500 MHz, $CDCl_3$, 298 K): δ = 7.37 (s, 1H), 7.28 (s, 2H), 7.22 (dd, J = 4.5, 2.1 Hz, 1H), 4.83 (s, 1H), 4.59 (s, 1H), 3.03 (quint, J = 8.0 Hz, 1H), 1.97–1.89 (m, 2H), 1.80 (s, 4H), 1.66 (s, 2H), 1.52 (s, 3H), 0.01 (s, 9H) ppm.

$^{13}C\{^{1}H\}$ NMR (126 MHz, $CDCl_3$, 298 K): δ = 154.2, 149.0, 144.3, 137.8, 134.4, 129.7, 127.3, 125.4, 97.5, 44.6, 38.3, 32.0, 31.4, 26.4, 26.4, 18.8, −2.1 ppm.

^{29}Si DEPT NMR (99 MHz, $CDCl_3$, 298 K, optimized for J = 7.0 Hz): δ = 3.3 ppm.

IR (ATR): ṽ = 3074, 2948, 2865, 1658, 1587, 1472, 1408, 1245, 1076, 833, 783, 749, 687 cm^{-1}.

HRMS (APCI): calculated for $C_{20}H_{28}ClSi^+$ [M−H]$^+$: 331.1643; Found: 331.1637.

(2-(3-Bromophenyl)-3-cyclopentyl-1-methyl-4-methylenecyclobut-2-en-1-yl)trimethylsilane (10ma)

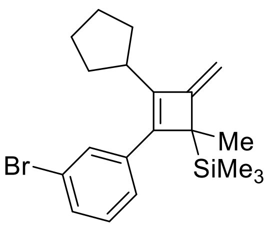

10ma
$C_{20}H_{27}BrSi$
M = 375.43 g/mol

Prepared from **2a** (36.0 mg, 0.20 mmol) and **1m** (42.7 mg, 0.22 mmol) according to **GP 2**. Flash column chromatography on silica gel using *n*-pentane afforded product **10ma** as a colorless oil (37.4 mg, 50% yield).

R_f = 0.70 (cyclohexane, UV).

^{1}H NMR (500 MHz, $CDCl_3$, 298 K): δ = 7.50–7.49 (m, 1H), 7.34 (d, J = 8.1 Hz, 1H), 7.30 (d, J = 7.9 Hz, 1H), 7.19 (t, J = 7.9 Hz, 1H), 4.80 (s, 1H), 4.56 (s, 1H), 2.99 (quint, J = 8.2 Hz, 1H), 1.93–1.86 (m, 2H), 1.80–1.70 (m, 4H), 1.66–1.61 (m, 2H), 1.48 (s, 3H), −0.02 (s, 9H) ppm.

$^{13}C\{^{1}H\}$ NMR (126 MHz, $CDCl_3$, 298 K): δ = 154.1, 148.9, 144.4, 138.1, 130.2, 130.1, 129.9, 125.9, 122.6, 97.6, 44.6, 38.3, 32.0, 31.4, 26.4, 26.4, 18.8, −2.1 ppm.

^{29}Si DEPT NMR (99 MHz, $CDCl_3$, 298 K, optimized for J = 7.0 Hz): δ = 3.3 ppm.

IR (ATR): ṽ = 3075, 2948, 2865, 1657, 1584, 1449, 1406, 1246, 1069, 833, 782, 737, 687 cm^{-1}.

HRMS (APCI): calculated for $C_{20}H_{28}{}^{79}BrSi^+$ [M+H]$^+$: 375.1138; Found: 375.1132, $C_{20}H_{28}{}^{81}BrSi^+$ [M+H]$^+$: 377.1118; Found: 377.1111.

(3-Cyclohexyl-1-methyl-4-methylene-2-phenylcyclobut-2-en-1-yl)trimethylsilane (10ab)

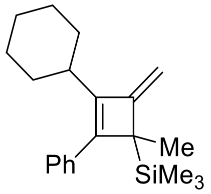

10ab
$C_{21}H_{30}Si$
M = 310.56 g/mol

Prepared from **2b** (38.8 mg, 0.20 mmol) and **1a** (25.6 mg, 0.22 mmol) according to **GP 2**. Flash column chromatography on silica gel using *n*-pentane afforded product **10ab** as a colorless oil (32.9 mg, 53% yield).

R_f = 0.80 (cyclohexane, UV).

^{1}H NMR (500 MHz, $CDCl_3$, 298 K): δ = 7.41–7.33 (m, 4H), 7.29–7.23 (m, 1H), 4.85 (s, 1H), 4.54 (s, 1H), 2.63 (tt, J = 12.2, 3.4 Hz, 1H), 1.89–1.78 (m, 4H), 1.77–1.69 (m, 2H), 1.65–1.59 (m, 1H), 1.53 (s, 3H), 1.33–1.22 (m, 3H), -0.00 (s, 9H) ppm.

^{13}C{^{1}H} NMR (126 MHz, $CDCl_3$, 298 K): δ = 154.8, 149.5, 144.2, 128.4, 127.4, 127.3, 96.9, 44.4, 38.1, 31.7, 31.5, 30.4, 26.7, 26.4, 19.0, −2.1 ppm.

^{29}Si DEPT NMR (99 MHz, $CDCl_3$, 298 K, optimized for J = 7.0 Hz): δ = 3.2 ppm.

IR (ATR): $\tilde{\nu}$ = 3078, 2924, 2851, 1660, 1594, 1446, 1246, 1061, 985, 835, 733, 692 cm^{-1}.

HRMS (APCI): calculated for $C_{21}H_{31}Si^+$ $[M+H]^+$: 311.2190; Found: 311.2183.

(3-Cycloheptyl-1-methyl-4-methylene-2-phenylcyclobut-2-en-1-yl)trimethylsilane (10ac)

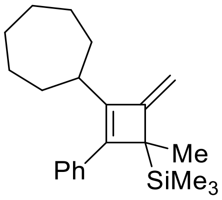

10ac
$C_{22}H_{32}Si$
M = 324.58 g/mol

Prepared from **2c** (41.6 mg, 0.20 mmol) and **1a** (25.6 mg, 0.22 mmol) according to **GP 2**. Flash column chromatography on silica gel using *n*-pentane afforded product **10ac** as a colorless oil (19.5 mg, 30% yield).

R*f* = 0.80 (cyclohexane, UV).

^{1}H NMR (500 MHz, $CDCl_3$, 298 K): δ = 7.36–7.31 (m, 4H), 7.24 – 7.21 (m, 1H), 4.77 (s, 1H), 4.51 (s, 1H), 2.77–2.70 (m, 1H), 1.91–1.86 (m, 2H), 1.80–1.62 (m, 7H), 1.60–1.55 (m, 3H), 1.49 (s, 3H), −0.03 (s, 9H) ppm.

^{13}C{^{1}H} NMR (126 MHz, $CDCl_3$, 298 K): δ = 154.5, 148.0, 145.6, 136.1, 128.4, 127.4, 127.3, 96.9, 44.1, 39.6, 33.7, 33.4, 28.4, 28.0, 27.9, 27.8, 19.1, −2.0 ppm.

^{29}Si DEPT NMR (99 MHz, $CDCl_3$, 298 K, optimized for J = 7.0 Hz): δ = 3.5 ppm.

IR (ATR): ṽ = 3078, 2923, 2854, 1662, 1447, 1368, 1246, 1072, 838, 768, 694 cm^{-1}.

HRMS (APCI): calculated for $C_{22}H_{33}Si^+$ $[M+H]^+$: 325.2346; Found: 325.2335.

(3-Isopropyl-1-methyl-4-methylene-2-phenylcyclobut-2-en-1-yl)trimethylsilane (10ad)

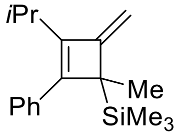

10ad

$C_{18}H_{26}Si$

M = 270.49 g/mol

Prepared from **2d** (30.8 mg, 0.20 mmol) and **1a** (25.6 mg, 0.22 mmol) according to **GP 2**. Flash column chromatography on silica gel using *n*-pentane afforded product **10ad** as a colorless oil (28.1 mg, 52% yield).

R*f* = 0.80 (cyclohexane, UV).

^{1}H NMR (500 MHz, $CDCl_3$, 298 K): δ = 7.38–7.36 (m, 2H), 7.32 (t, J = 7.7 Hz, 2H), 7.23 (td, J = 7.0, 1.4 Hz, 1H), 4.81 (s, 1H), 4.53 (s, 1H), 2.96 (hept, J = 7.0 Hz, 1H), 1.50 (s, 3H), 1.23 (t, J = 6.6 Hz, 6H), −0.02 (s, 9H) ppm.

^{13}C{^{1}H} NMR (126 MHz, $CDCl_3$, 298 K): δ = 154.3, 149.3, 145.0, 136.0, 128.4, 127.5, 127.3, 96.9, 44.3, 27.6, 21.5, 21.4, 18.9, −2.1 ppm.

^{29}Si DEPT NMR (99 MHz, $CDCl_3$, 298 K, optimized for J = 7.0 Hz): δ = 3.3 ppm.

IR (ATR): ṽ = 3079, 2957, 2855, 1660, 1459, 1380, 1245, 1074, 833, 771, 750, 690 cm^{-1}.

HRMS (APCI): calculated for $C_{18}H_{27}Si^+$ $[M+H]^+$: 271.1877; Found: 271.1870.

5 NMR Spectra

Figure S1. ^{1}H NMR (500 MHz, $CDCl_3$, 298 K) of **2a**

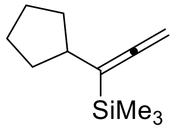

2a

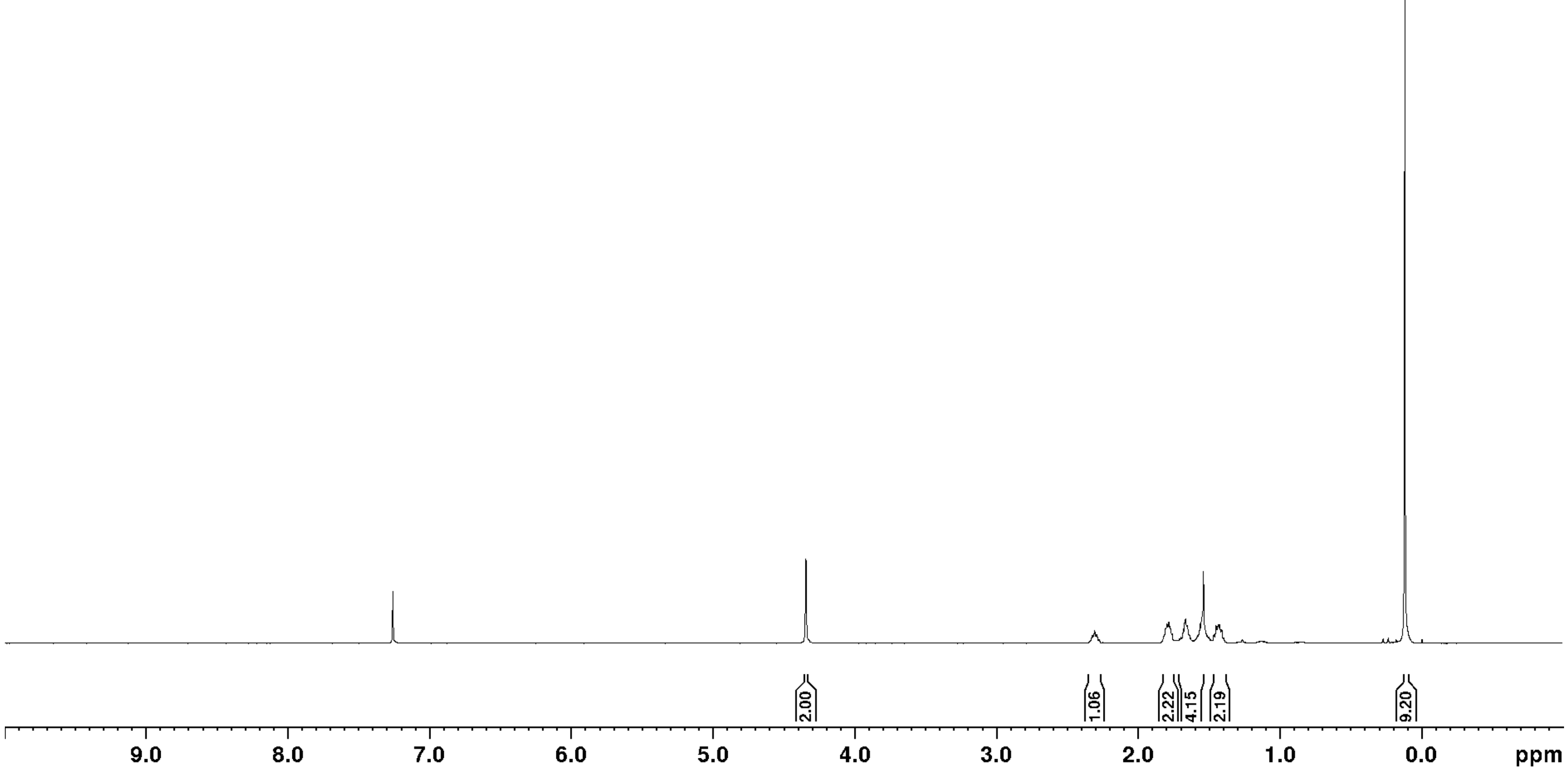

Figure S2. $^{13}C\{^{1}H\}$ NMR (126 MHz, $CDCl_3$, 298 K) of **2a**

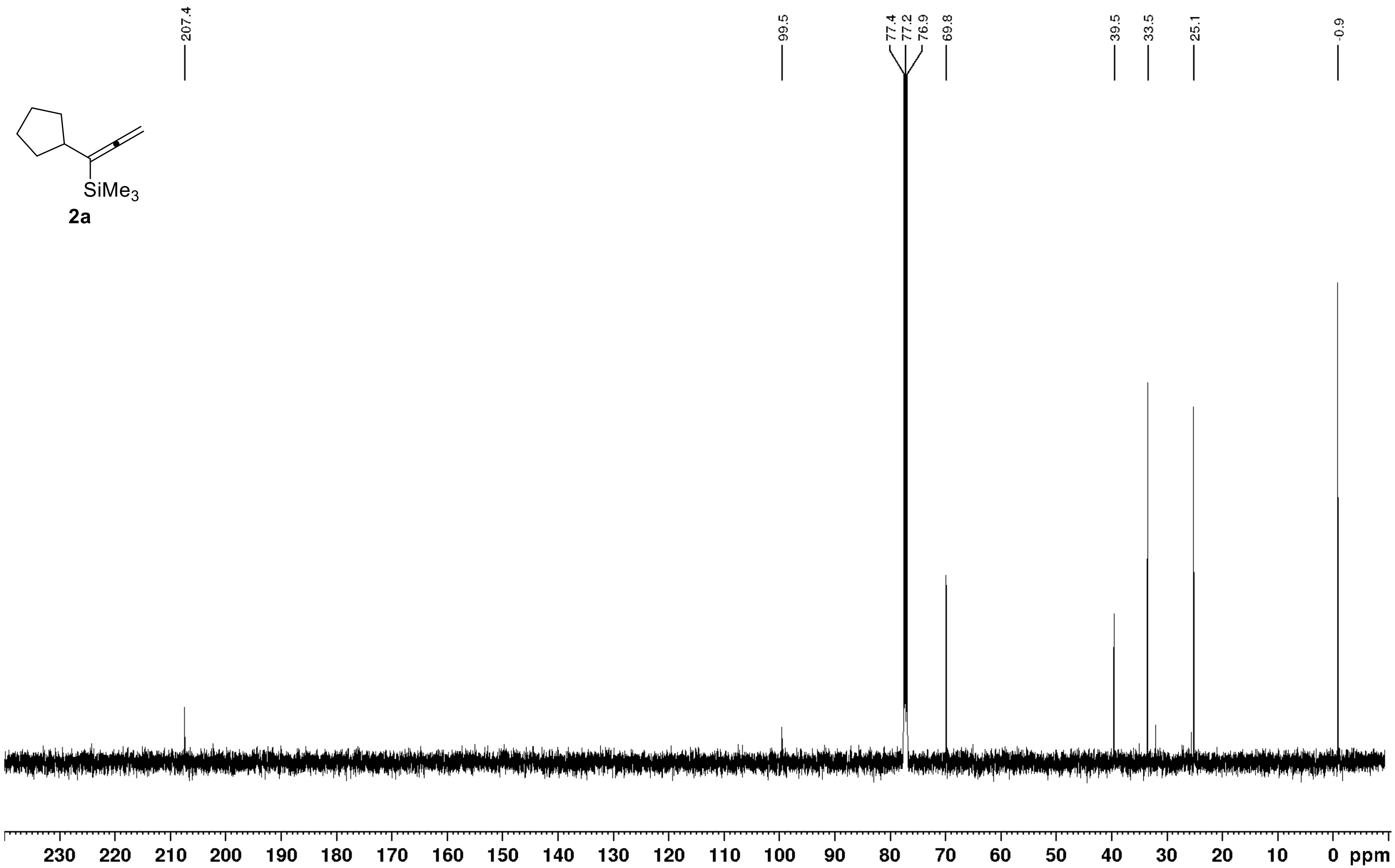

Figure S3. ^{29}Si DEPT NMR (99 MHz, $CDCl_3$, 298 K, optimized for J = 7.0 Hz) of **2a**

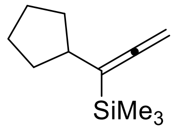

2a

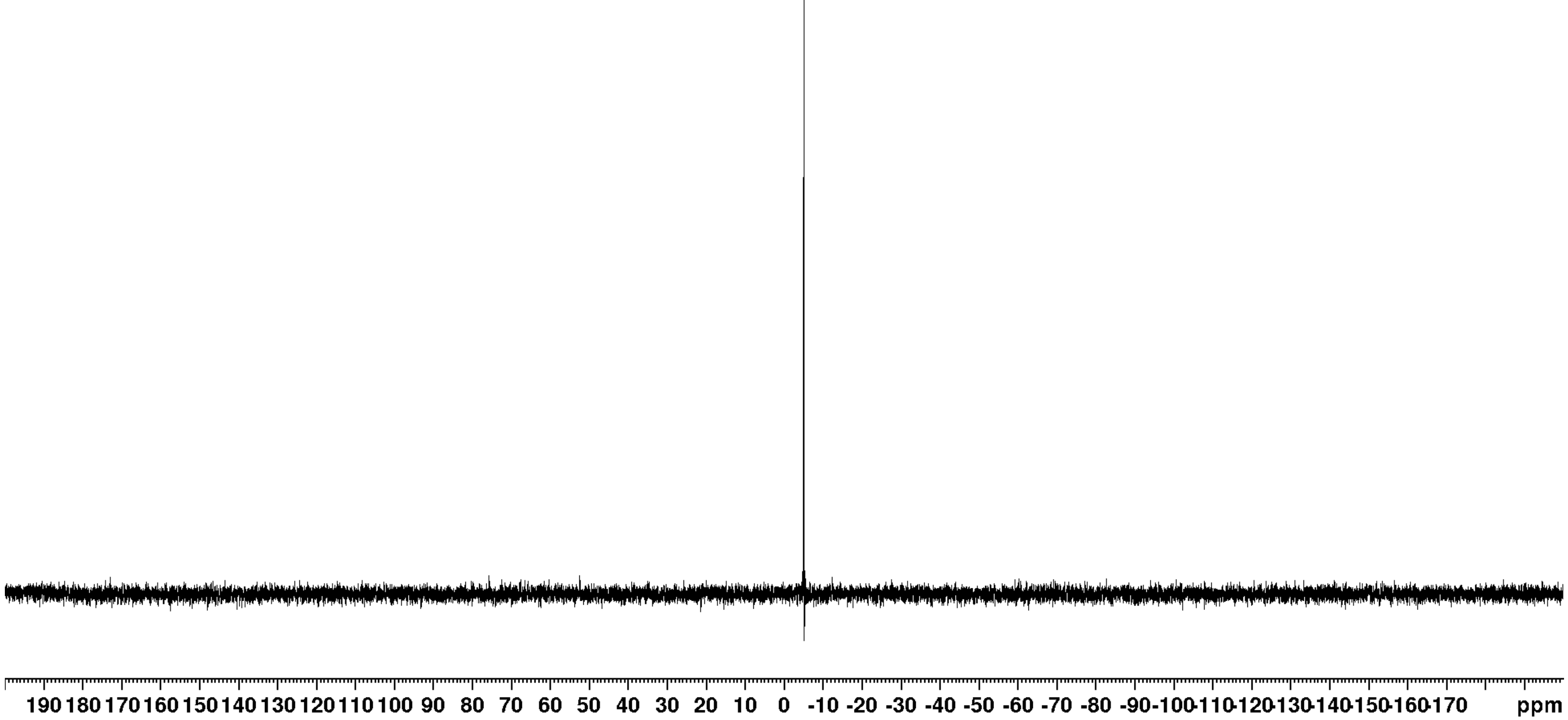

Figure S4. ^{1}H NMR (500 MHz, $CDCl_3$, 298 K) of **2b**

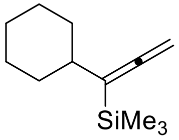

2b

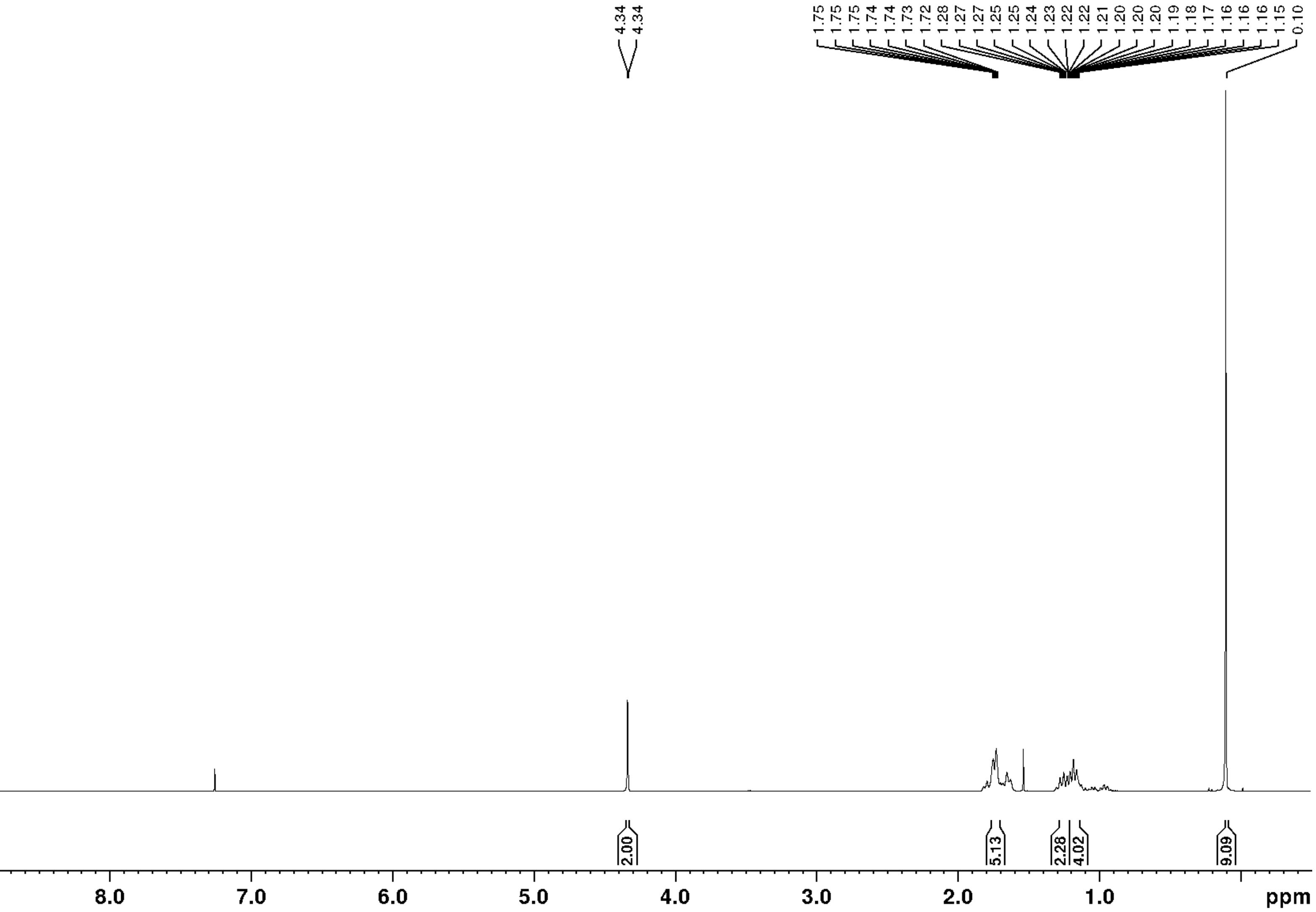

Figure S5. $^{13}C\{^1H\}$ NMR (126 MHz, $CDCl_3$, 298 K) of **2b**

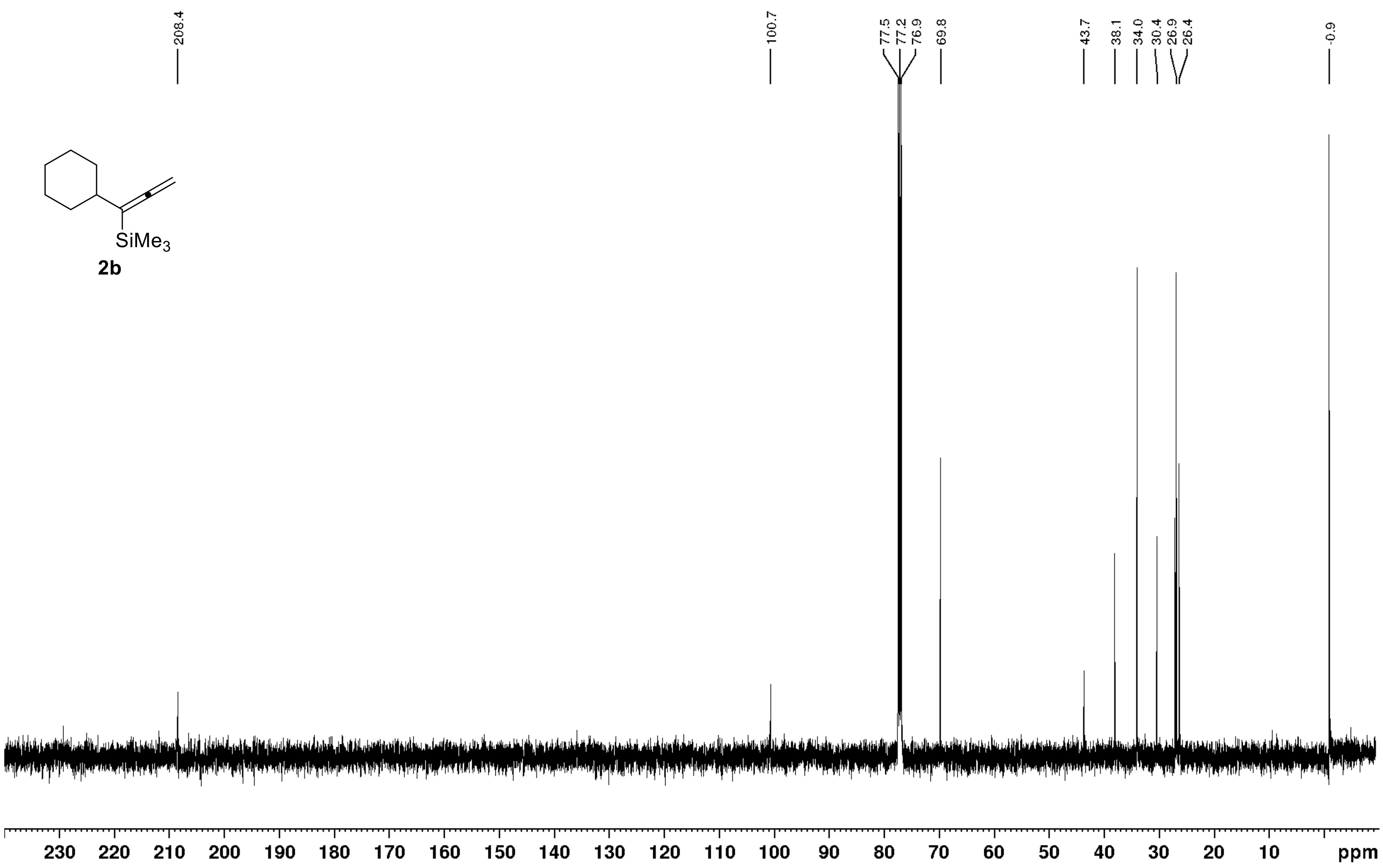

Figure S6. ^{29}Si DEPT NMR (99 MHz, $CDCl_3$, 298 K, optimized for J = 7.0 Hz) of **2b**

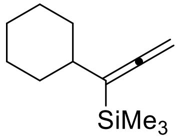

2b

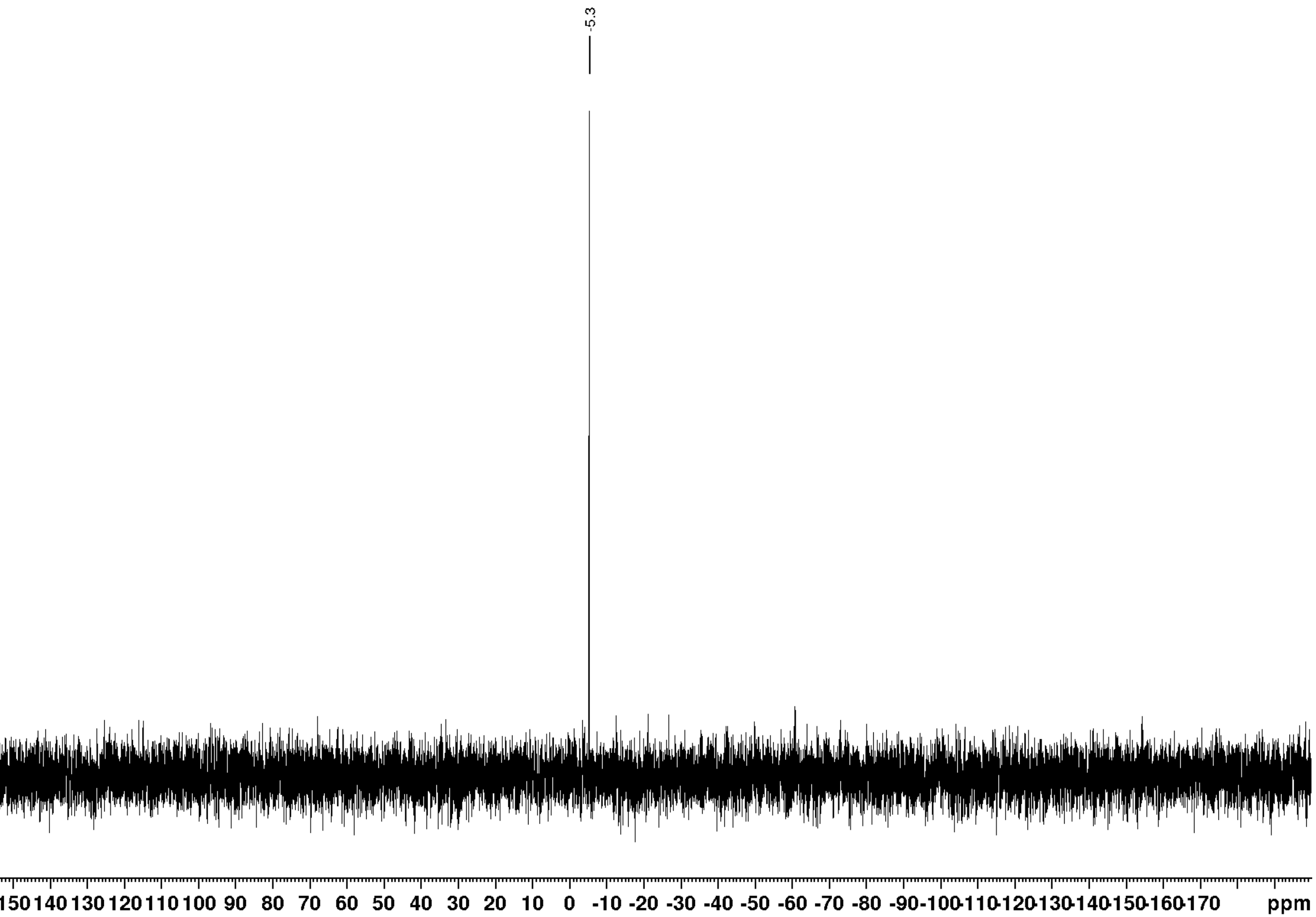

Figure S7. ^{1}H NMR (500 MHz, $CDCl_3$, 298 K) of **2c**

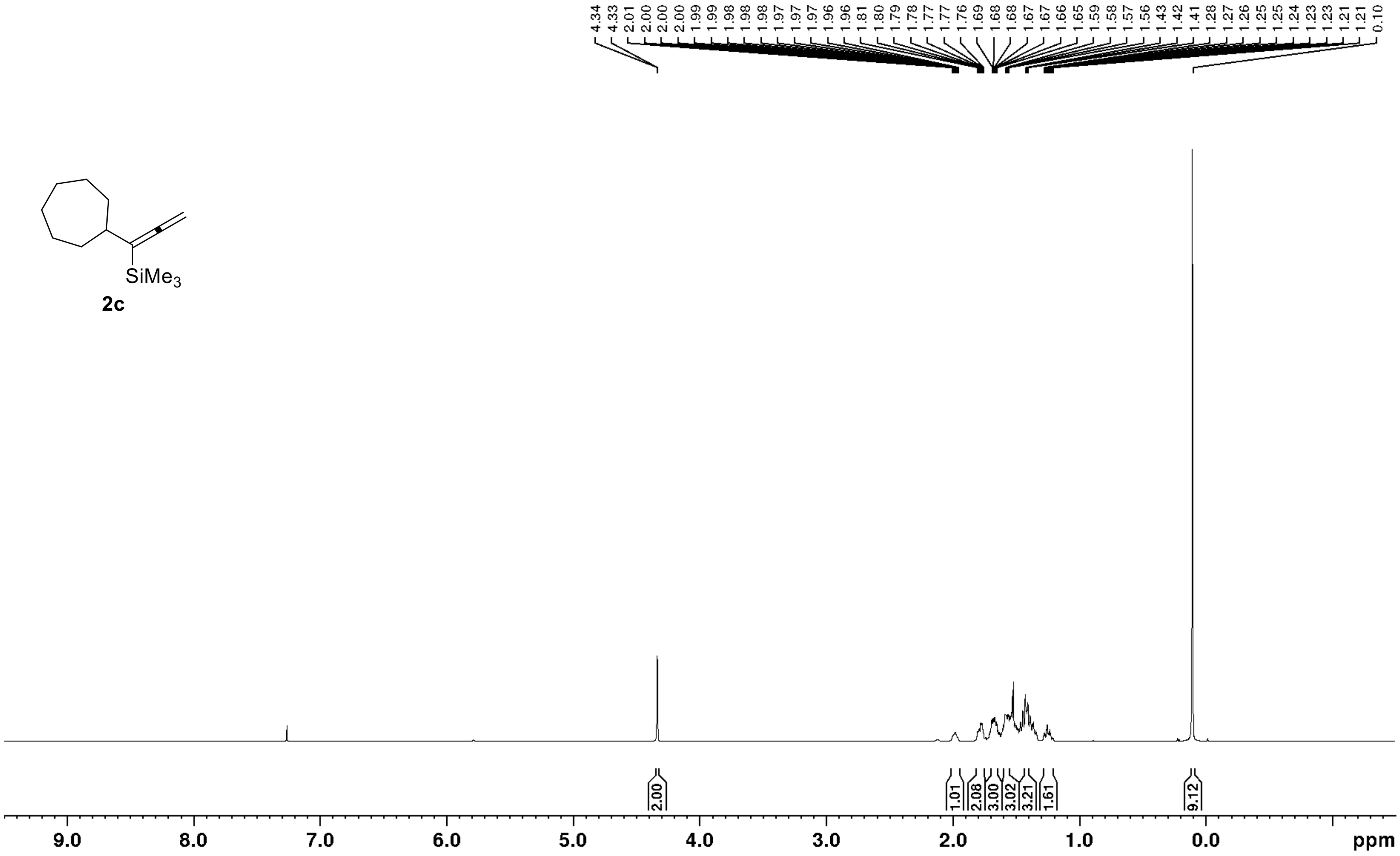

Figure S8. $^{13}C\{^{1}H\}$ NMR (126 MHz, $CDCl_3$, 298 K) of **2c**

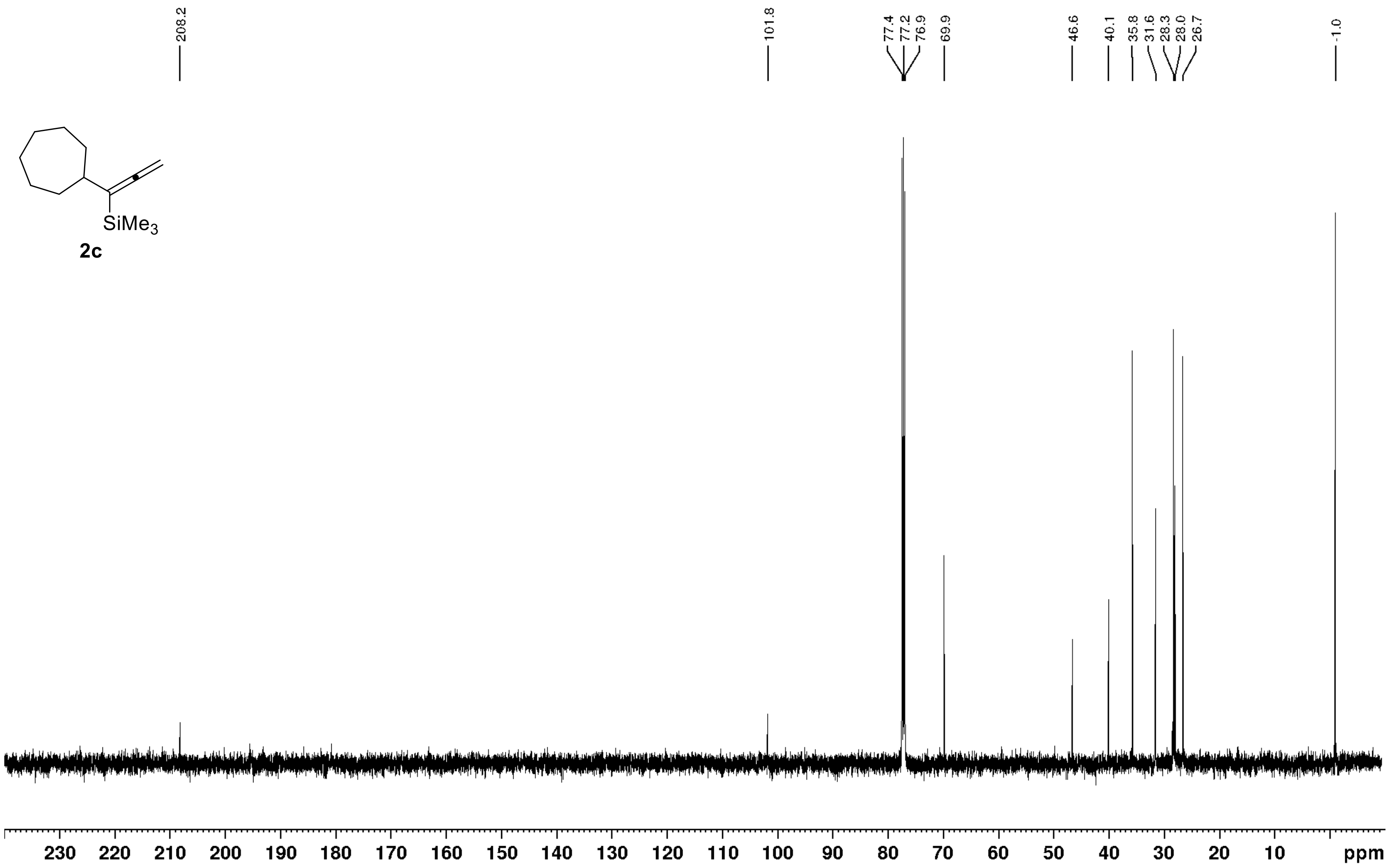

Figure S9. ^{29}Si DEPT NMR (99 MHz, $CDCl_3$, 298 K, optimized for J = 7.0 Hz) of **2c**

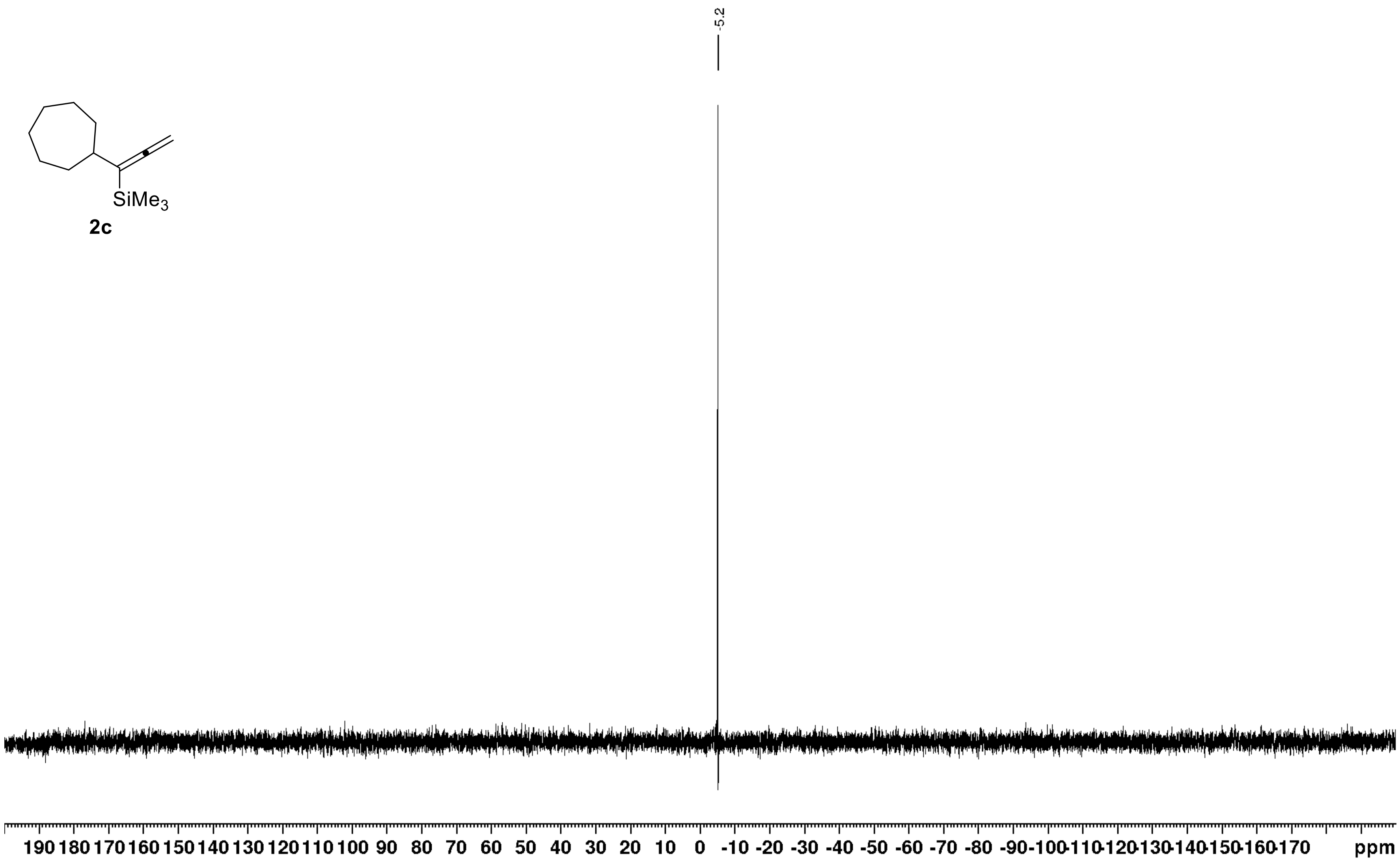

Figure S10. ^{1}H NMR (500 MHz, $CDCl_3$, 298 K) of **2d**

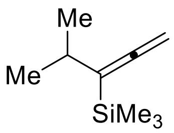

2d

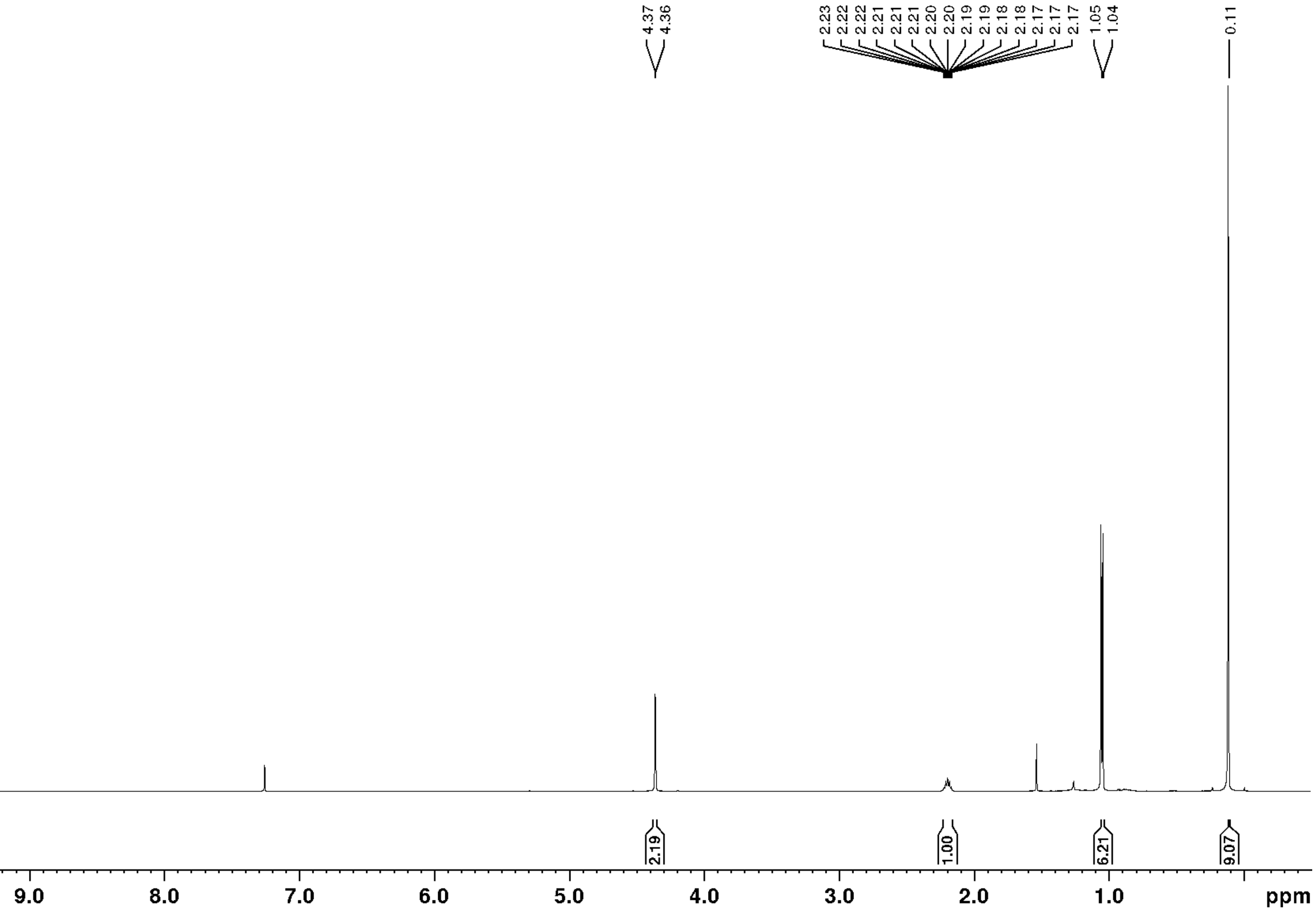

Figure S11. $^{13}C\{^{1}H\}$ NMR (126 MHz, $CDCl_3$, 298 K) of **2d**

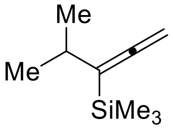

2d

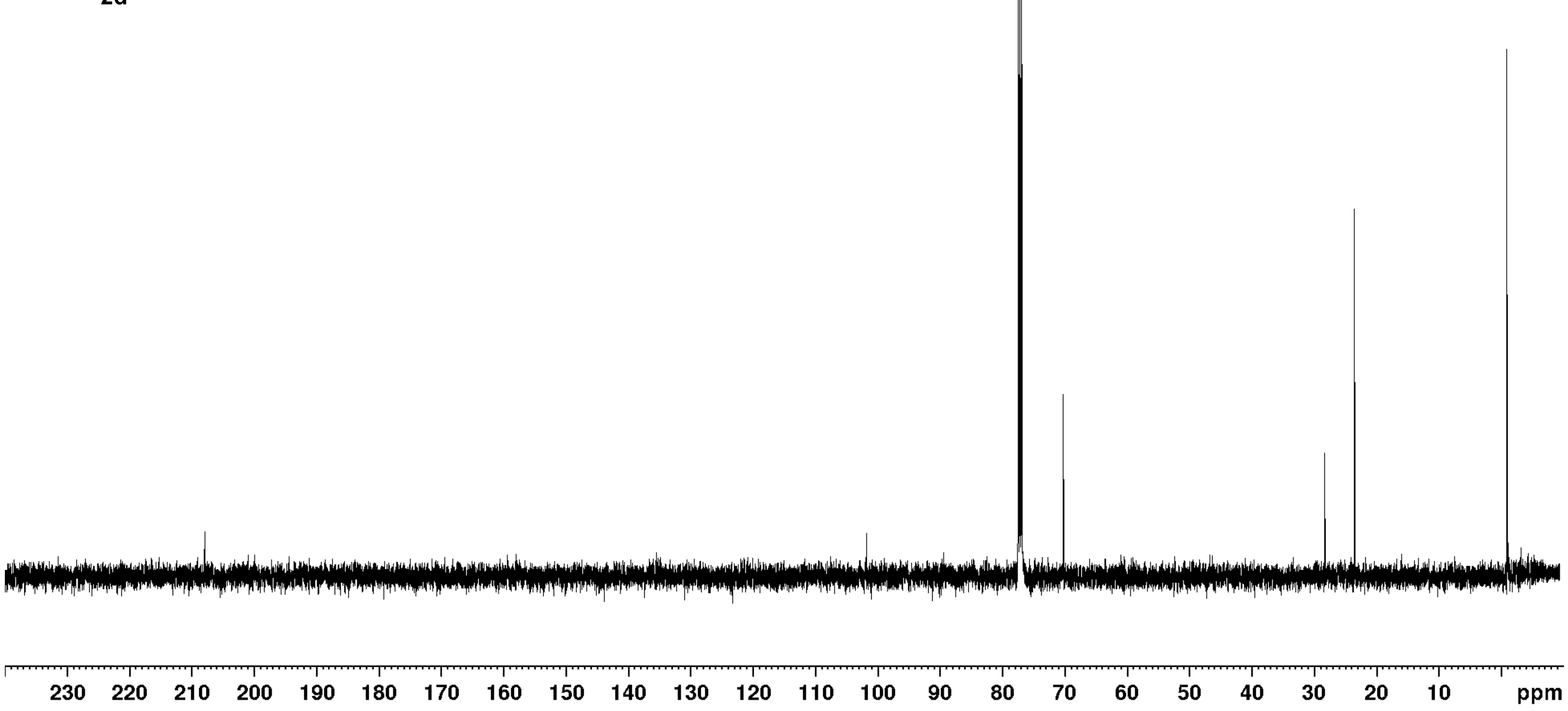

Figure S12. ^{29}Si DEPT NMR (99 MHz, $CDCl_3$, 298 K, optimized for J = 7.0 Hz) of **2d**

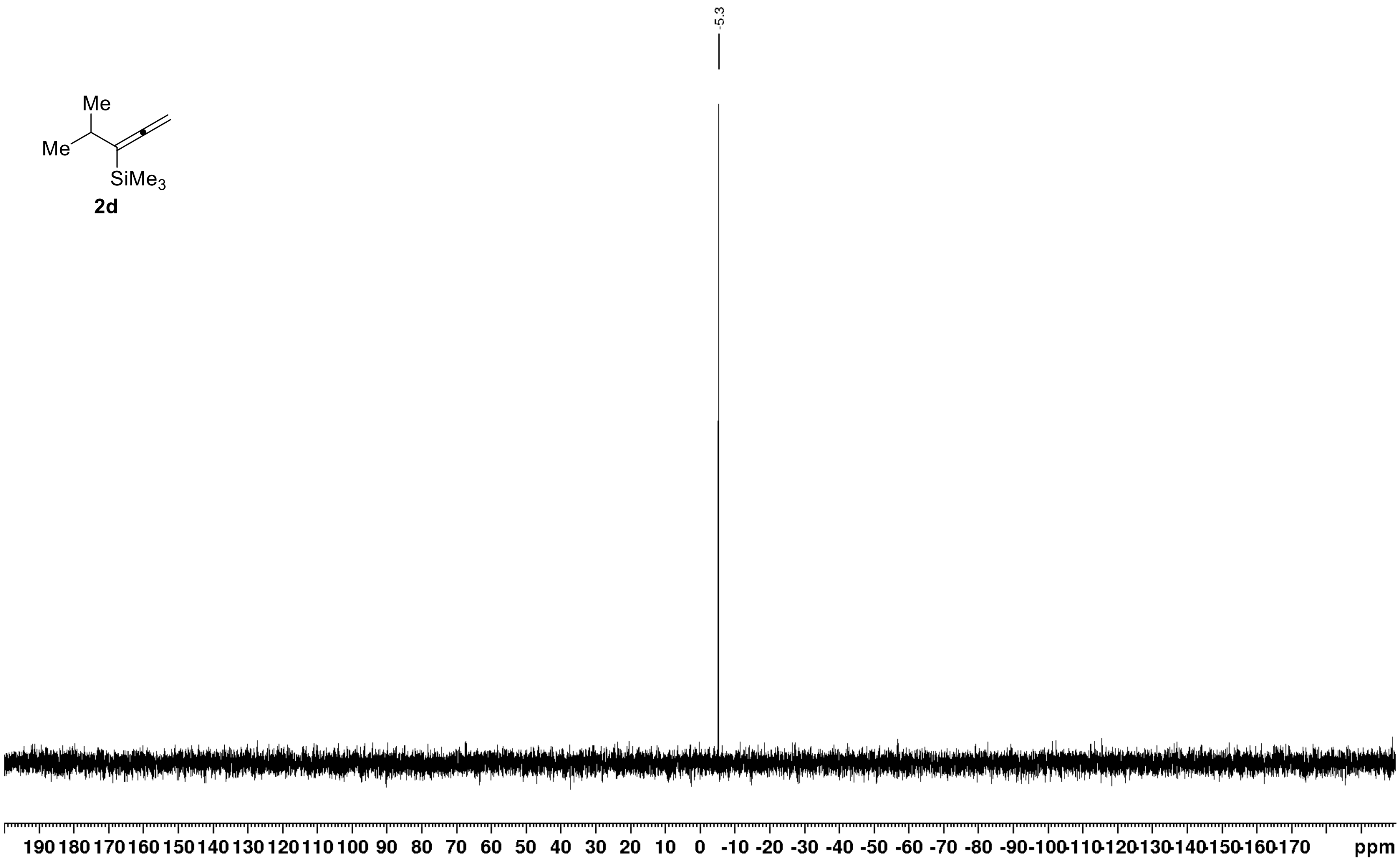

Figure S13. ^{1}H NMR (500 MHz, $CDCl_3$, 298 K) of **10aa**

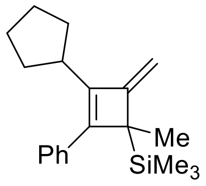

10aa

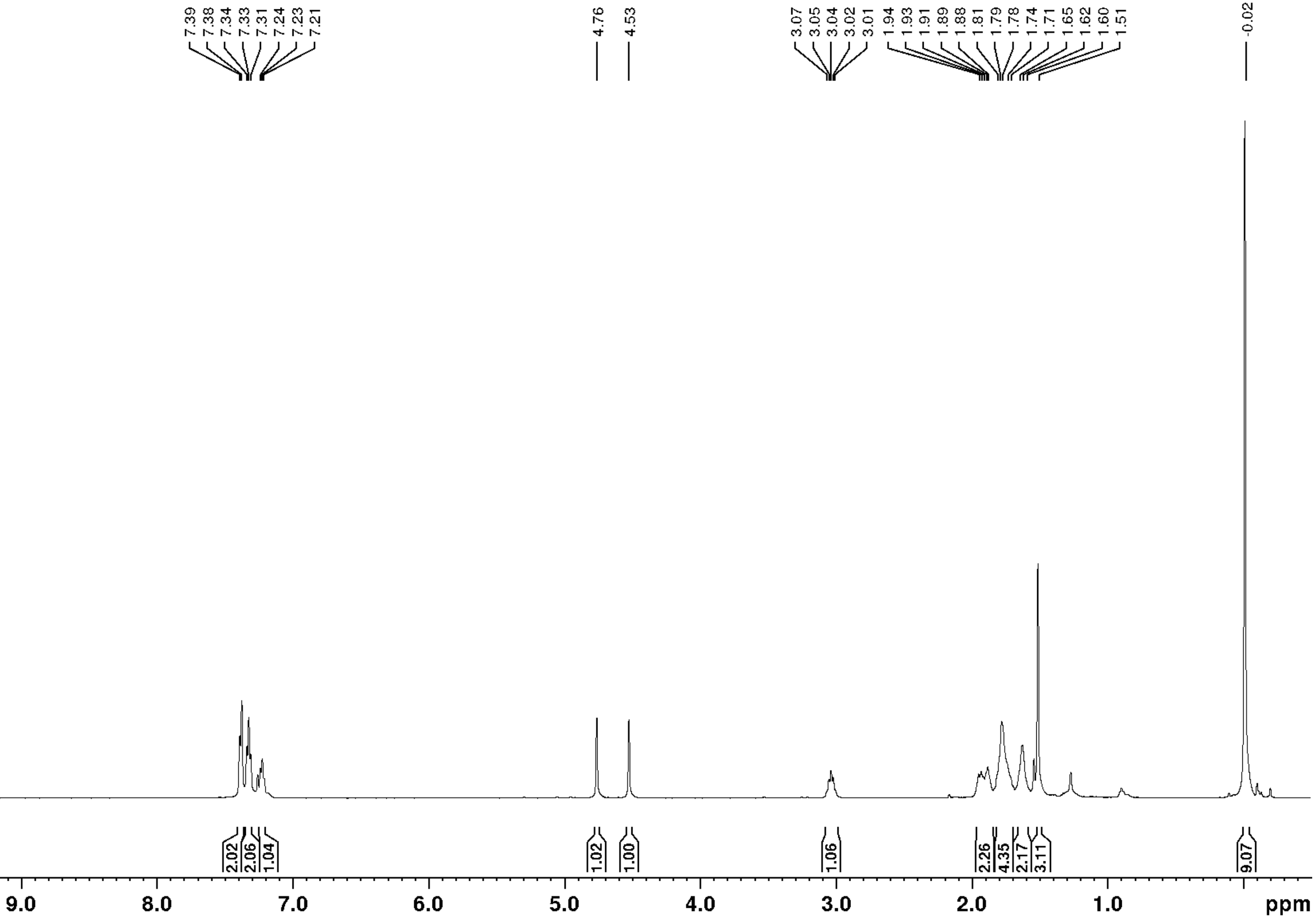

Figure S14. $^{13}C\{^{1}H\}$ NMR (126 MHz, $CDCl_3$, 298 K) of **10aa**

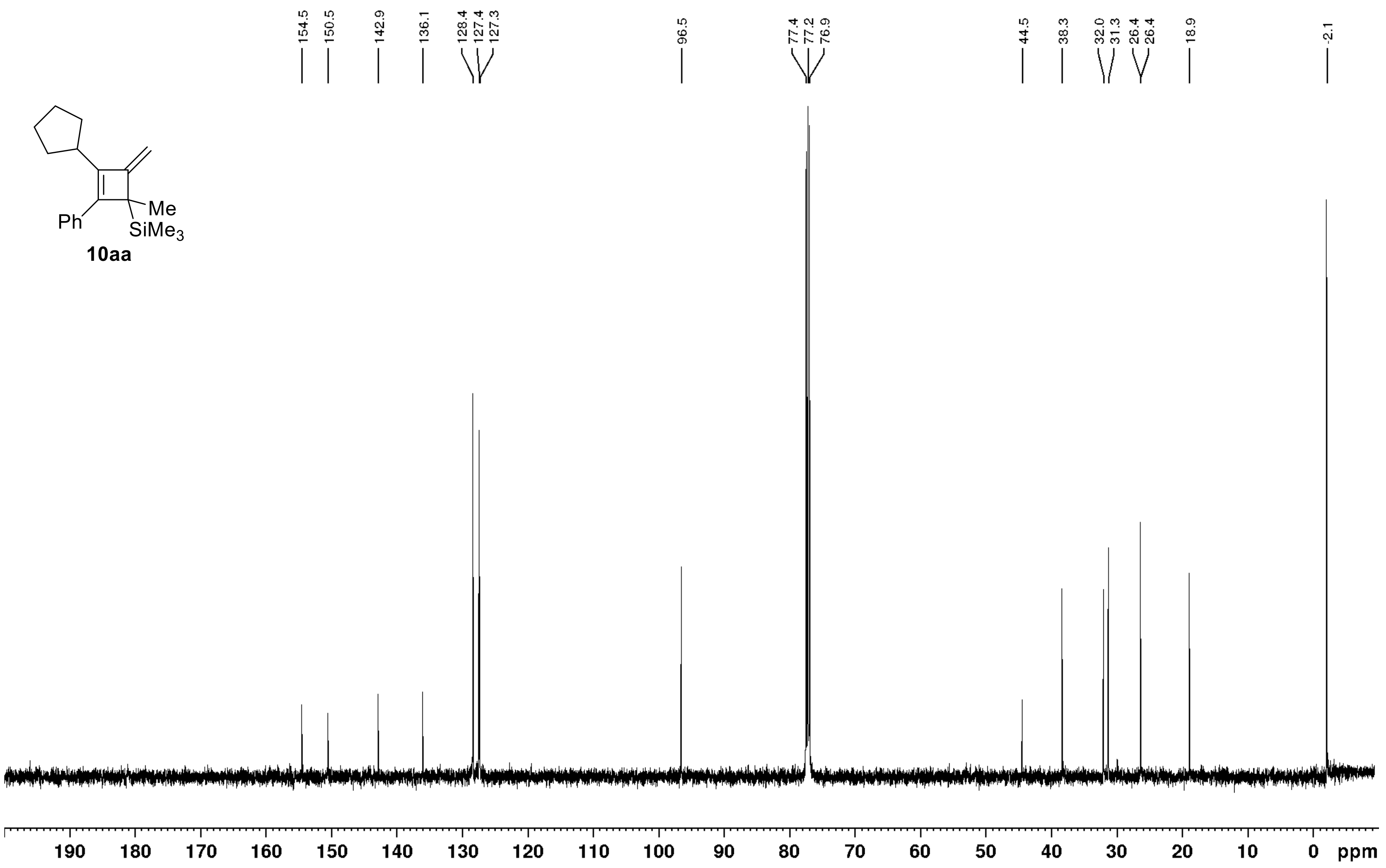

Figure S15. ^{29}Si DEPT NMR (99 MHz, $CDCl_3$, 298 K, optimized for J = 7.0 Hz) of **10aa**

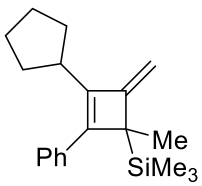

10aa

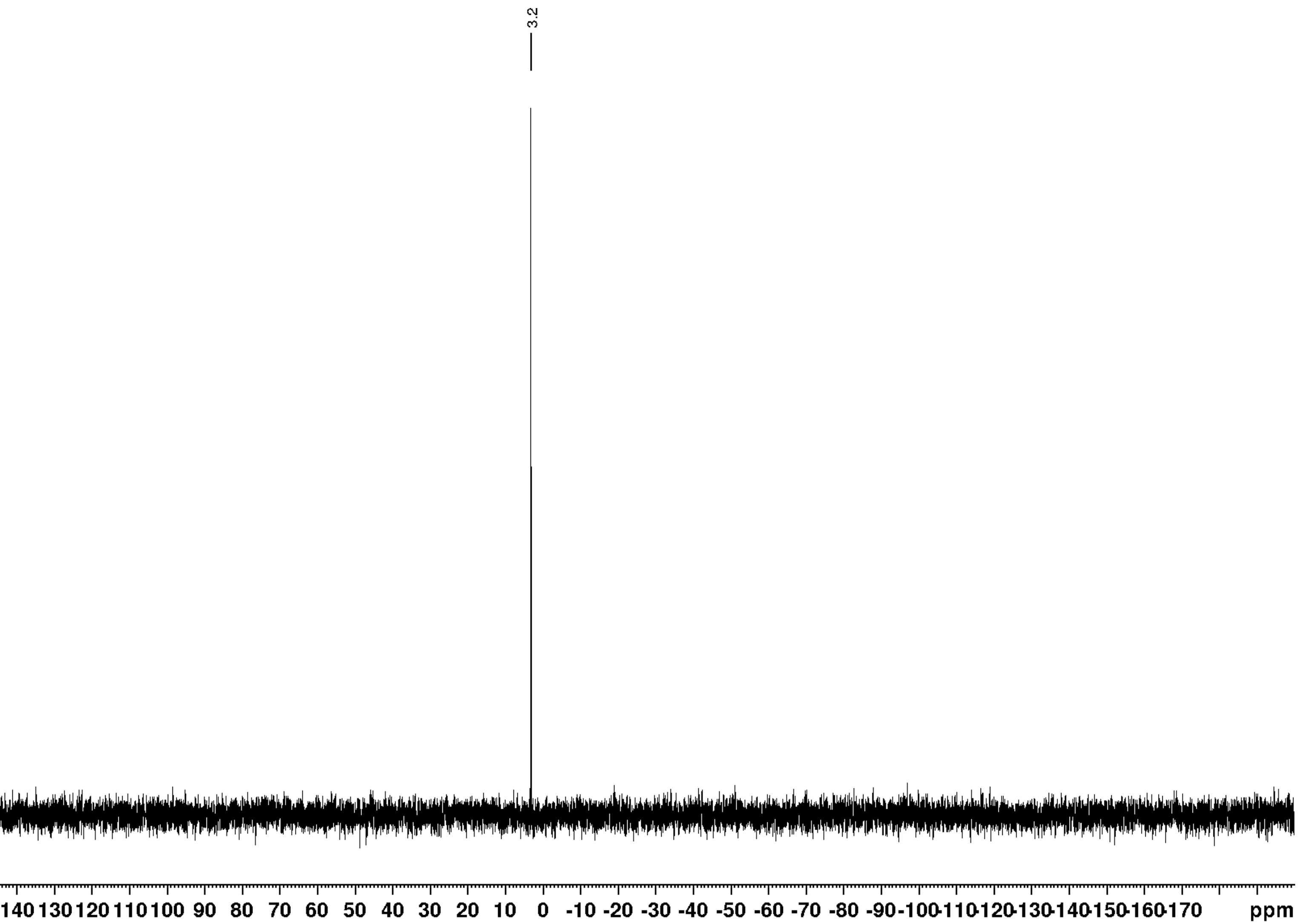

Figure S16. ^{1}H NMR (500 MHz, $CDCl_3$, 298 K) of **10ba**

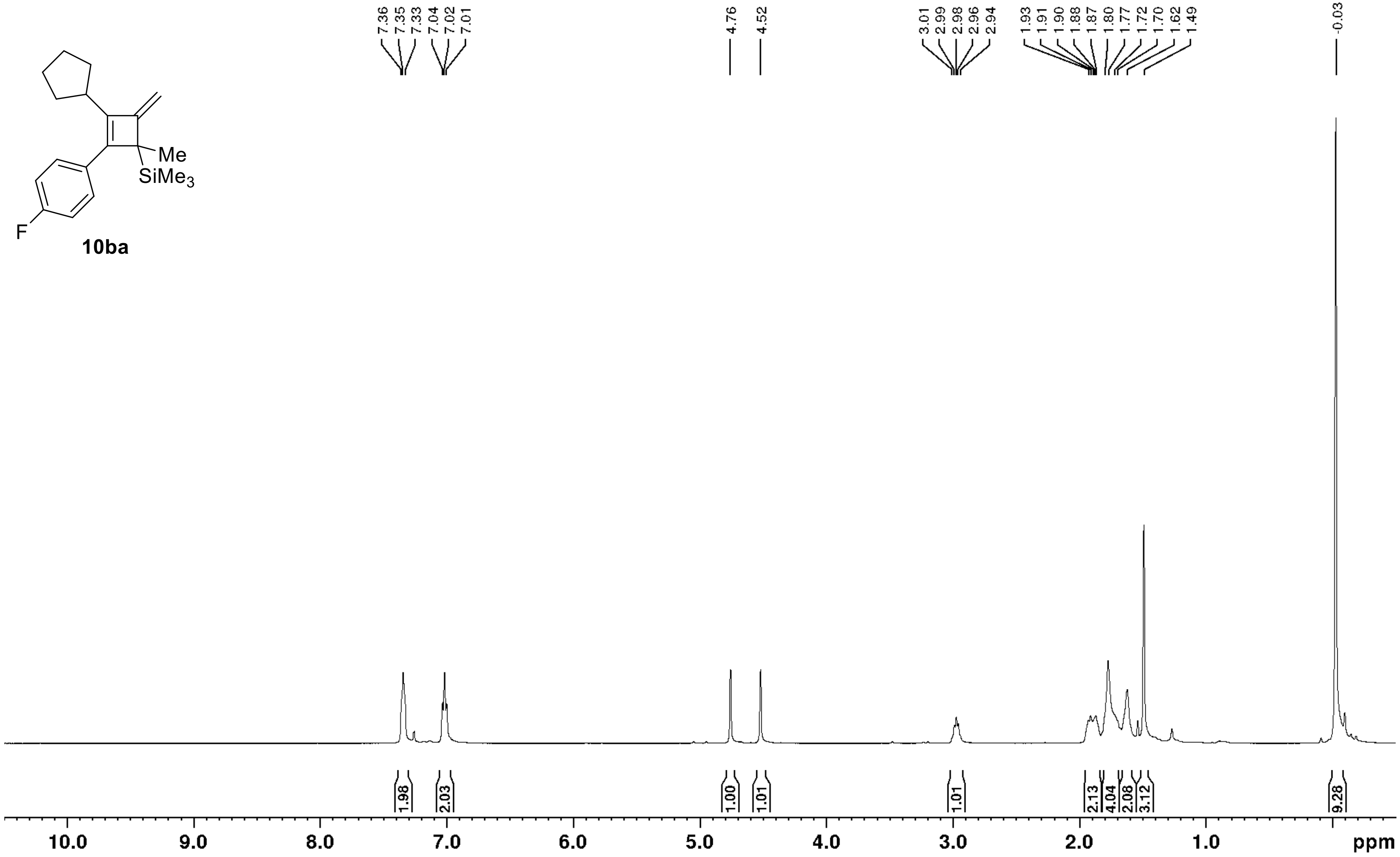

Figure S17. $^{13}C\{^{1}H\}$ NMR (126 MHz, $CDCl_3$, 298 K) of **10ba**

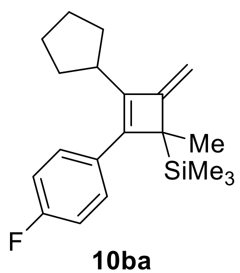

10ba

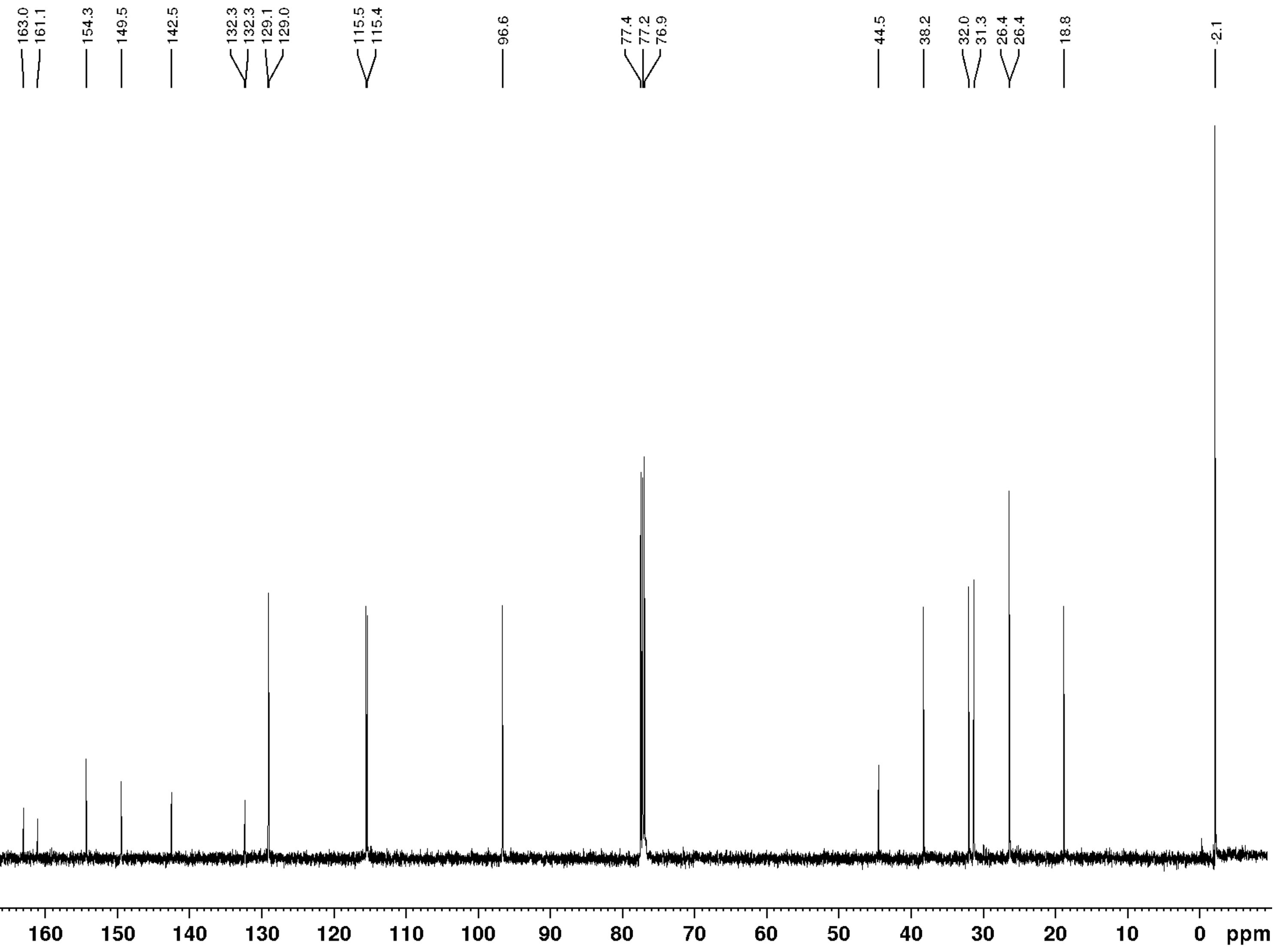

Figure S18. ^{19}F NMR (471 MHz, $CDCl_3$, 298 K) of **10ba**

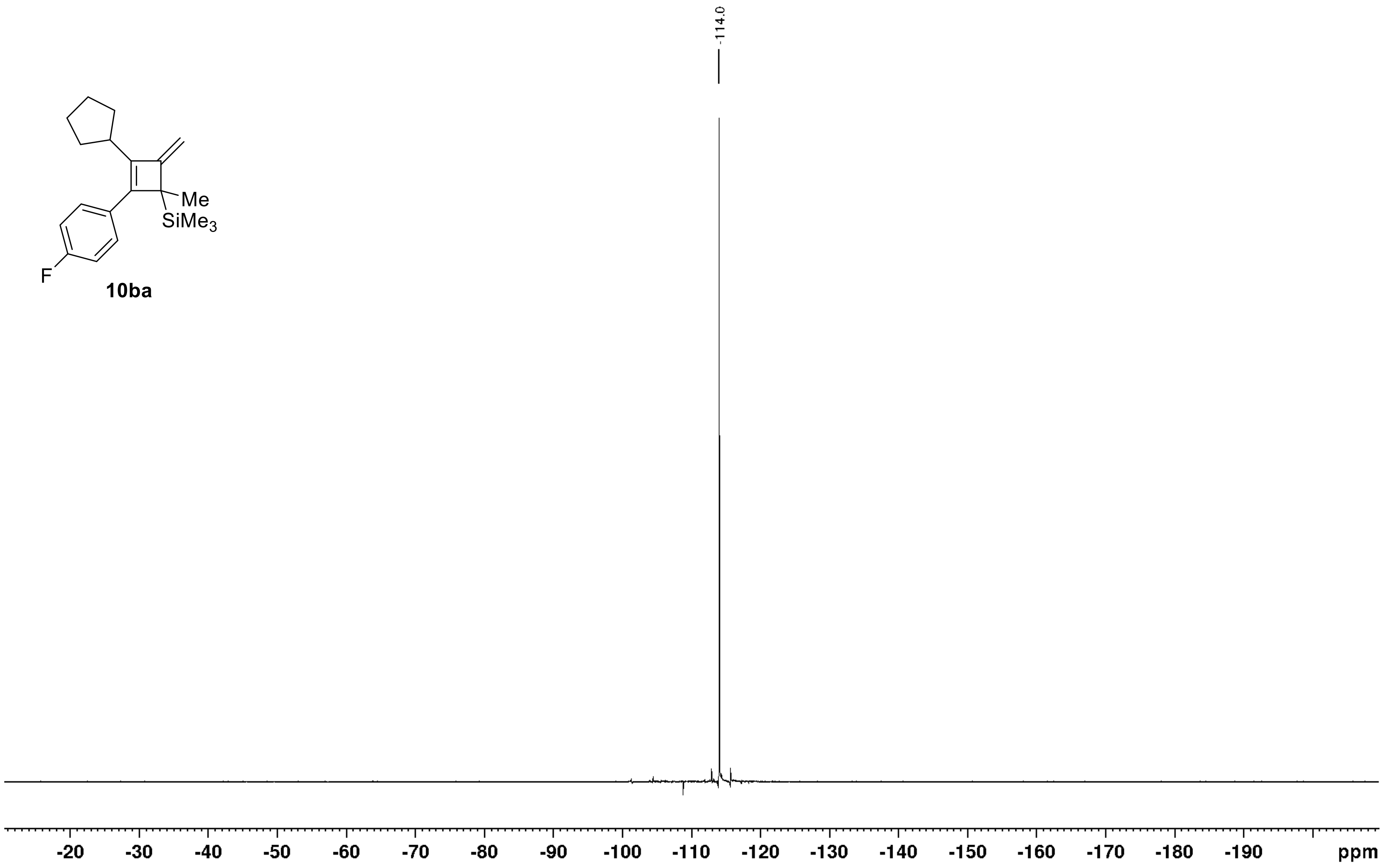

Figure S19. ^{29}Si DEPT NMR (99 MHz, $CDCl_3$, 298 K, optimized for J = 7.0 Hz) of **10ba**

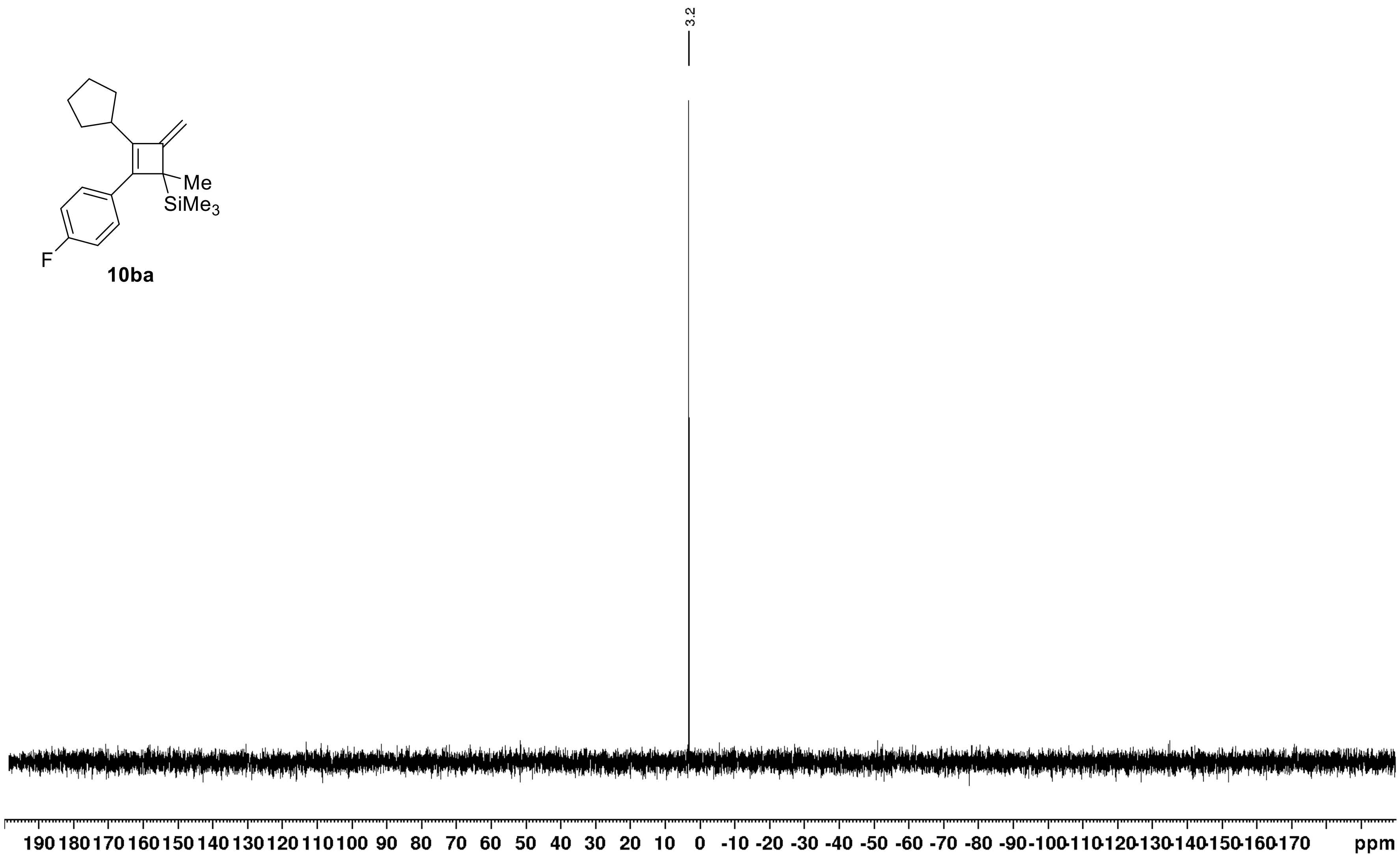

Figure S20. ^{1}H NMR (500 MHz, $CDCl_3$, 298 K) of **10ca**

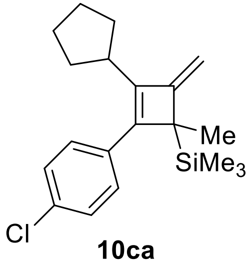

10ca

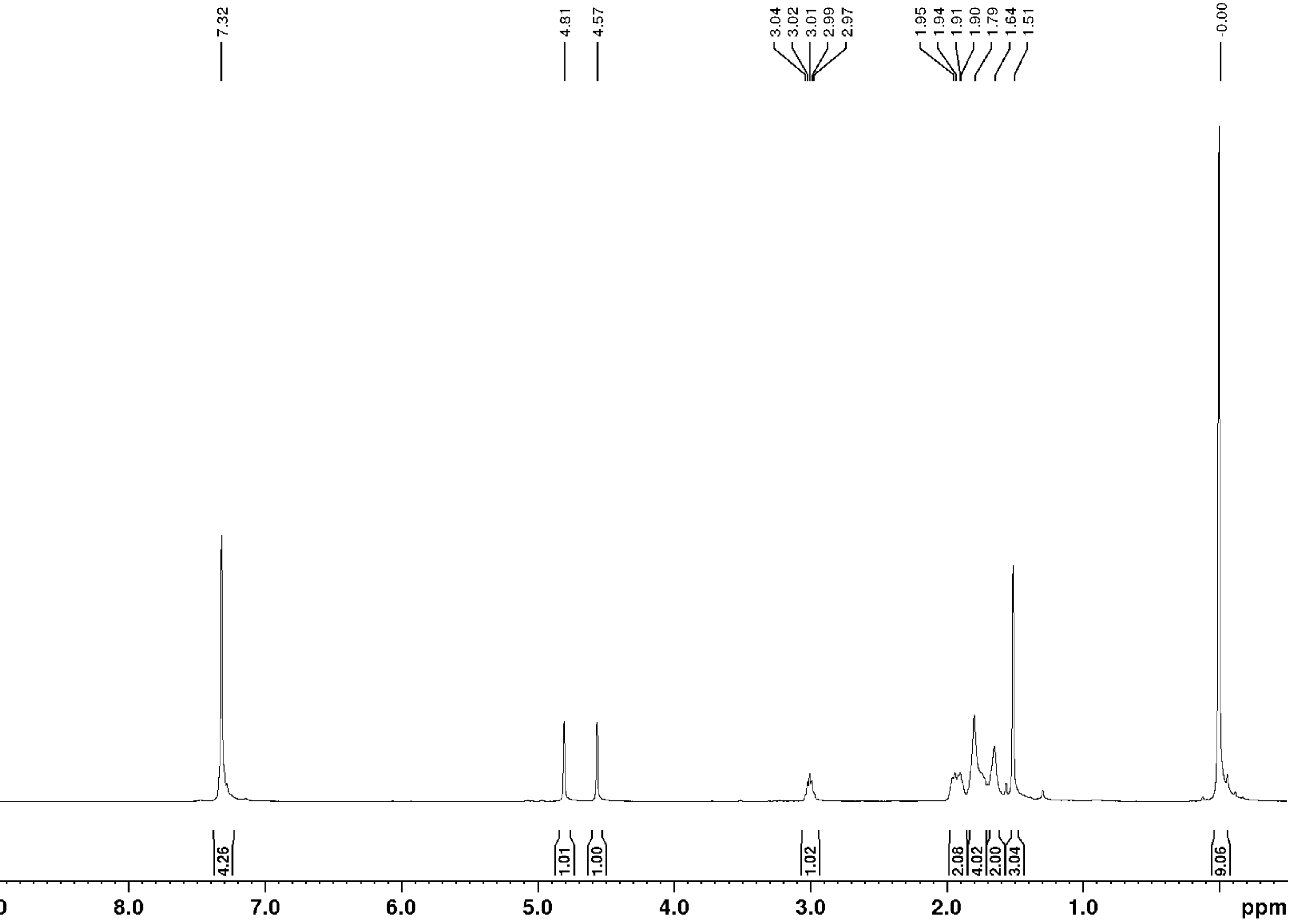

Figure S21. $^{13}C\{^{1}H\}$ NMR (126 MHz, $CDCl_3$, 298 K) of **10ca**

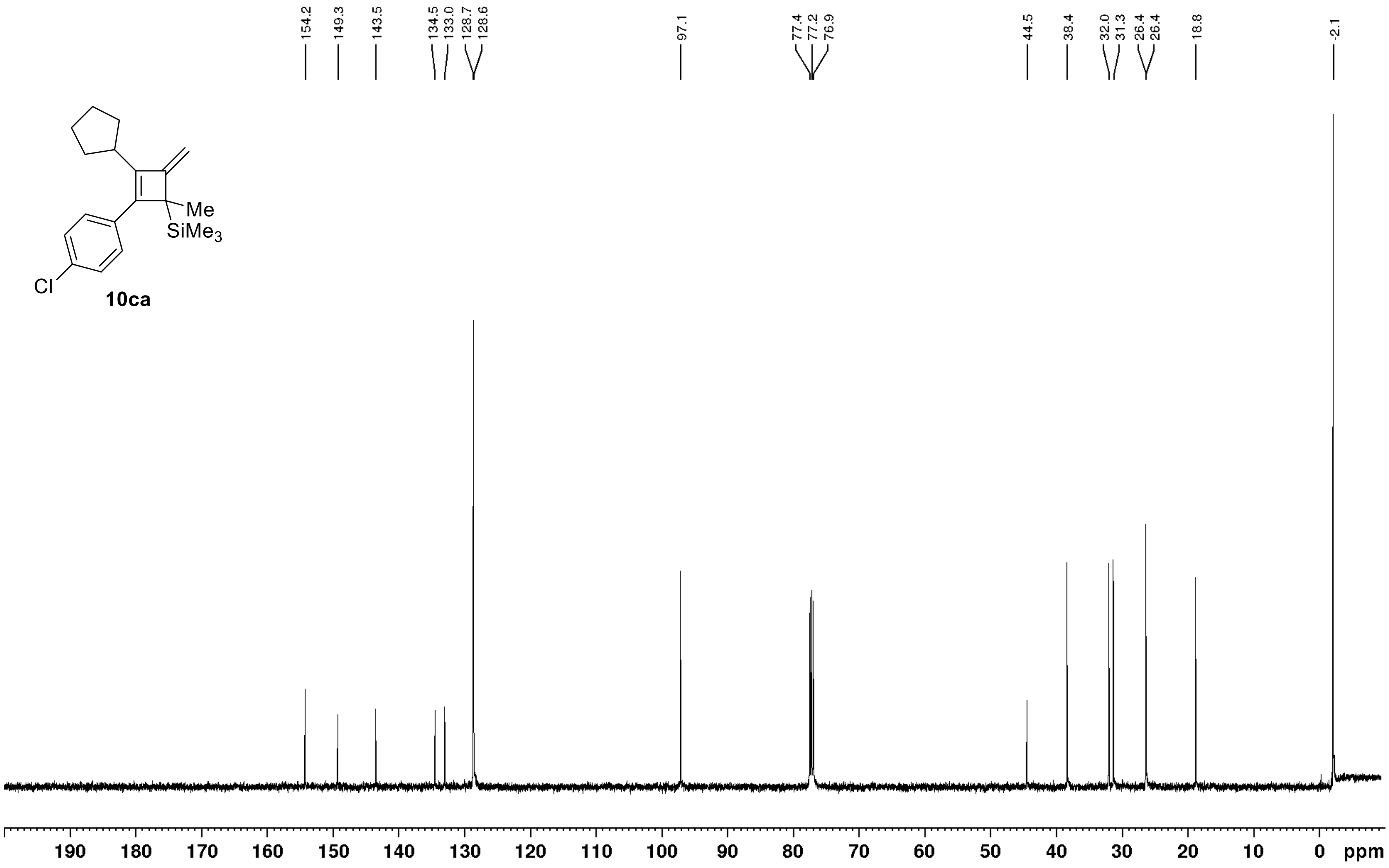

Figure S22. ^{29}Si DEPT NMR (99 MHz, $CDCl_3$, 298 K, optimized for J = 7.0 Hz) of **10ca**

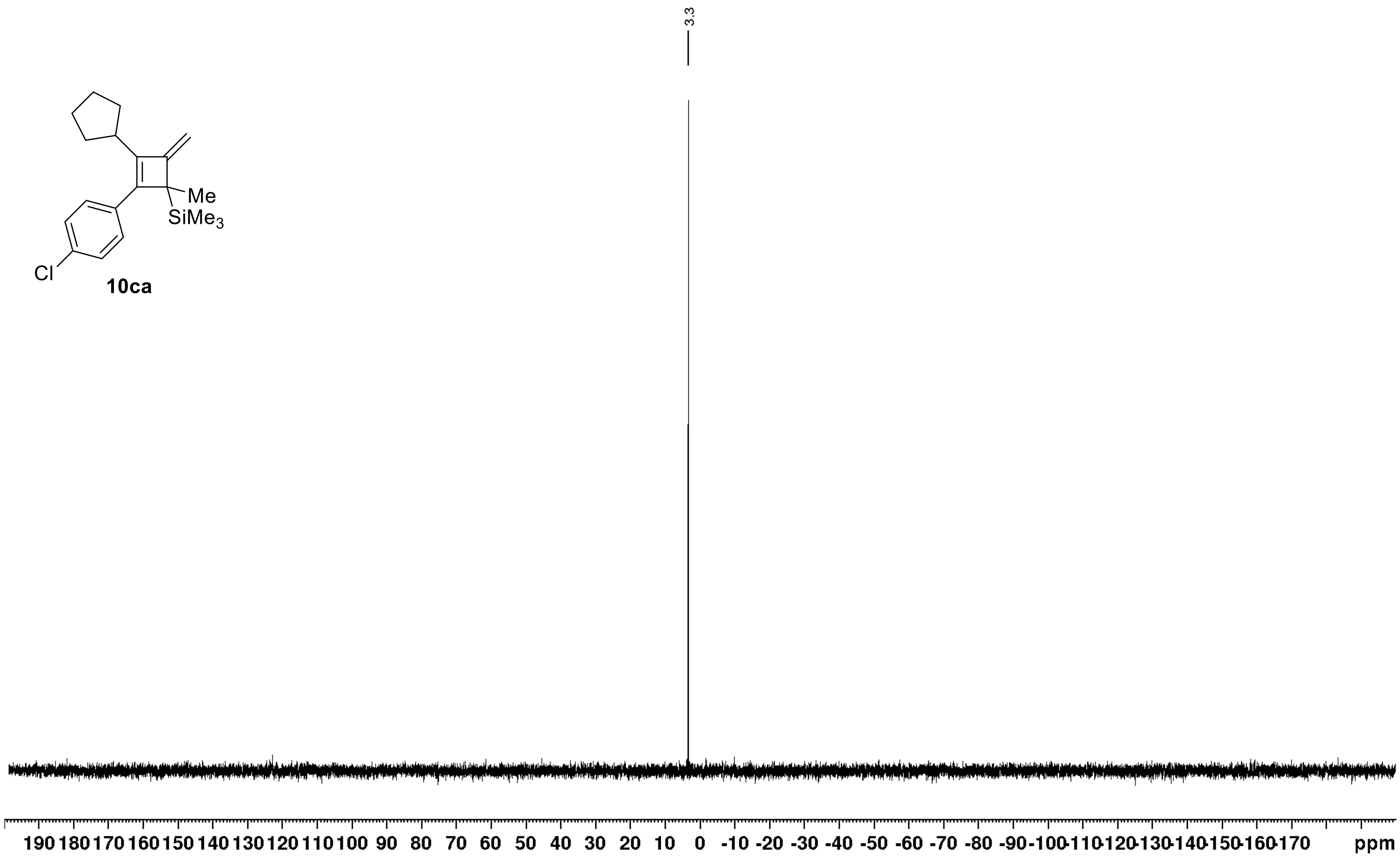

10ca

Figure S23. ^{1}H NMR (500 MHz, $CDCl_3$, 298 K) of **10da**

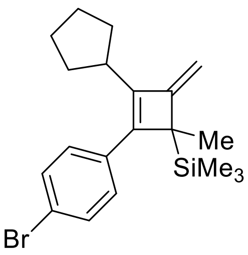

10da

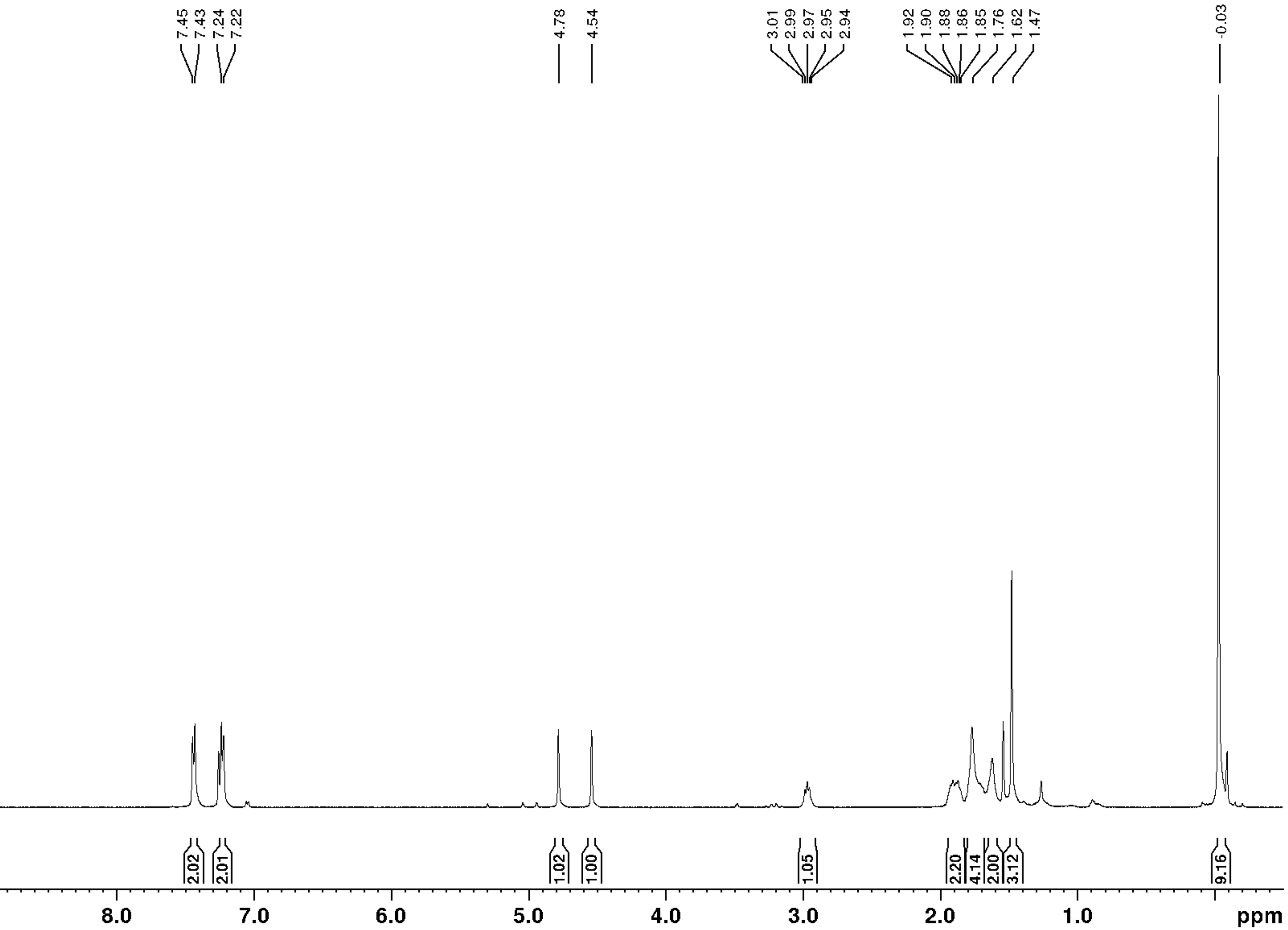

Figure S24. $^{13}C\{^{1}H\}$ NMR (126 MHz, $CDCl_3$, 298 K) of **10da**

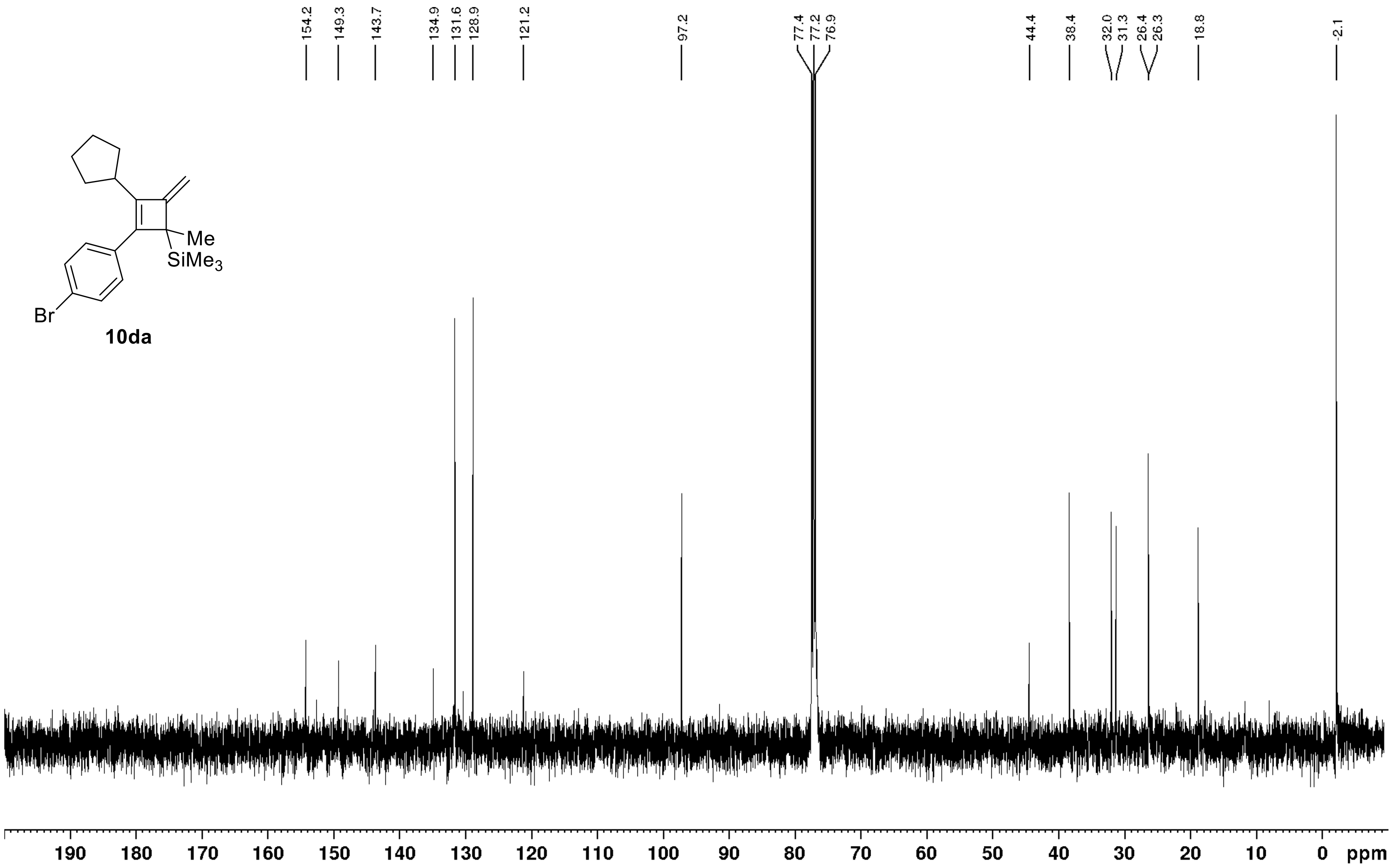

Figure S25. ^{29}Si DEPT NMR (99 MHz, $CDCl_3$, 298 K, optimized for J = 7.0 Hz) of **10da**

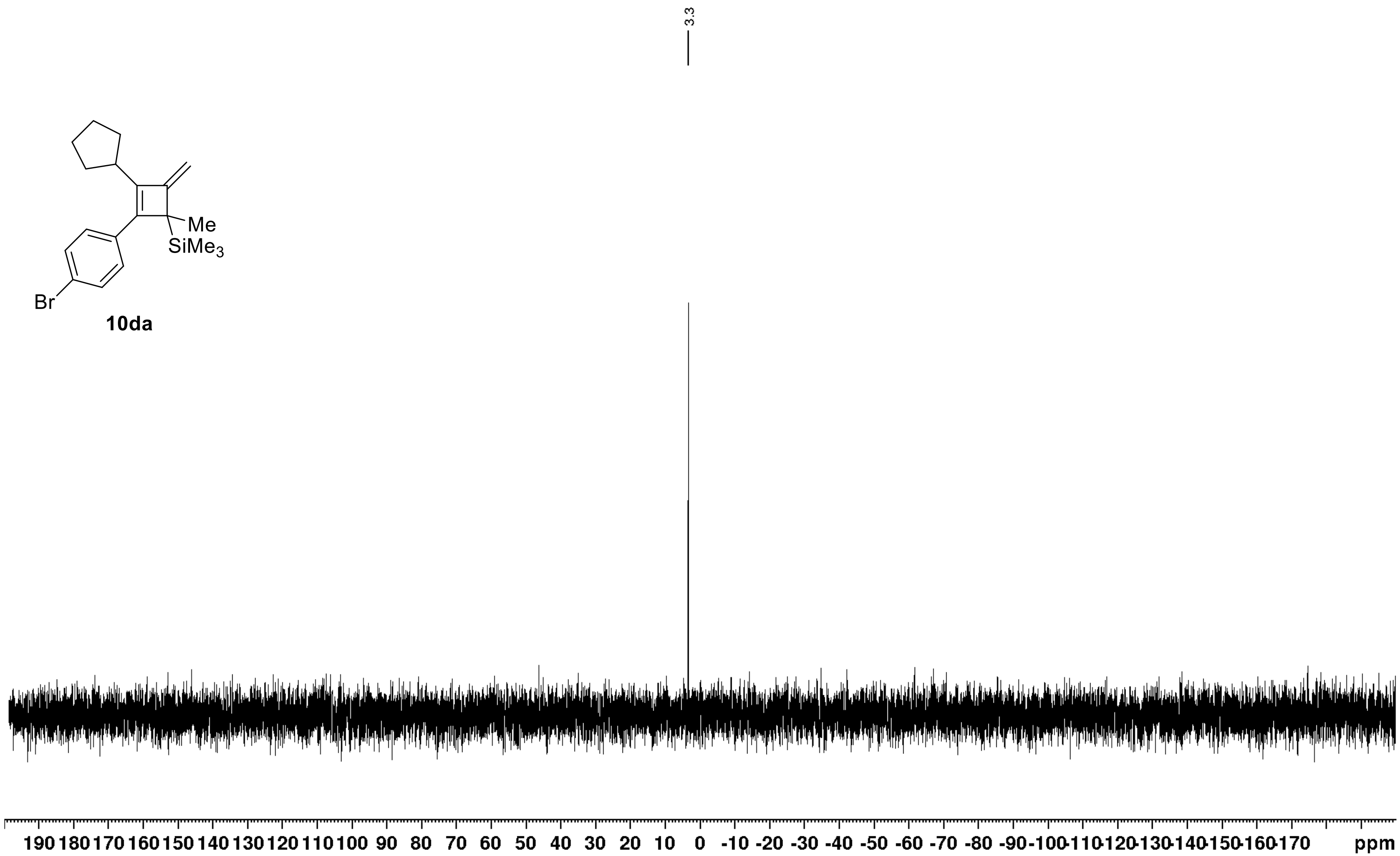

Figure S26. ^{1}H NMR (500 MHz, $CDCl_3$, 298 K) of **10fa**

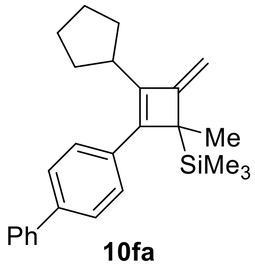

10fa

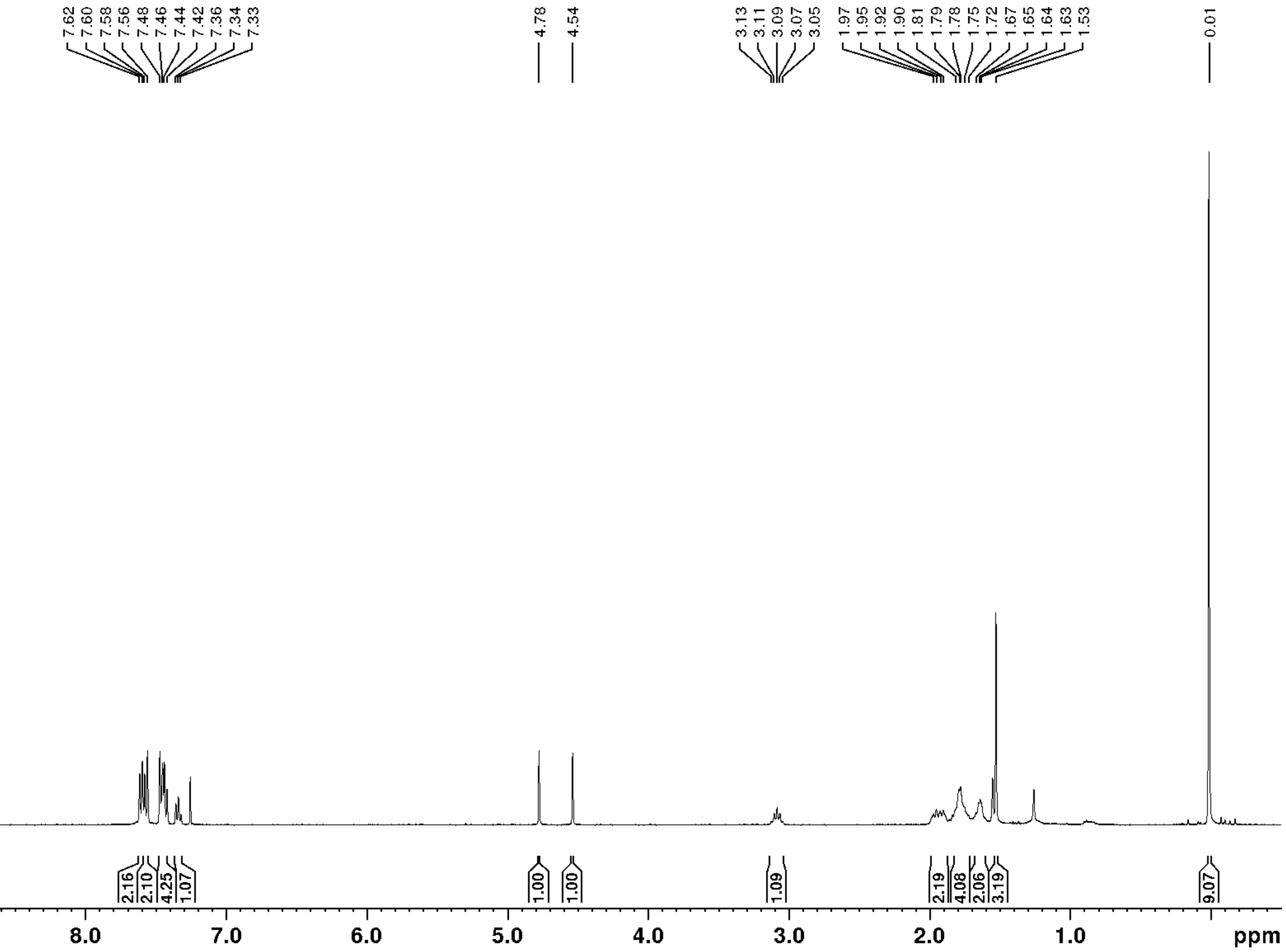

Figure S27. $^{13}C\{^{1}H\}$ NMR (126 MHz, $CDCl_3$, 298 K) of **10fa**

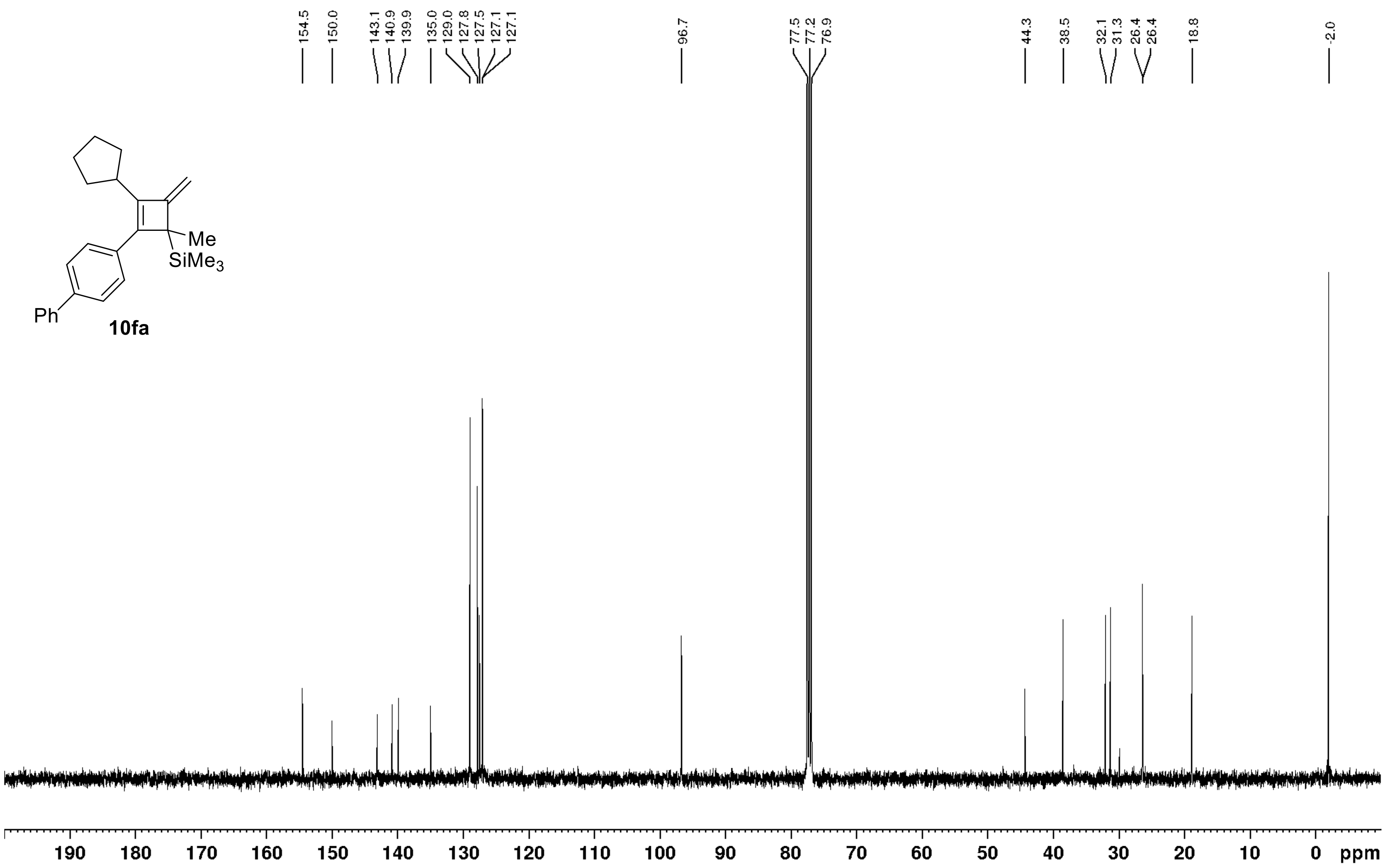

Figure S28. ^{29}Si DEPT NMR (99 MHz, $CDCl_3$, 298 K, optimized for J = 7.0 Hz) of **10fa**

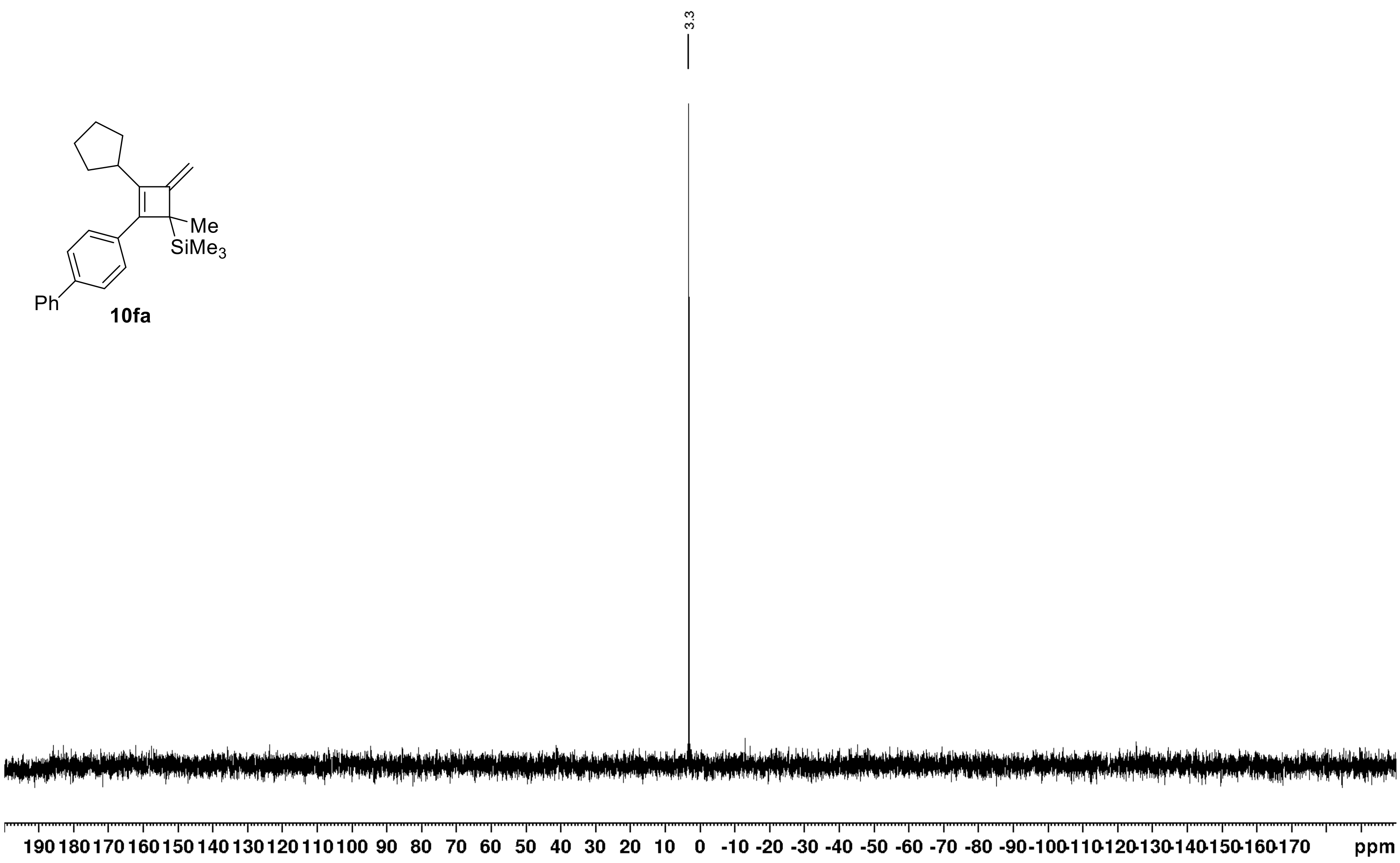

Figure S29. ^{1}H NMR (500 MHz, $CDCl_3$, 298 K) of **10ha**

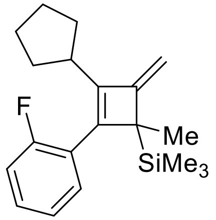

10ha

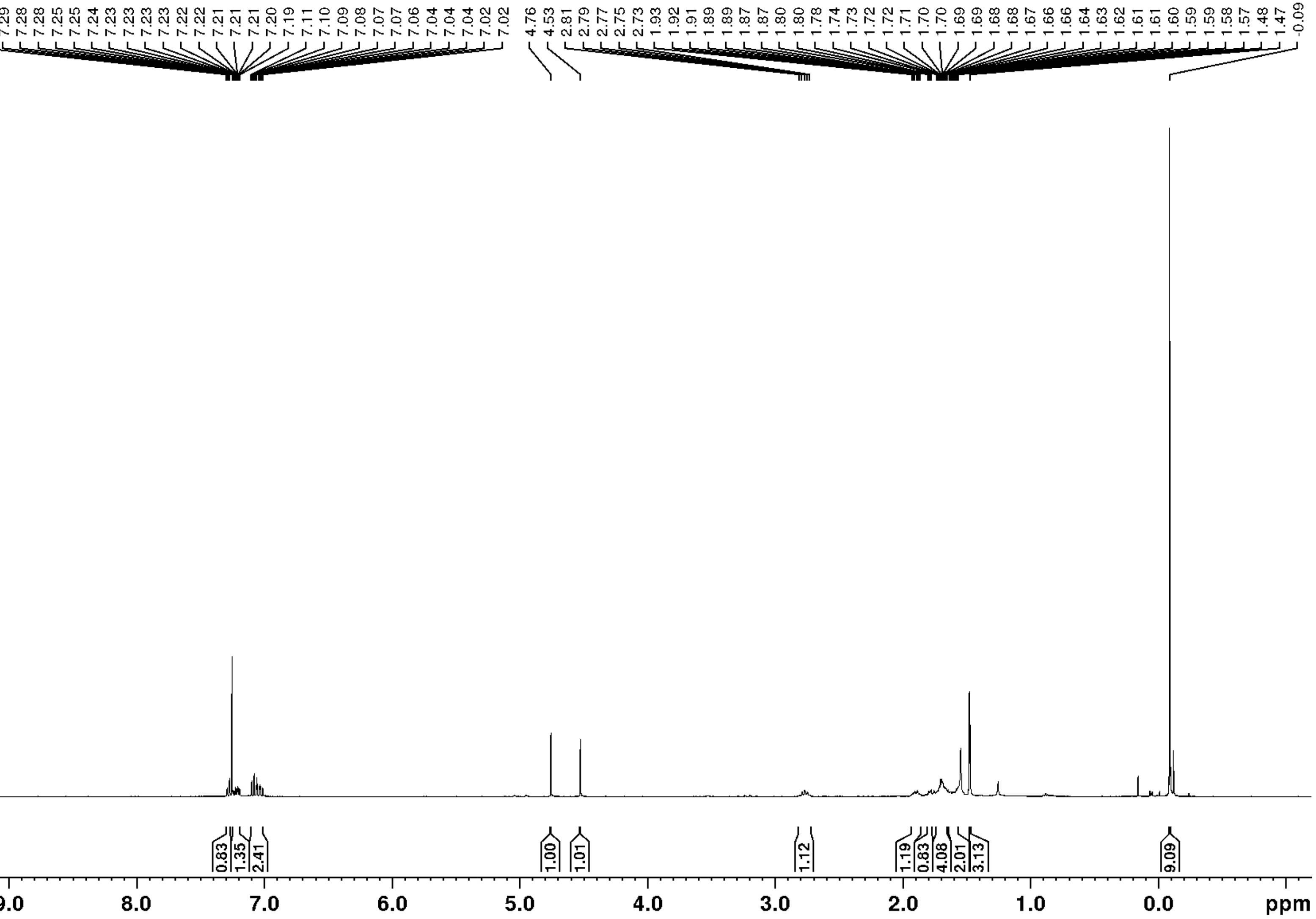

Figure S30. $^{13}C\{^{1}H\}$ NMR (126 MHz, $CDCl_3$, 298 K) of **10ha**

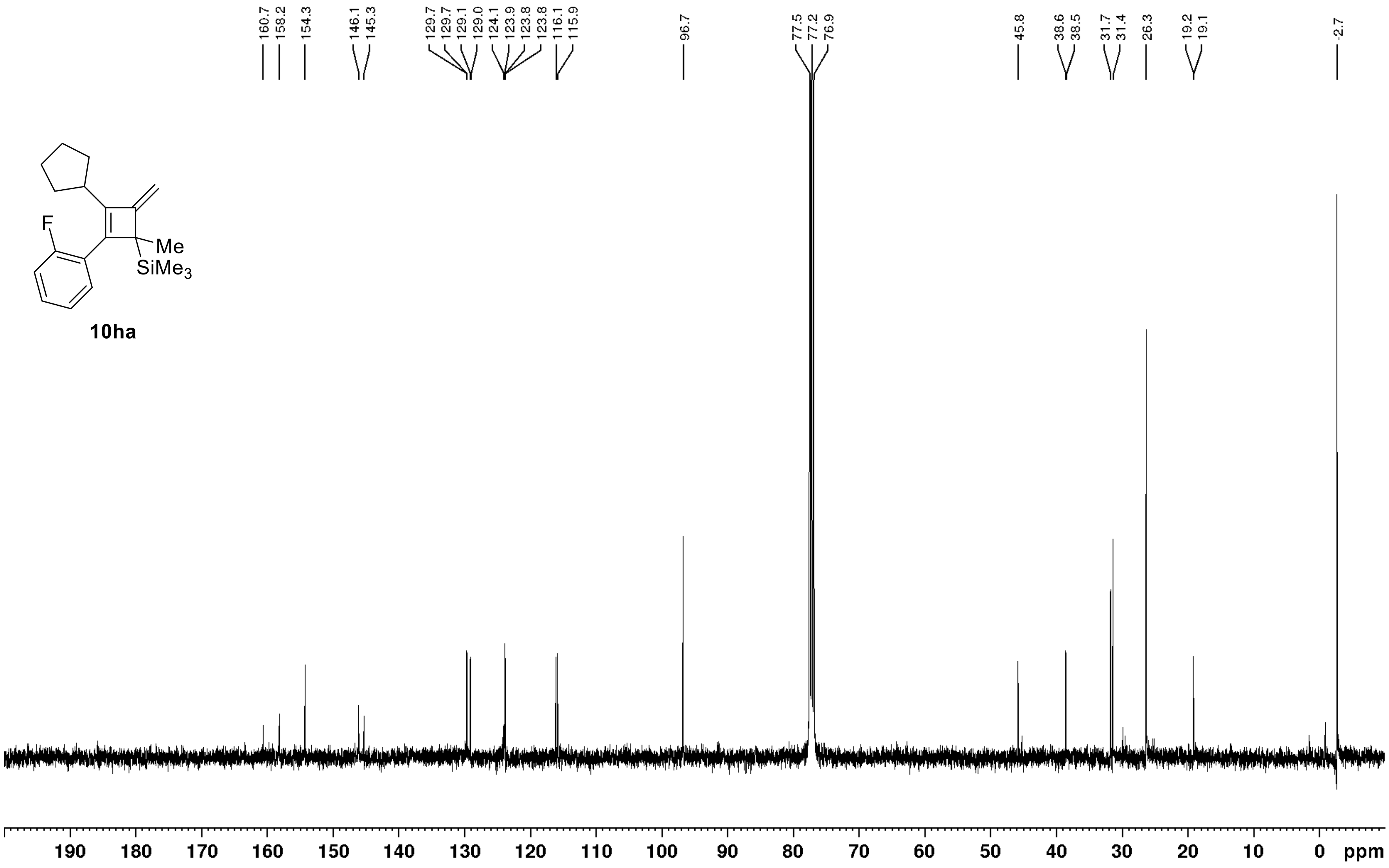

Figure S31. ^{19}F NMR (471 MHz, $CDCl_3$, 298 K) of **10ha**

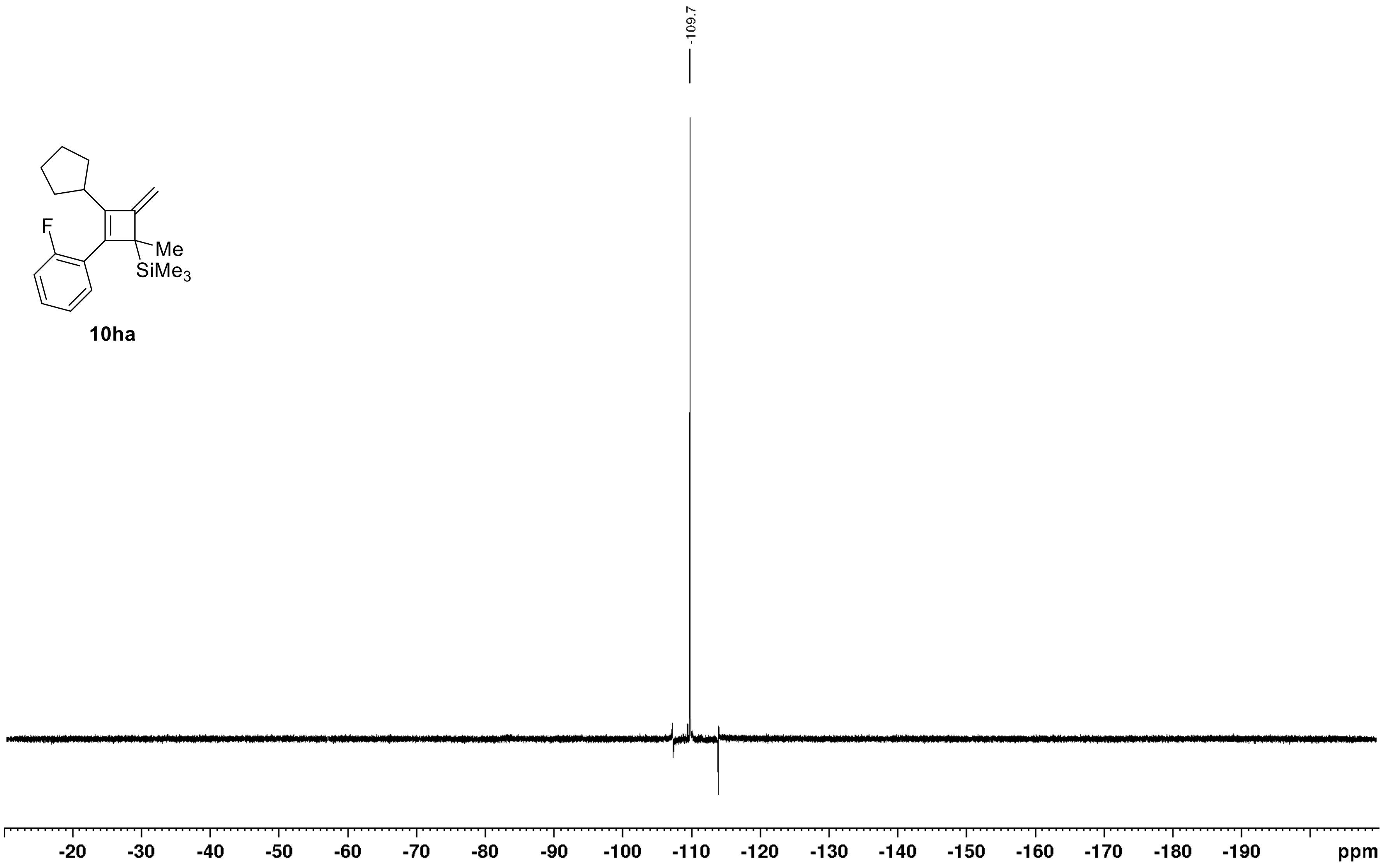

Figure S32. ^{29}Si DEPT NMR (99 MHz, $CDCl_3$, 298 K, optimized for J = 7.0 Hz) of **10ha**

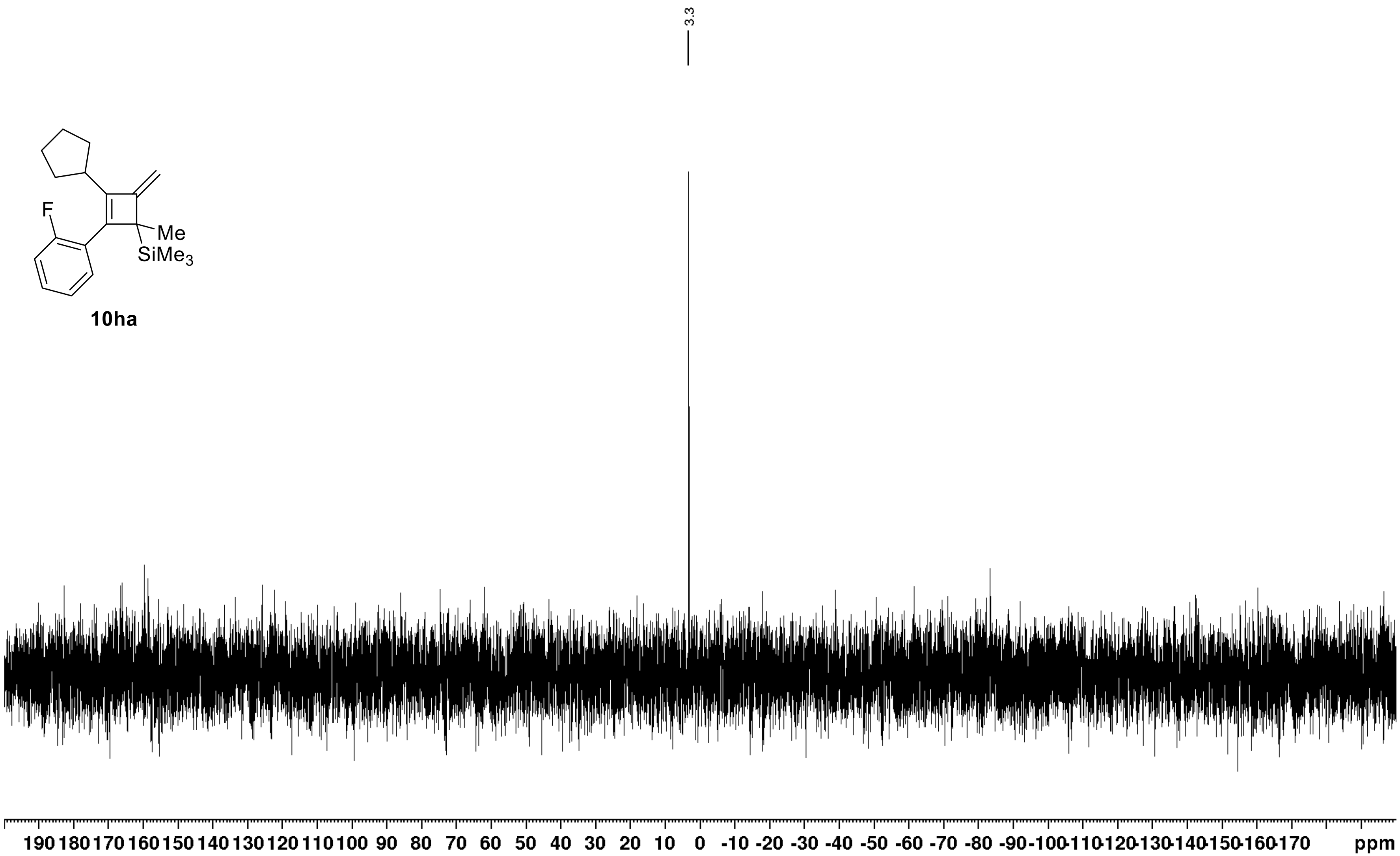

Figure S33. ^{1}H NMR (500 MHz, $CDCl_3$, 298 K) of **10ia**

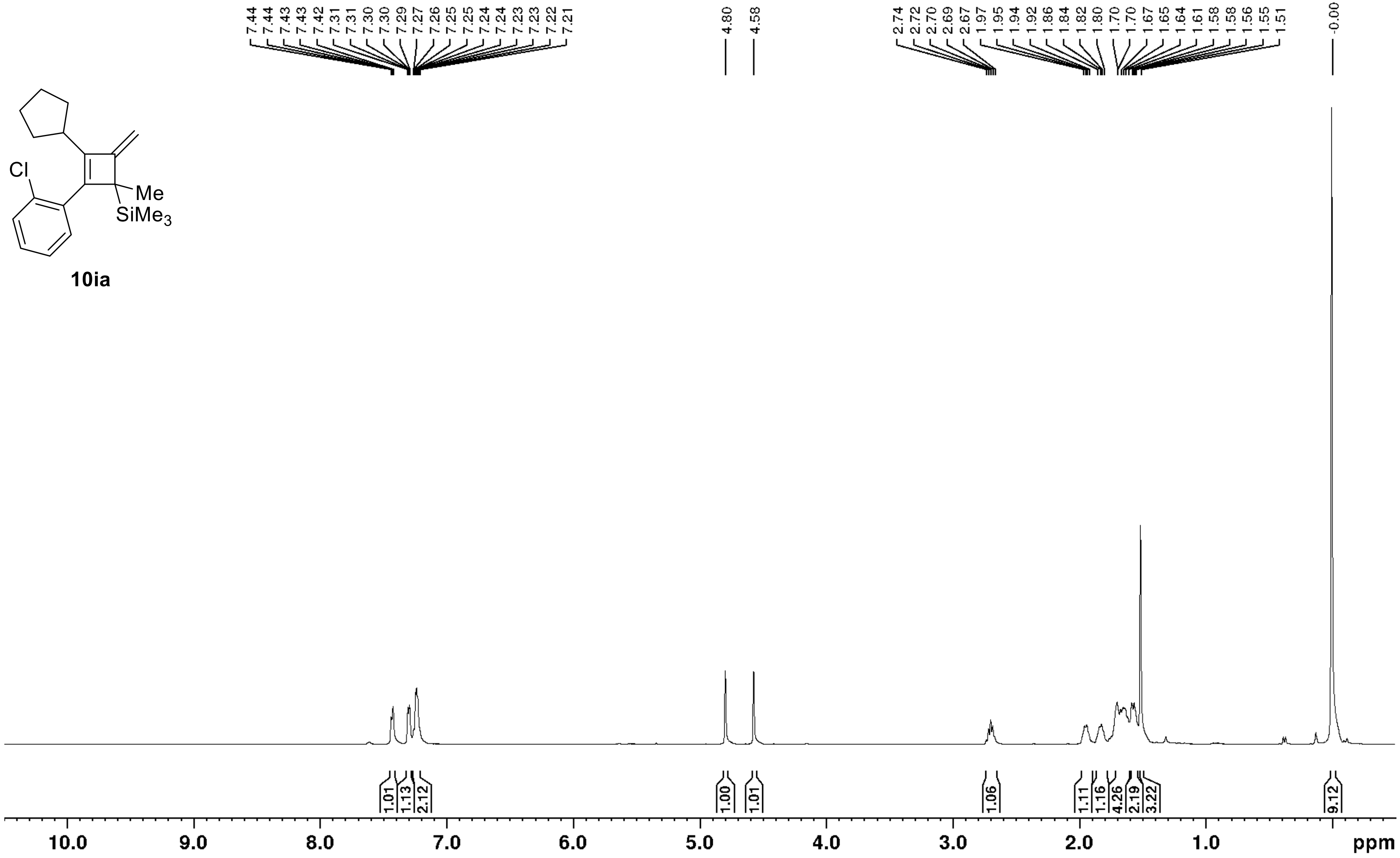

Figure S34. $^{13}C\{^1H\}$ NMR (126 MHz, $CDCl_3$, 298 K) of **10ia**

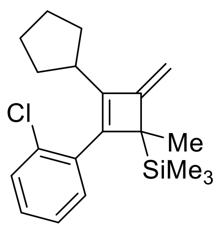

10ia

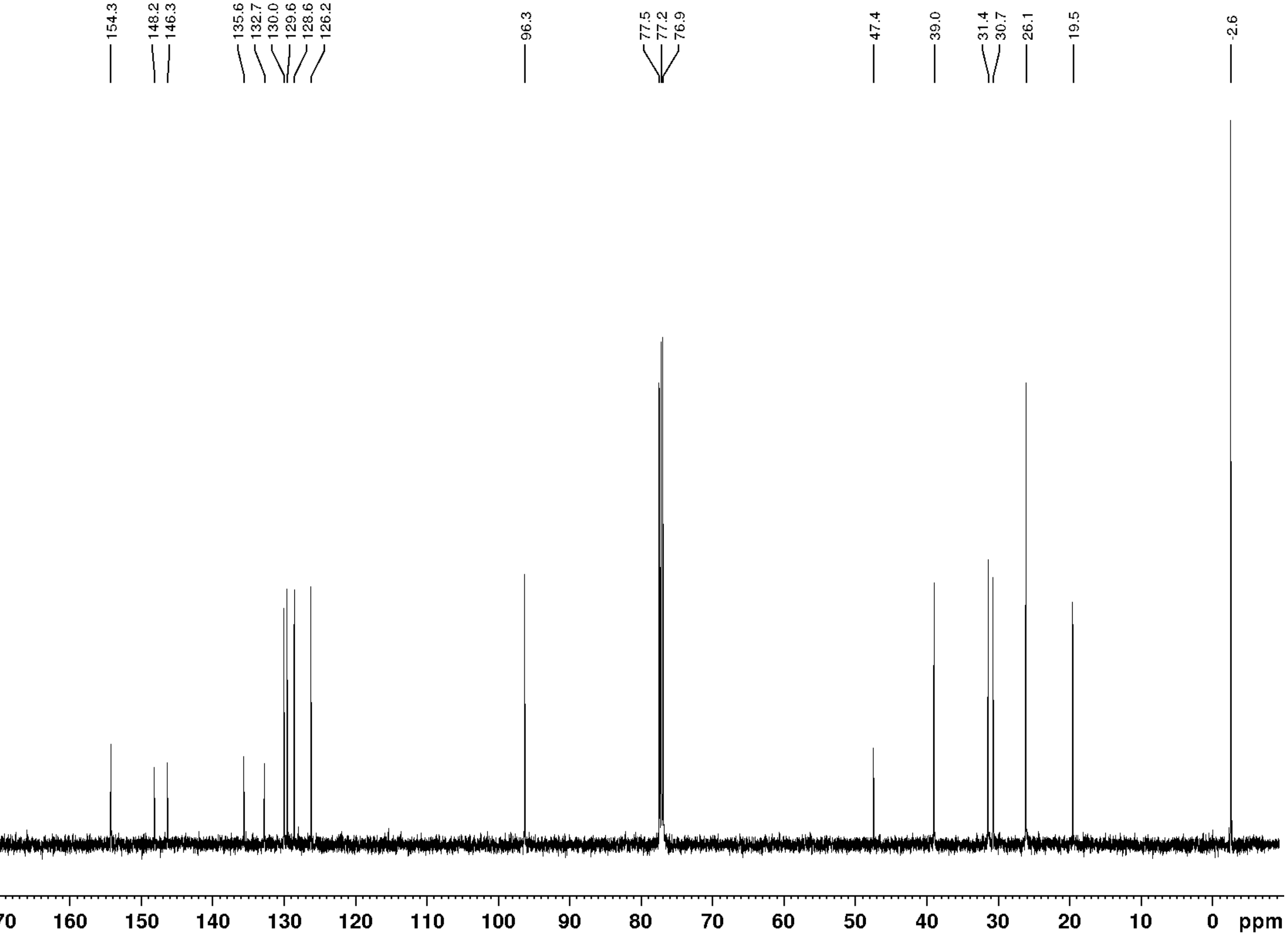

Figure S35. ^{29}Si DEPT NMR (99 MHz, $CDCl_3$, 298 K, optimized for J = 7.0 Hz) of **10ia**

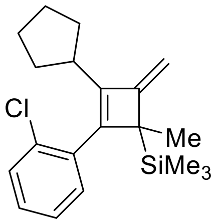

10ia

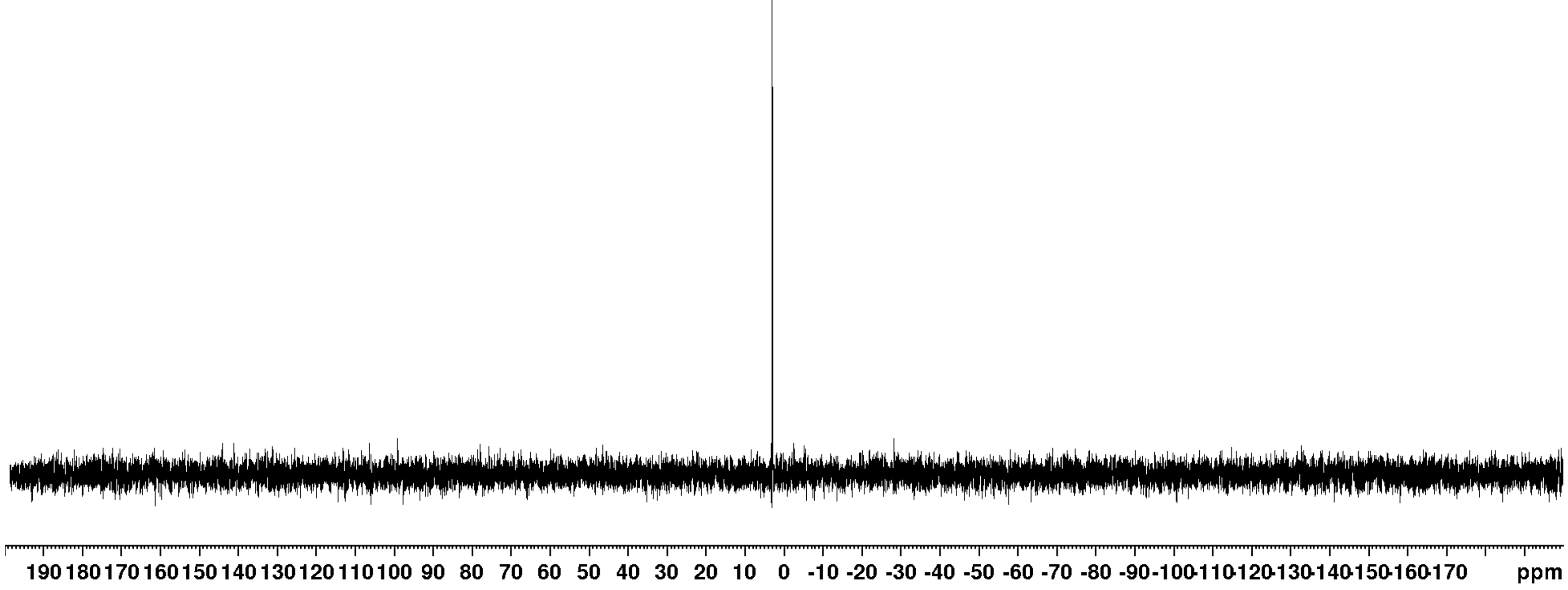

Figure S36. ^{1}H NMR (500 MHz, $CDCl_3$, 298 K) of **10ja**

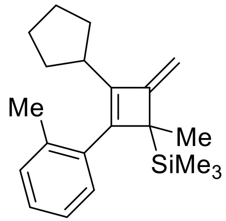

10ja

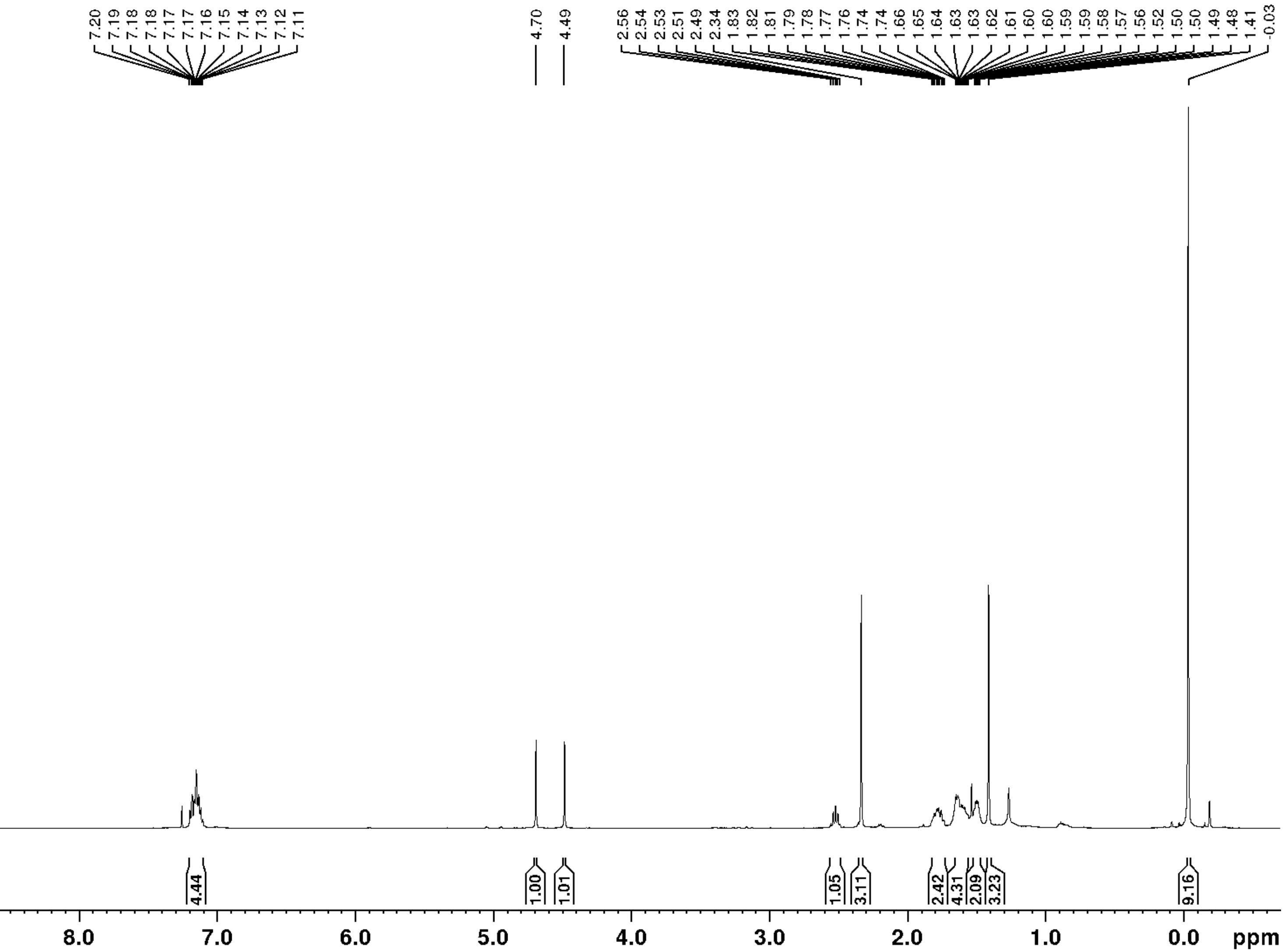

Figure S37. $^{13}C\{^{1}H\}$ NMR (126 MHz, $CDCl_3$, 298 K) of **10ja**

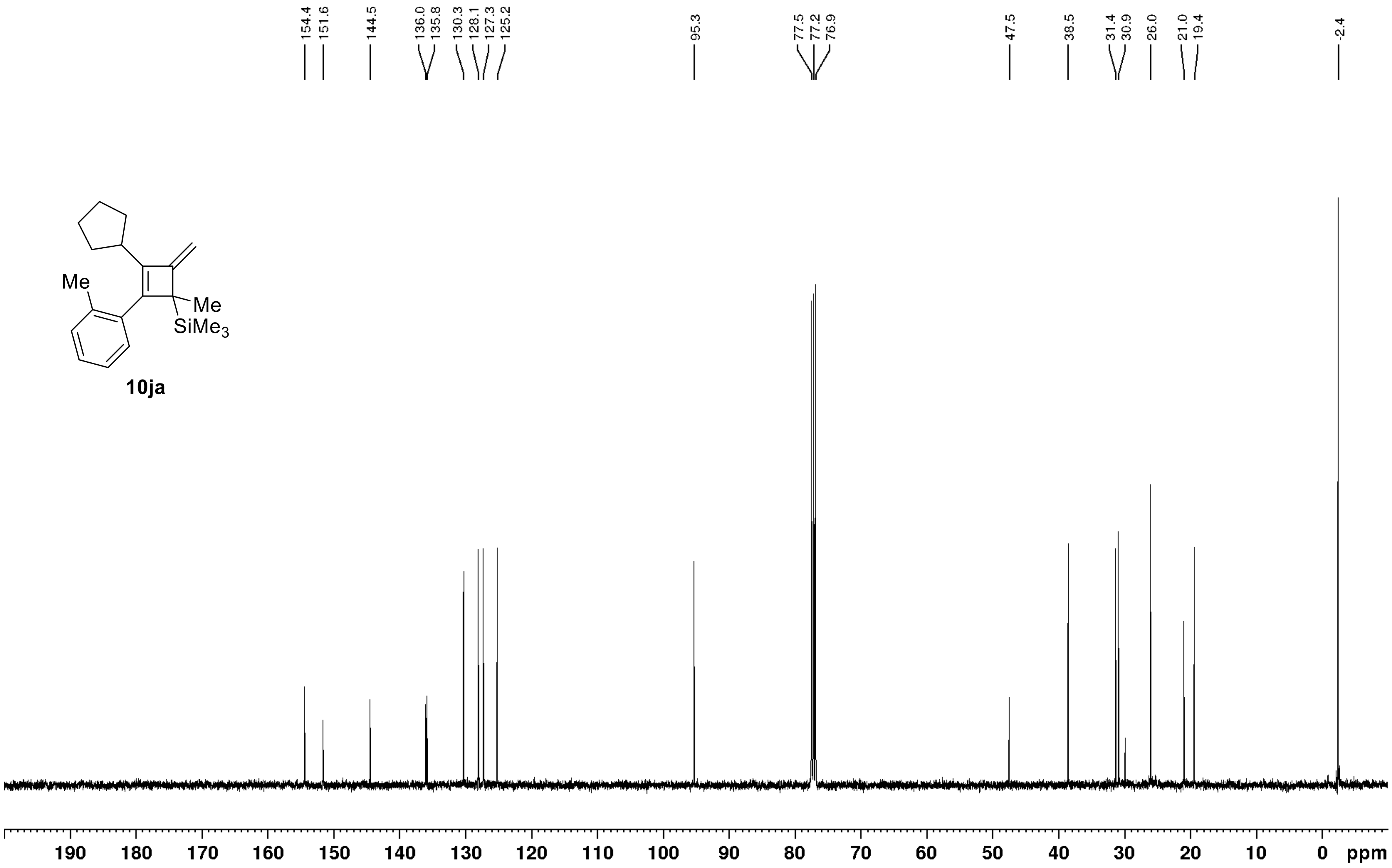

Figure S38. ^{29}Si DEPT NMR (99 MHz, $CDCl_3$, 298 K, optimized for J = 7.0 Hz) of **10ja**

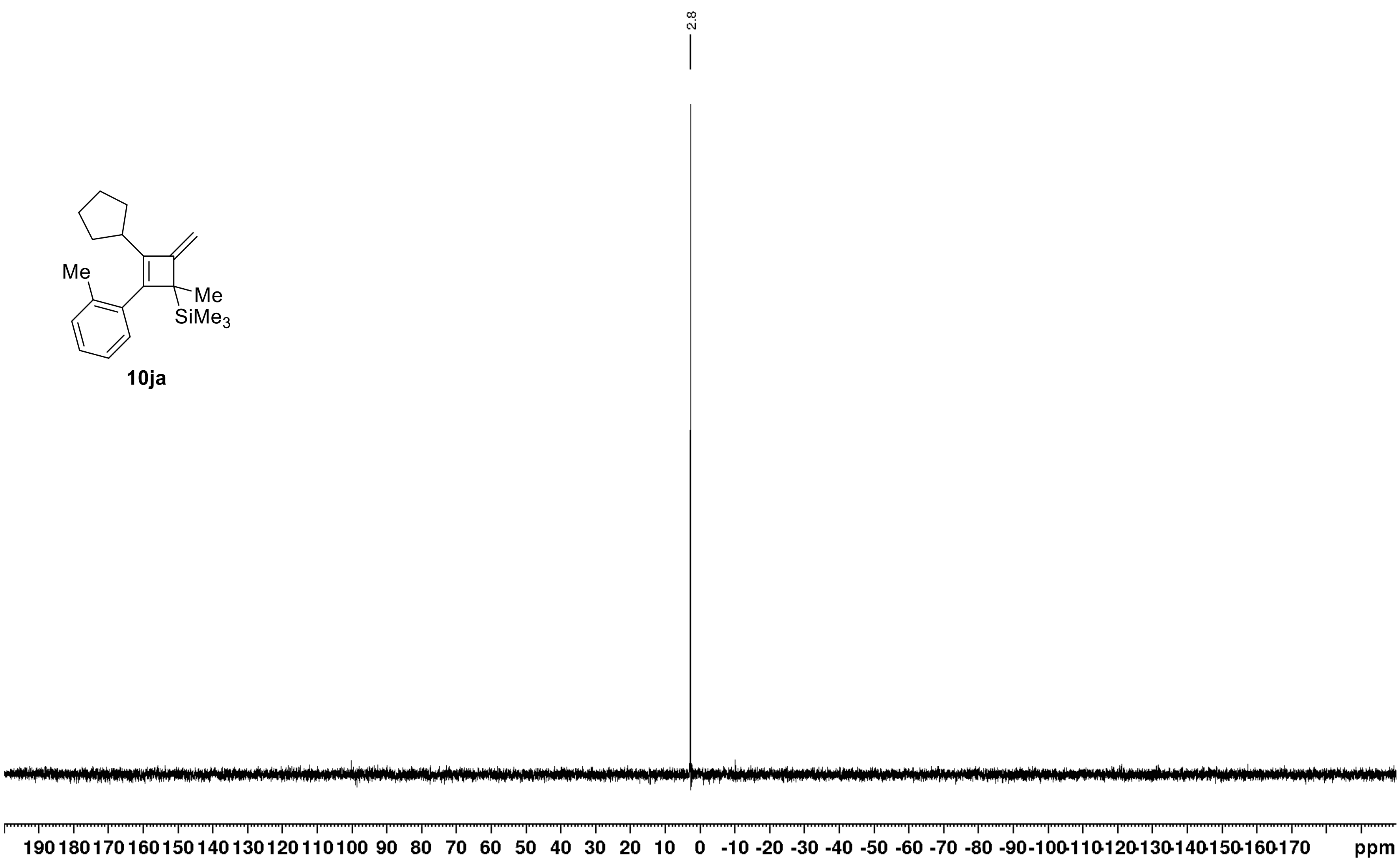

Figure S39. ^{1}H NMR (500 MHz, $CDCl_3$, 298 K) of **10ka**

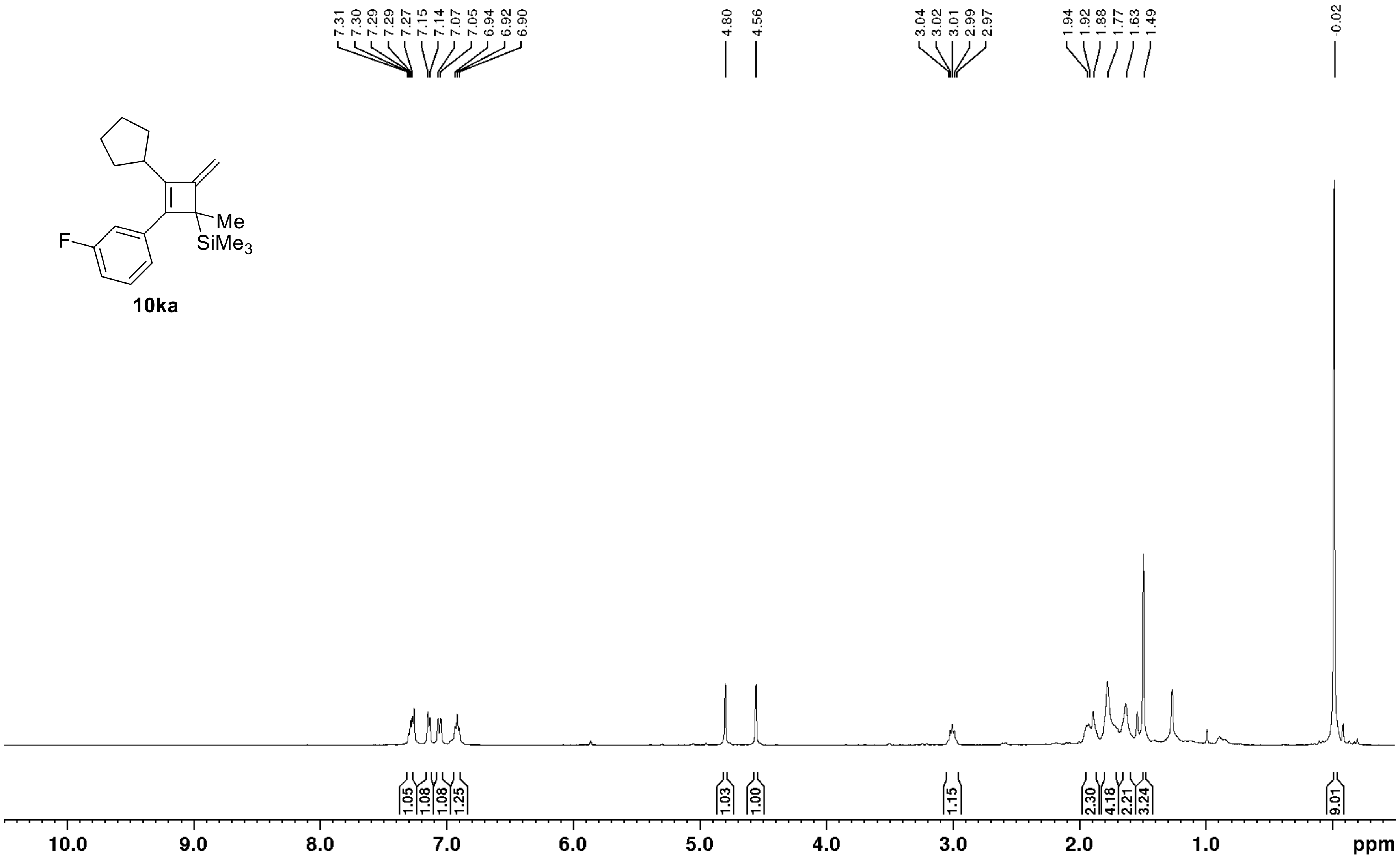

Figure S40. $^{13}C\{^1H\}$ NMR (126 MHz, $CDCl_3$, 298 K) of **10ka**

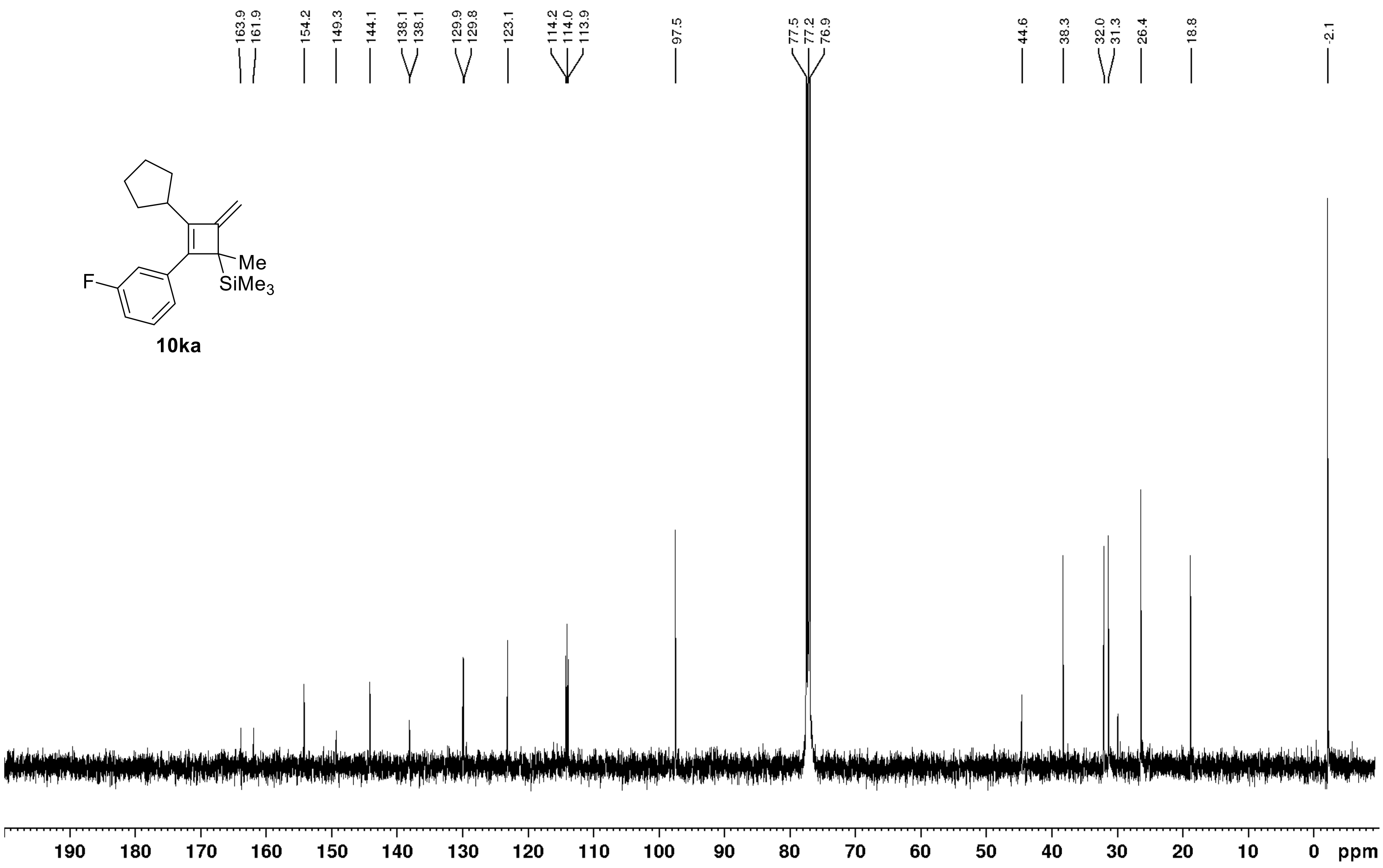

Figure S41. ^{19}F NMR (471 MHz, $CDCl_3$, 298 K) of **10ka**

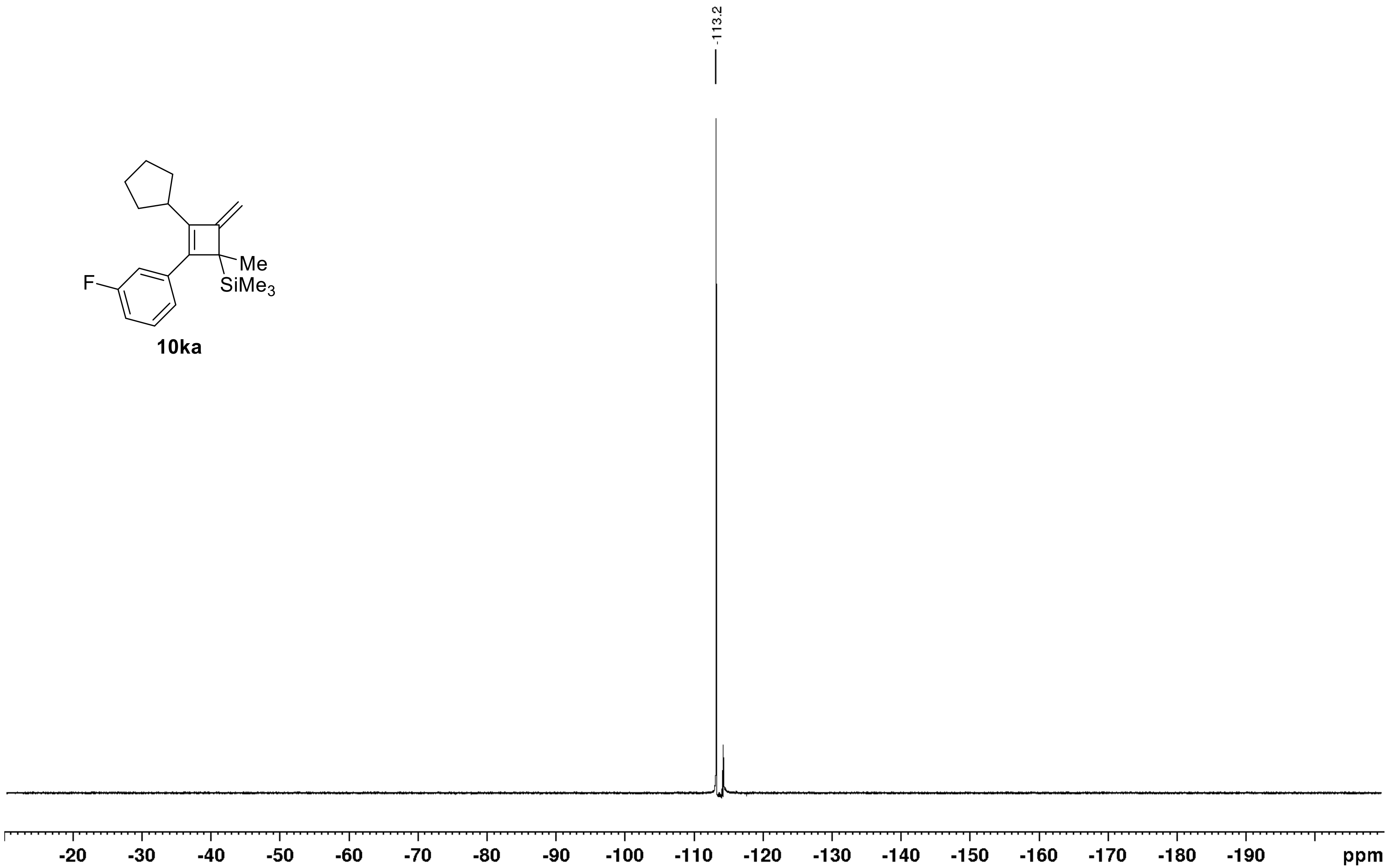

Figure S42. ^{29}Si DEPT NMR (99 MHz, $CDCl_3$, 298 K, optimized for J = 7.0 Hz) of **10ka**

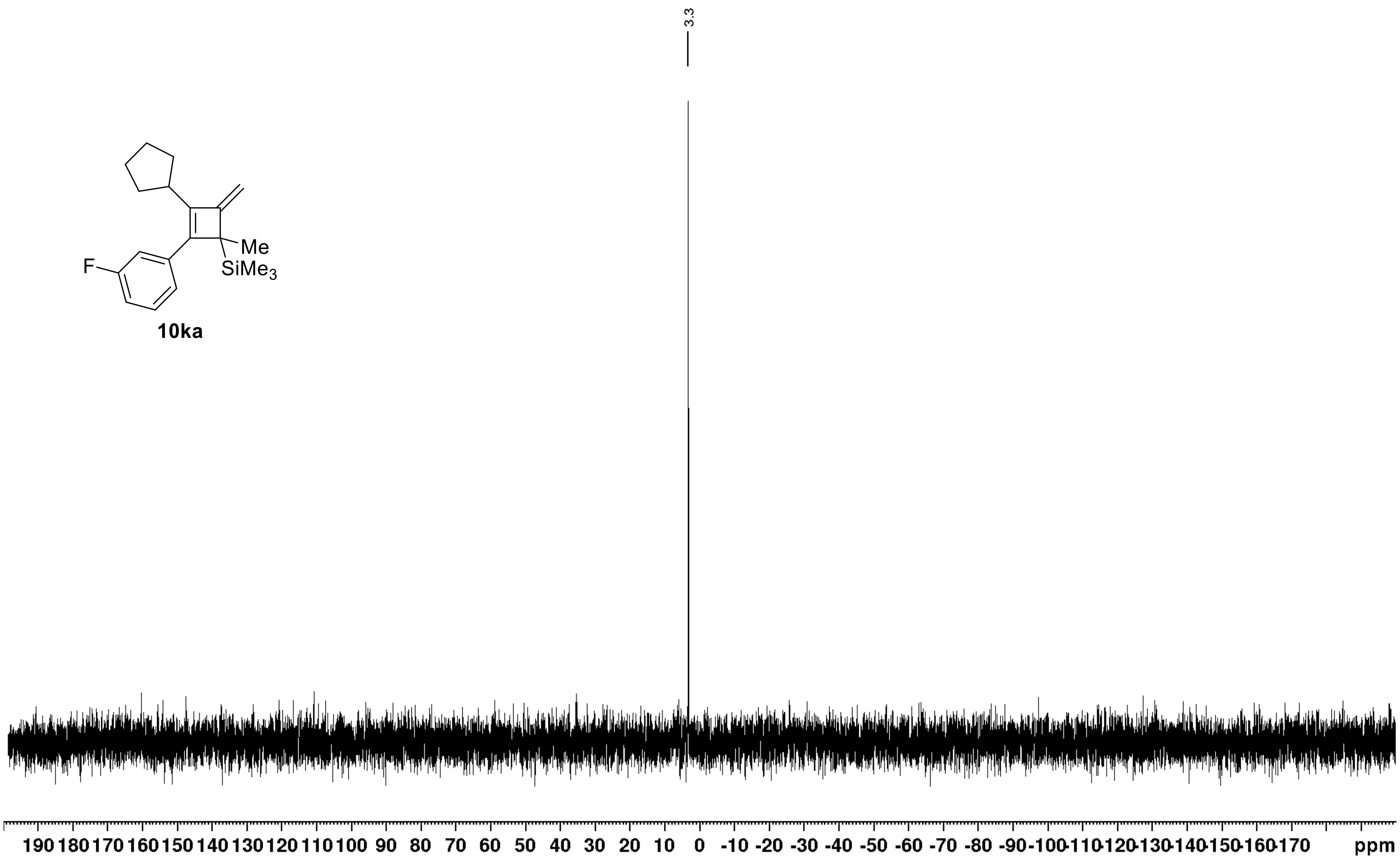

Figure S43. ^{1}H NMR (500 MHz, $CDCl_3$, 298 K) of **10la**

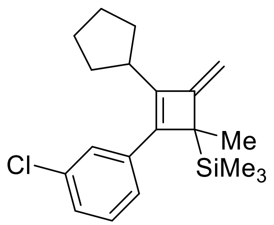

10la

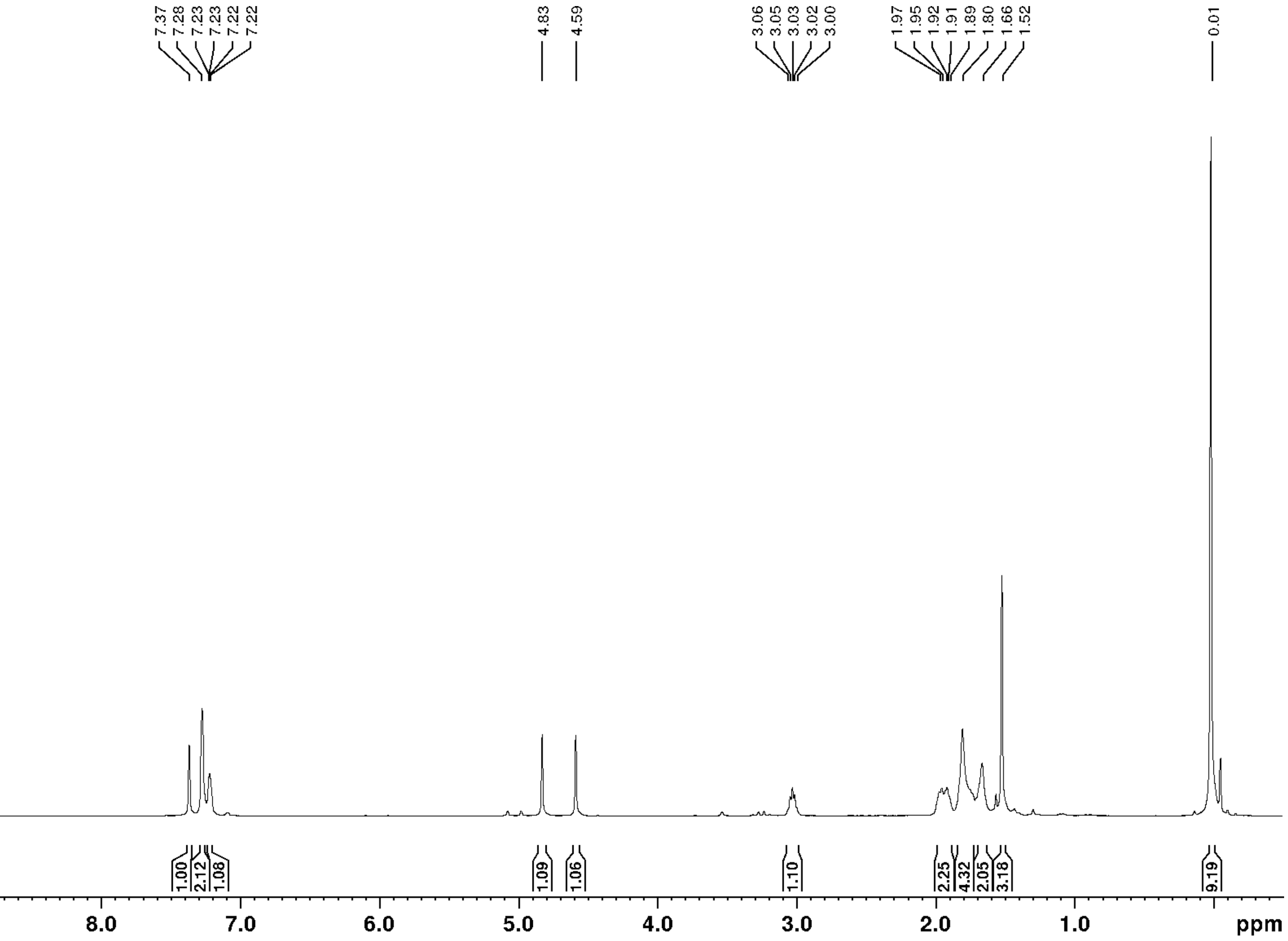

Figure S44. $^{13}C\{^1H\}$ NMR (126 MHz, $CDCl_3$, 298 K) of **10la**

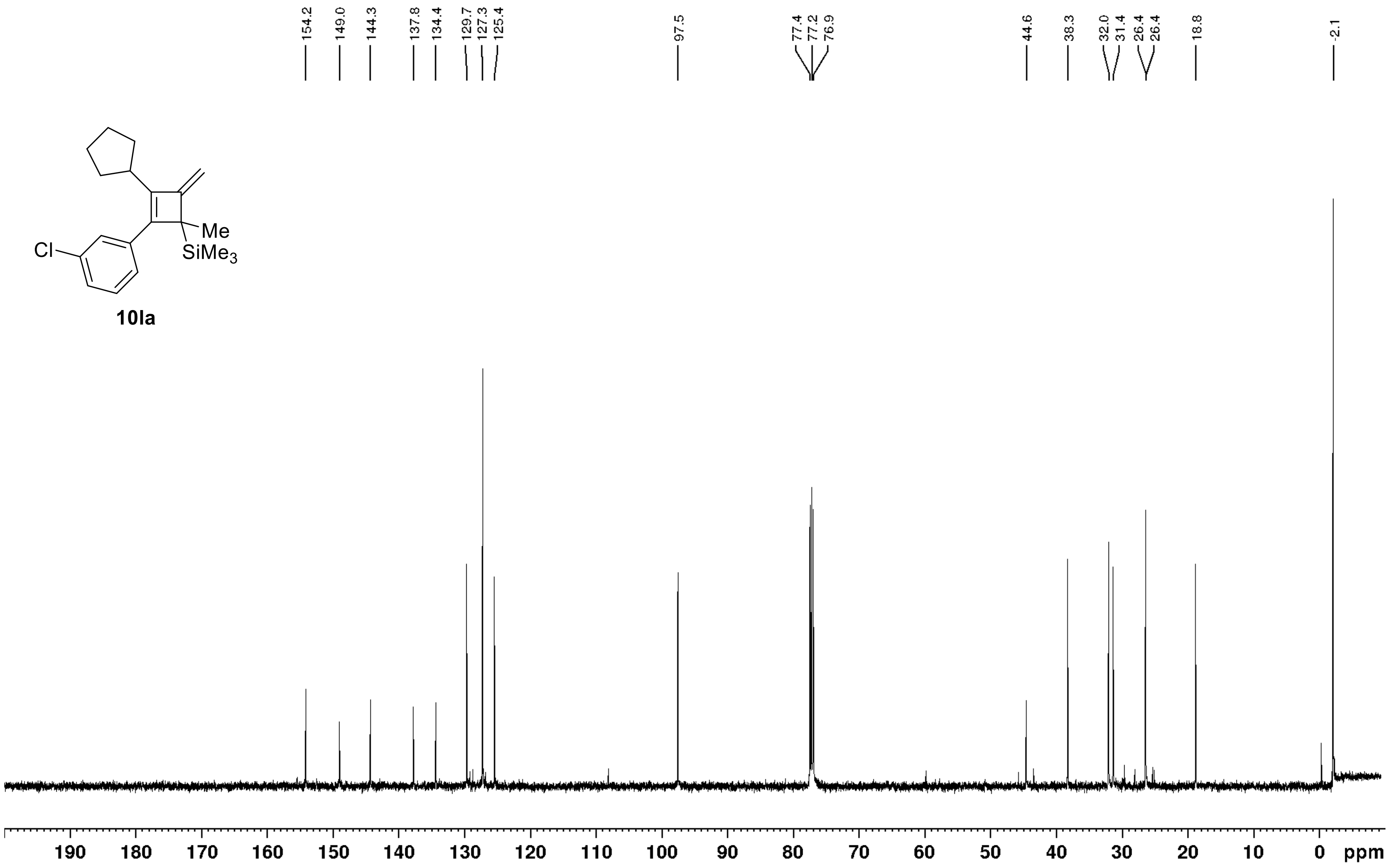

10la

Figure S45. ^{29}Si DEPT NMR (99 MHz, $CDCl_3$, 298 K, optimized for J = 7.0 Hz) of **10la**

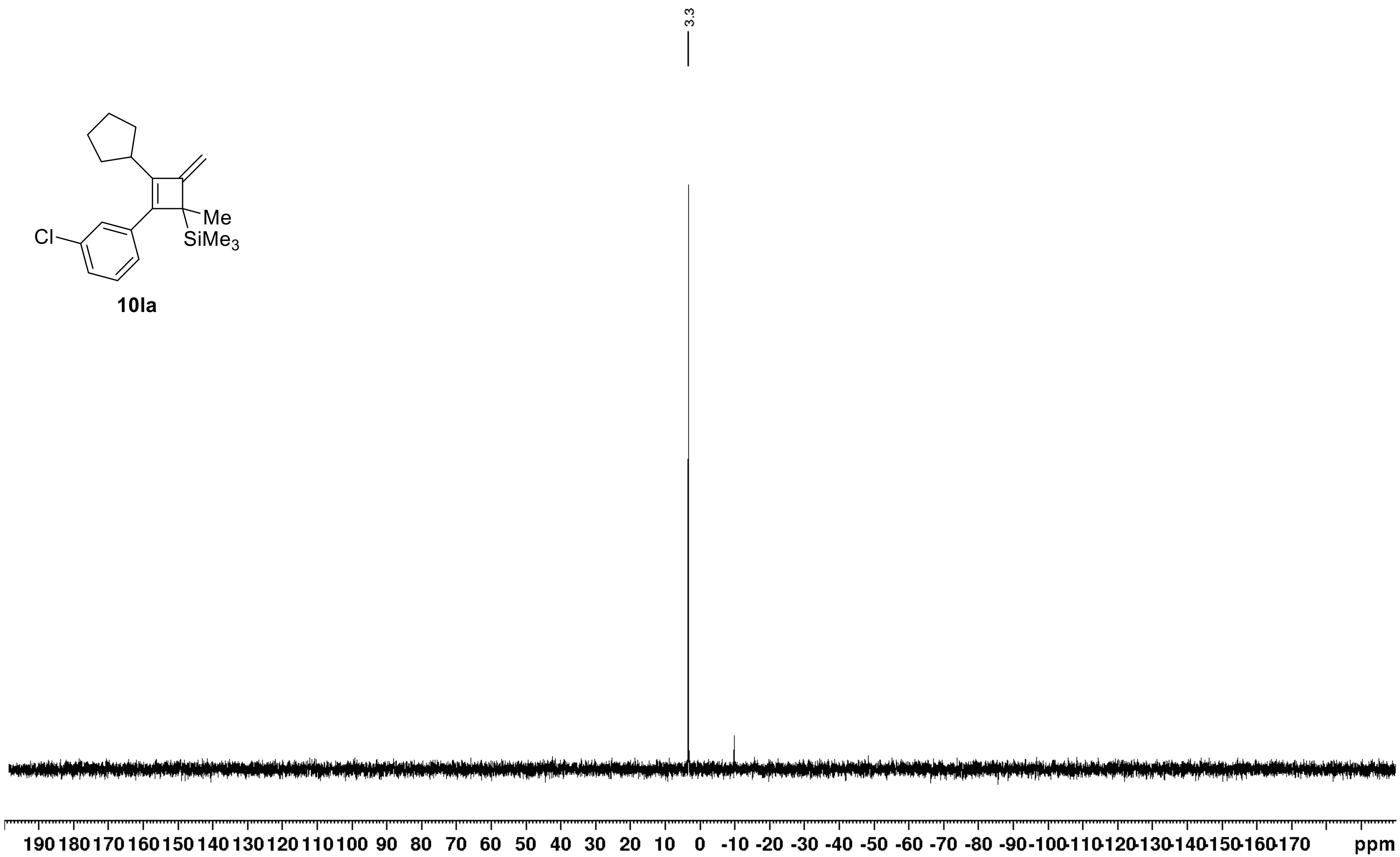

Figure S46. ^{1}H NMR (500 MHz, $CDCl_3$, 298 K) of **10ma**

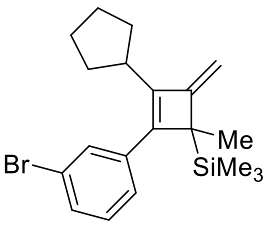

10ma

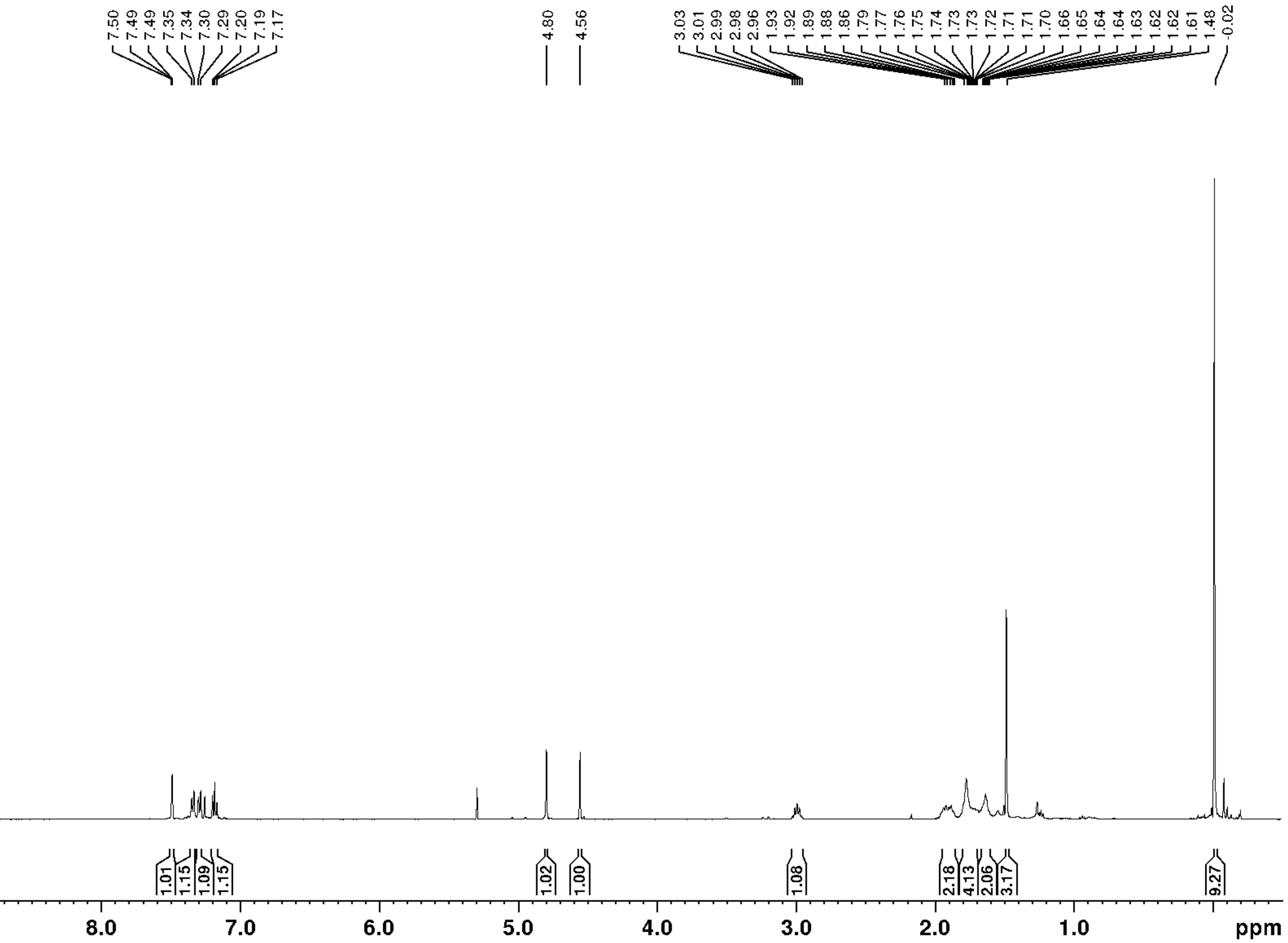

Figure S47. $^{13}C\{^1H\}$ NMR (126 MHz, $CDCl_3$, 298 K) of **10ma**

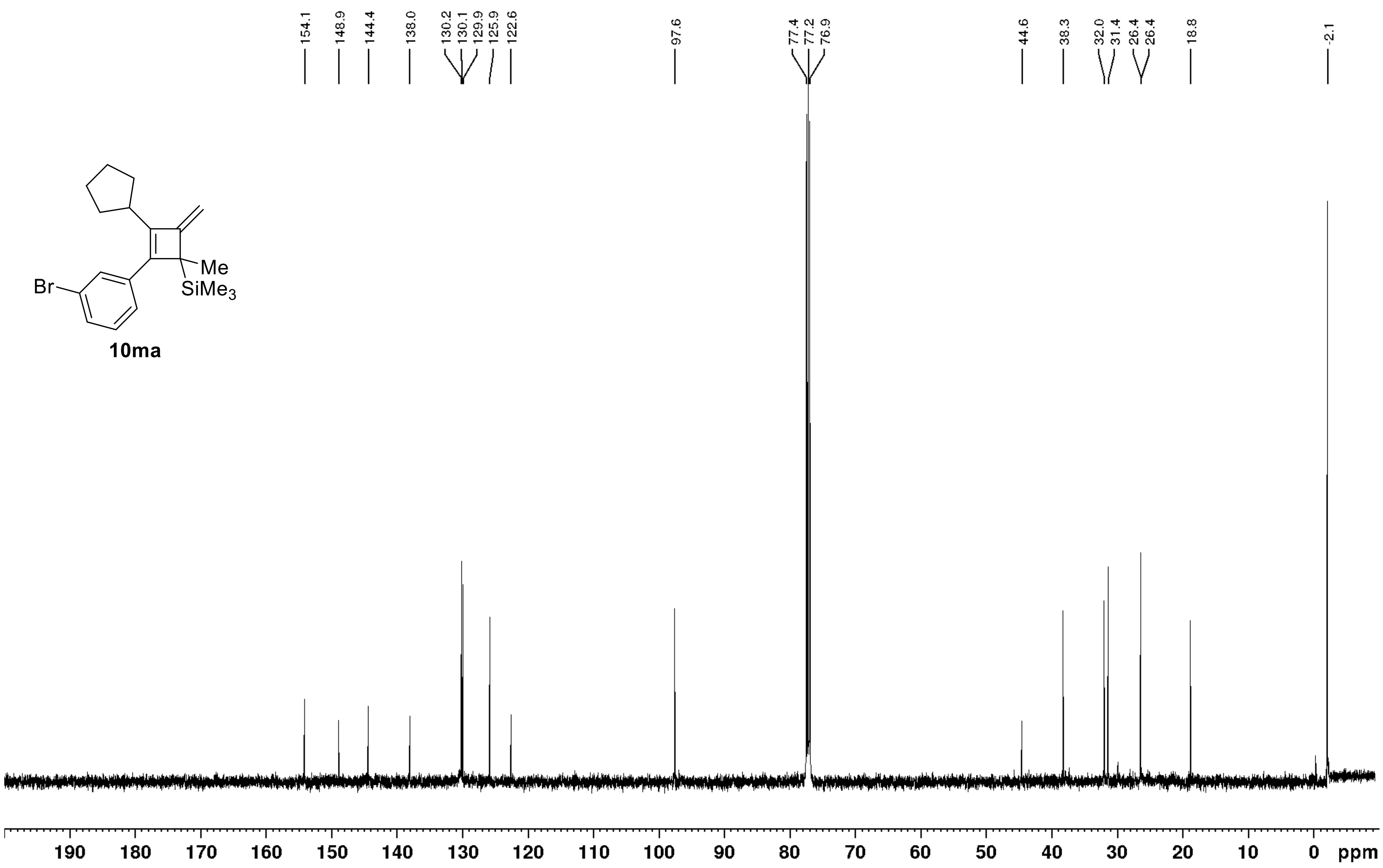

Figure S48. ^{29}Si DEPT NMR (99 MHz, $CDCl_3$, 298 K, optimized for J = 7.0 Hz) of **10ma**

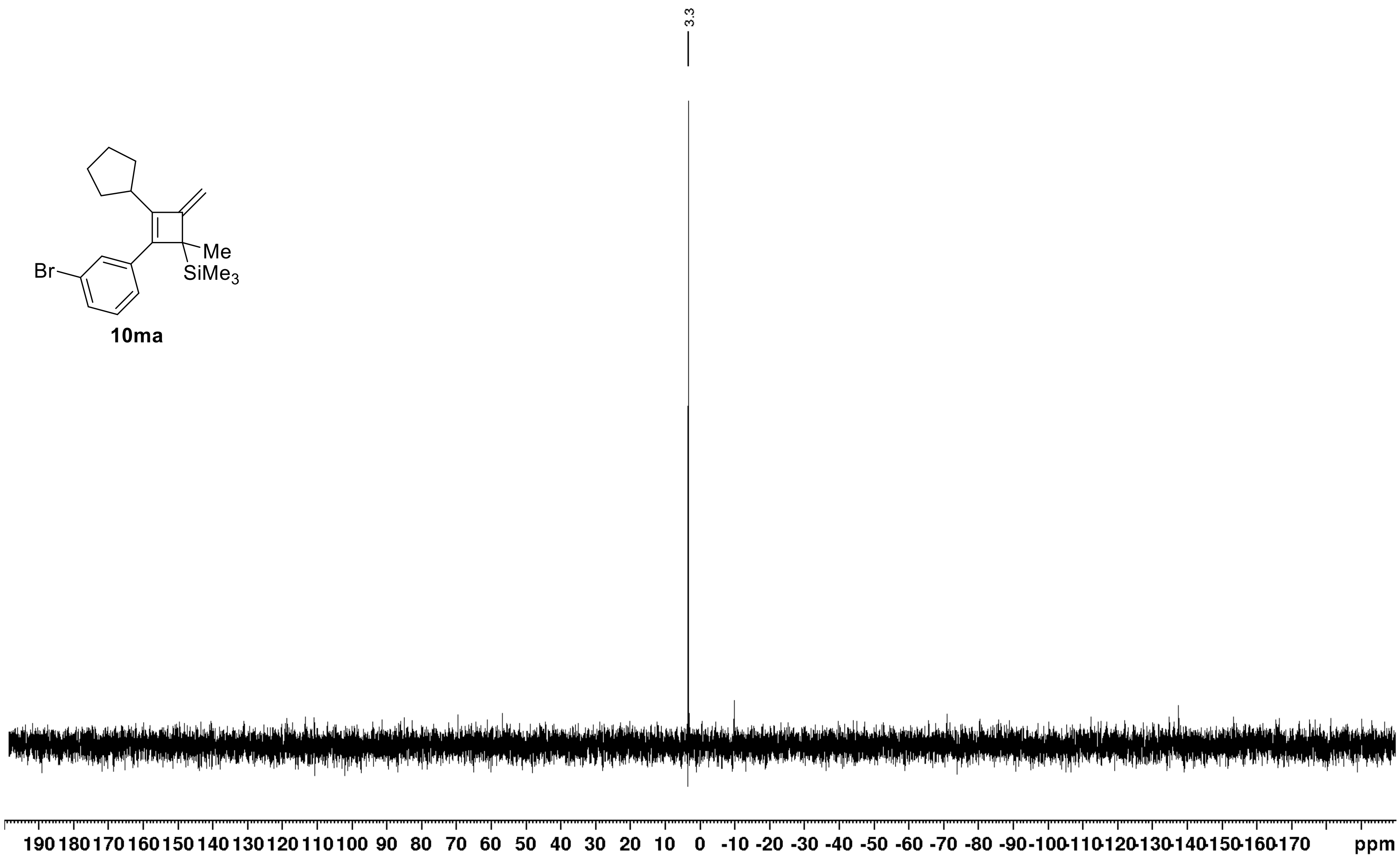

Figure S49. ^{1}H NMR (500 MHz, $CDCl_3$, 298 K) of **10ab**

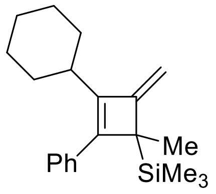

10ab

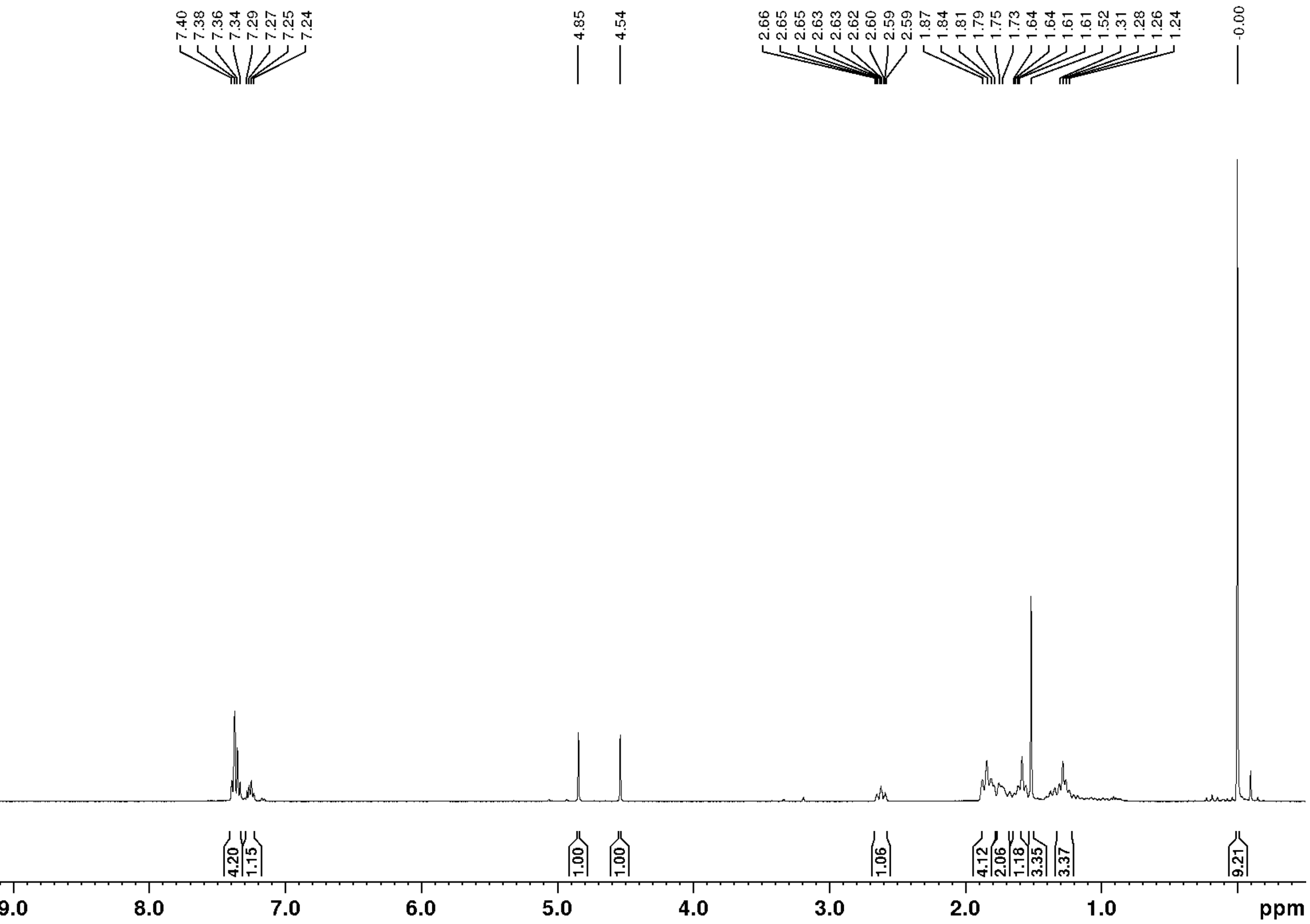

Figure S50. $^{13}C\{^{1}H\}$ NMR (126 MHz, $CDCl_3$, 298 K) of **10ab**

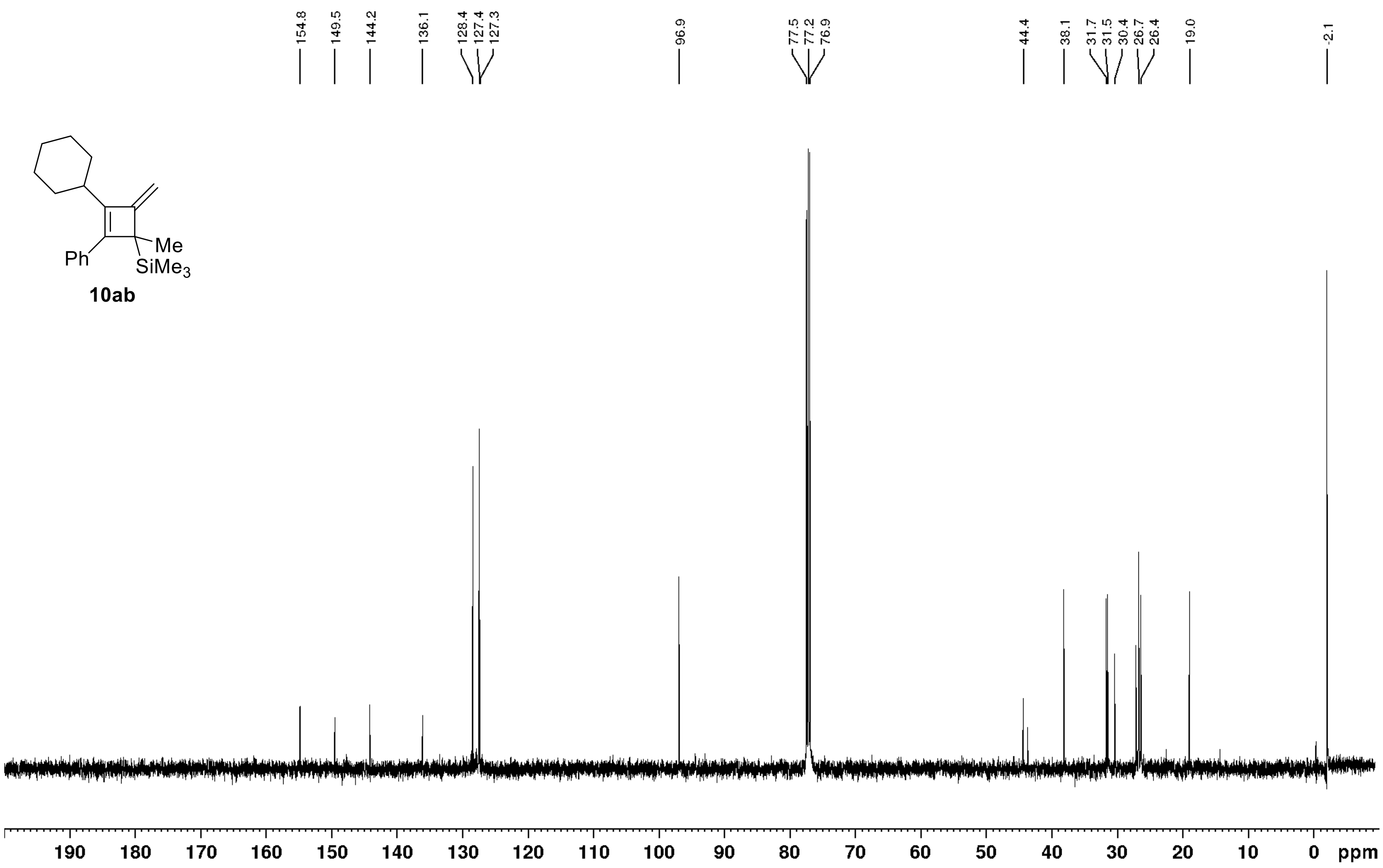

Figure S51. ^{29}Si DEPT NMR (99 MHz, $CDCl_3$, 298 K, optimized for J = 7.0 Hz) of **10ab**

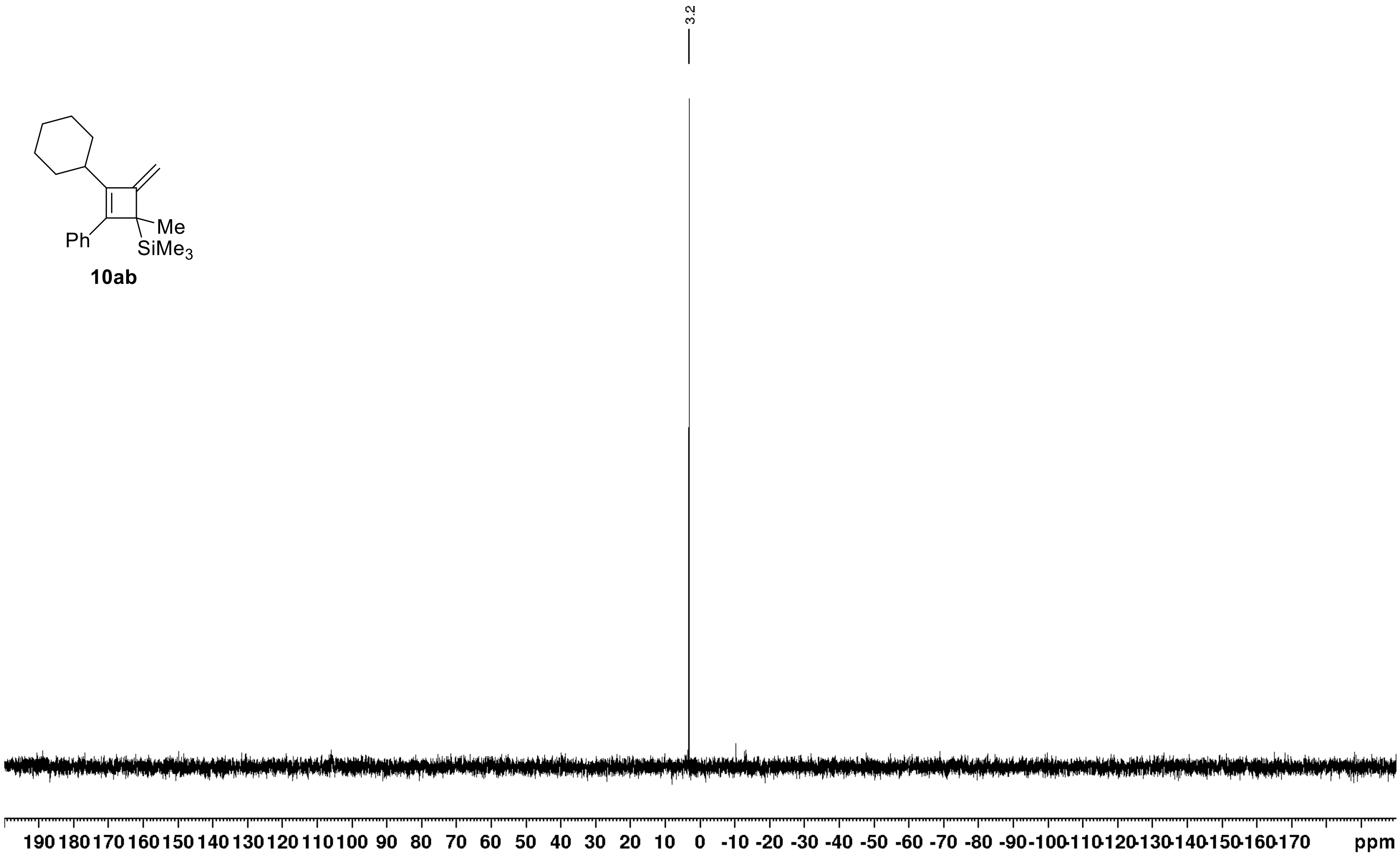

Figure S52. ^{1}H NMR (500 MHz, $CDCl_3$, 298 K) of **10ac**

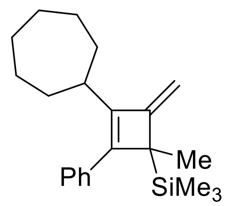

10ac

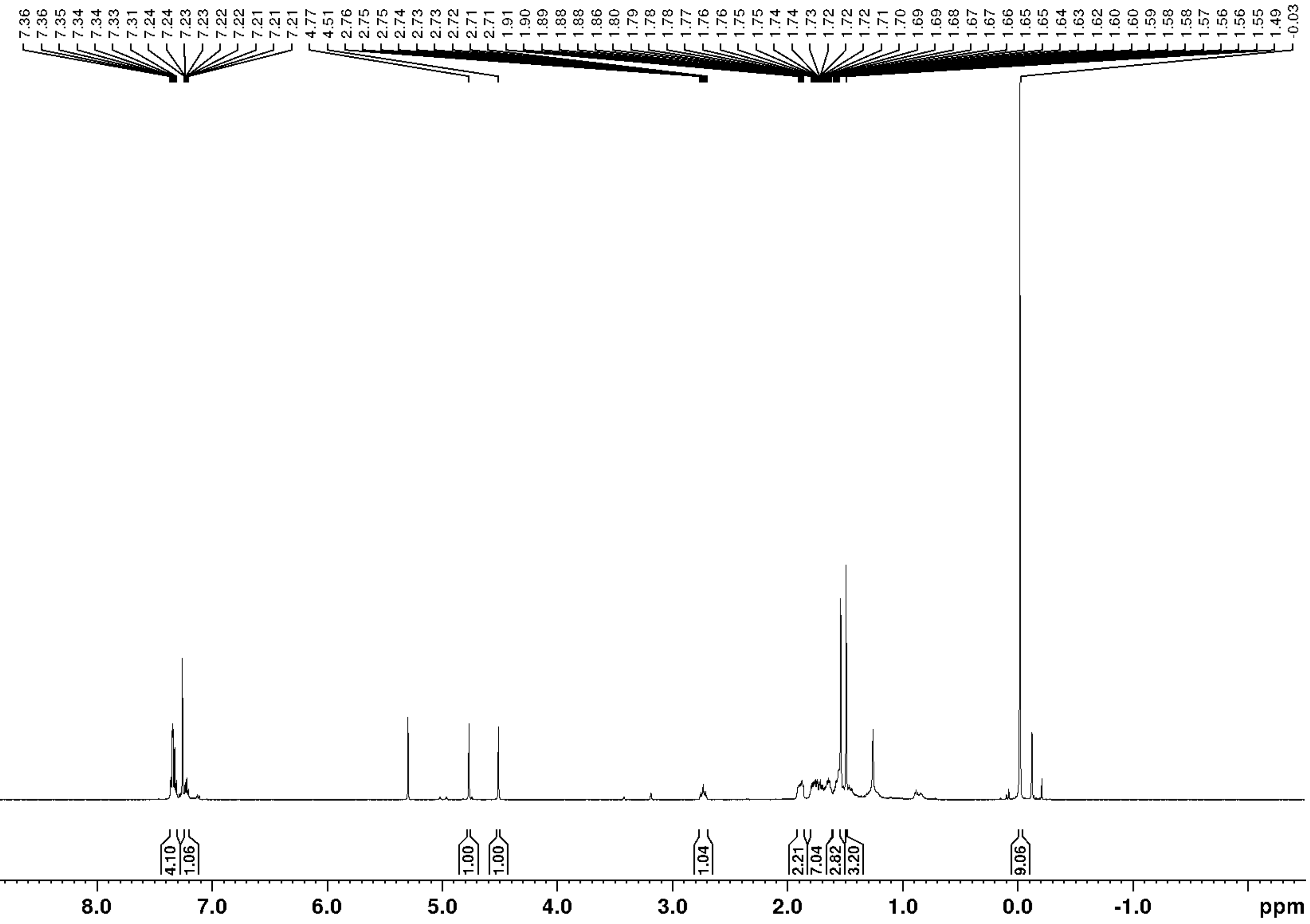

Figure S53. $^{13}C\{^1H\}$ NMR (126 MHz, $CDCl_3$, 298 K) of **10ac**

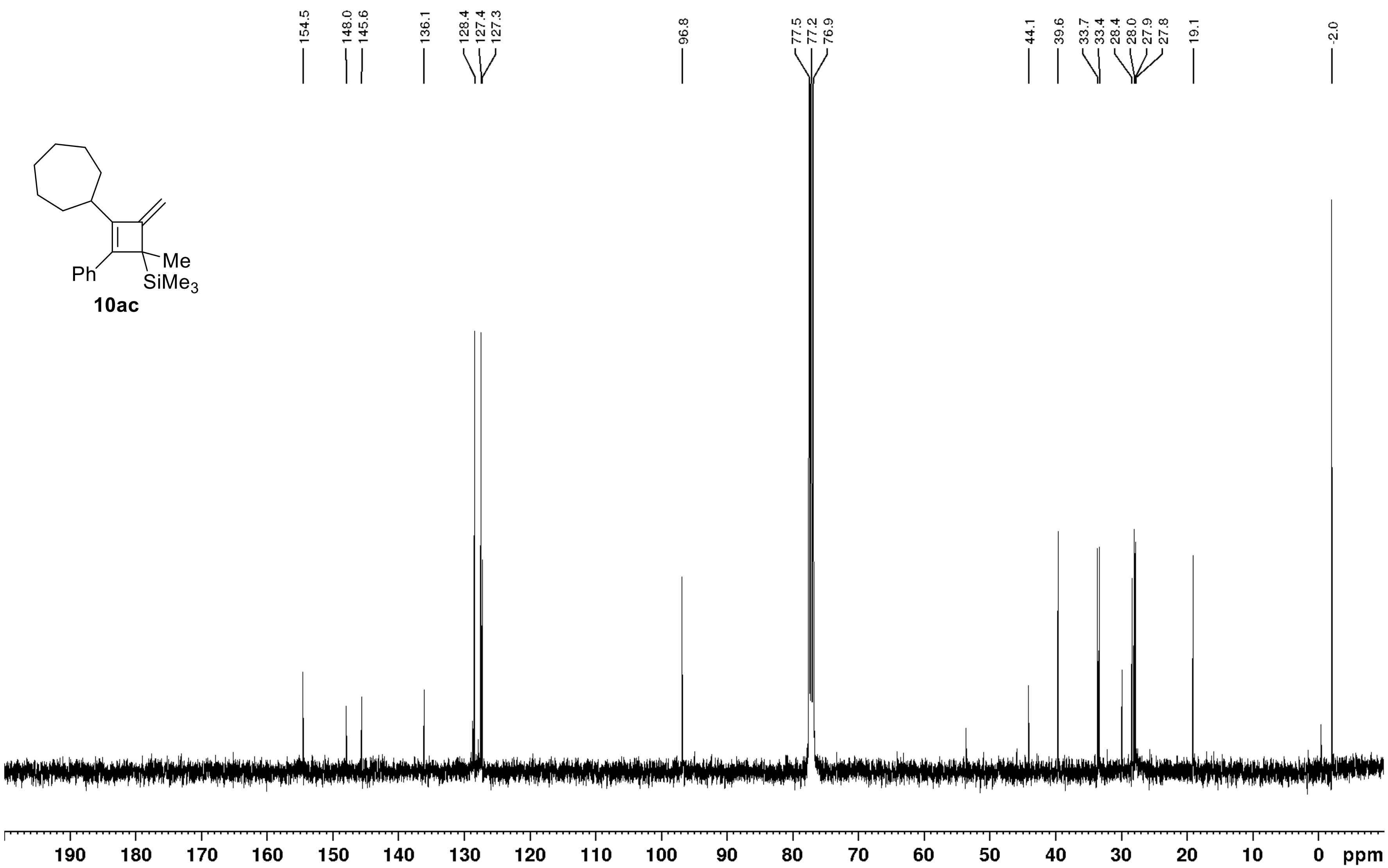

Figure S54. ^{29}Si DEPT NMR (99 MHz, $CDCl_3$, 298 K, optimized for J = 7.0 Hz) of **10ac**

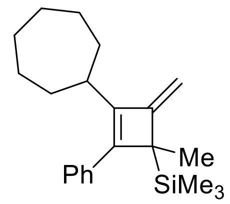

10ac

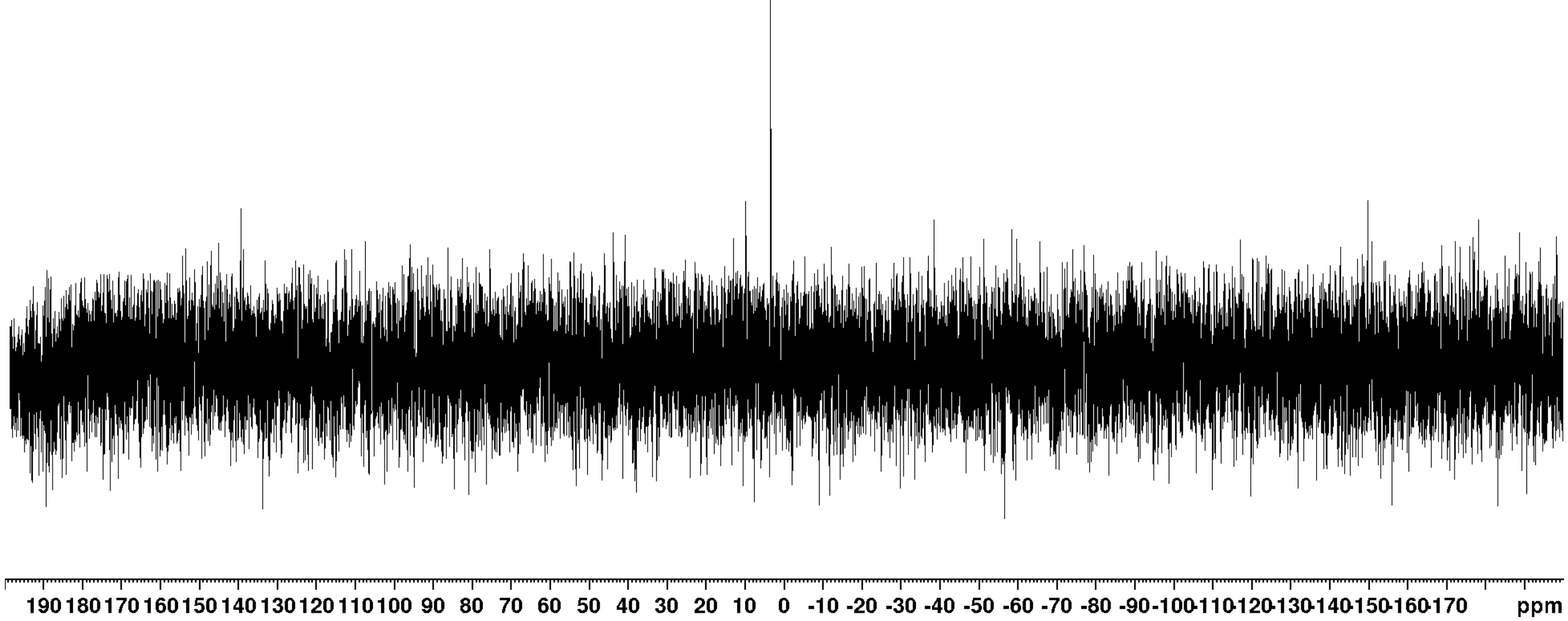

Figure S55. ^{1}H NMR (500 MHz, $CDCl_3$, 298 K) of **10ad**

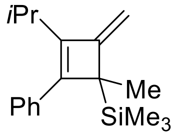

10ad

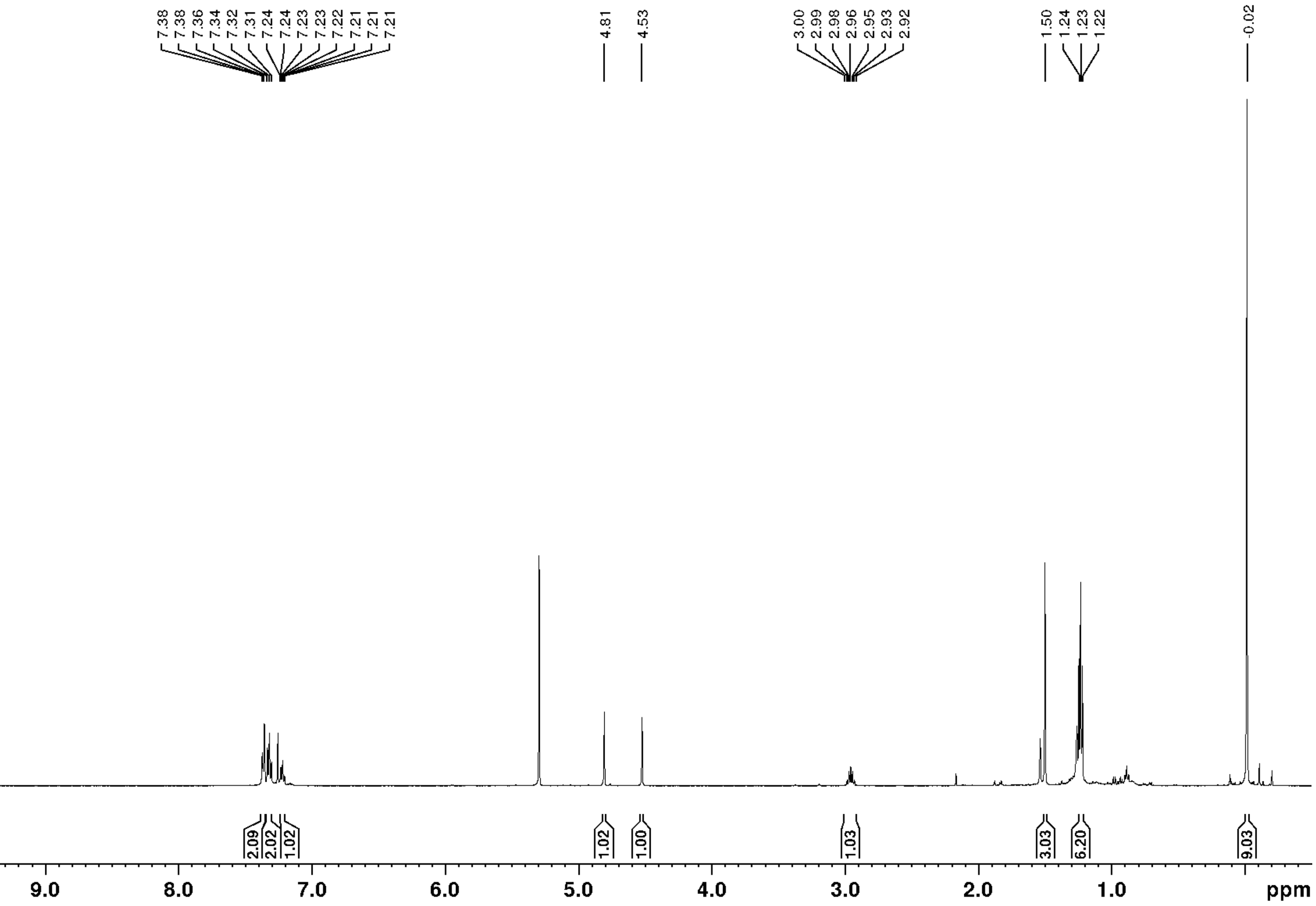

Figure S56. $^{13}C\{^1H\}$ NMR (126 MHz, $CDCl_3$, 298 K) of **10ad**

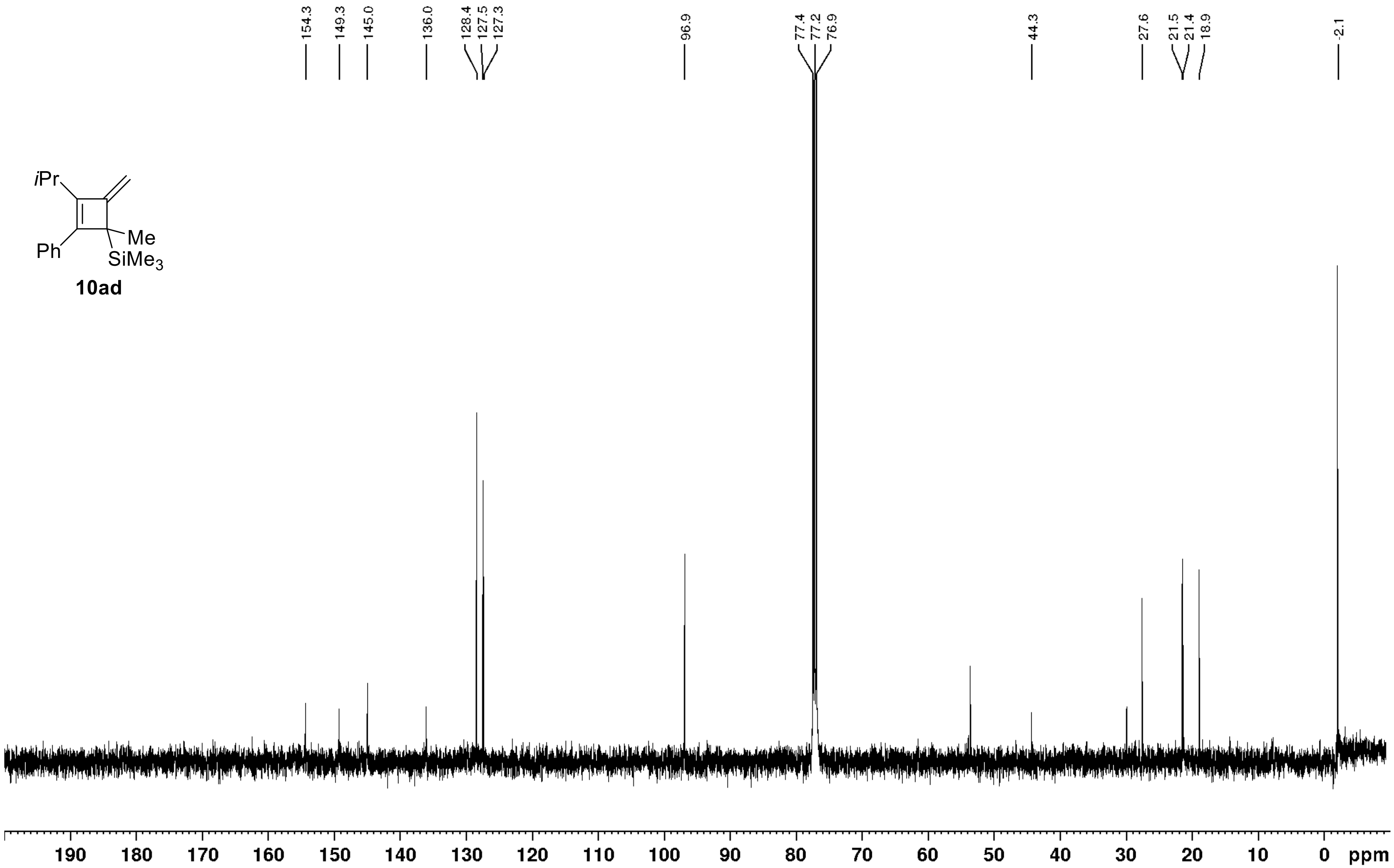

Figure S57. ^{29}Si DEPT NMR (99 MHz, $CDCl_3$, 298 K, optimized for J = 7.0 Hz) of **10ad**

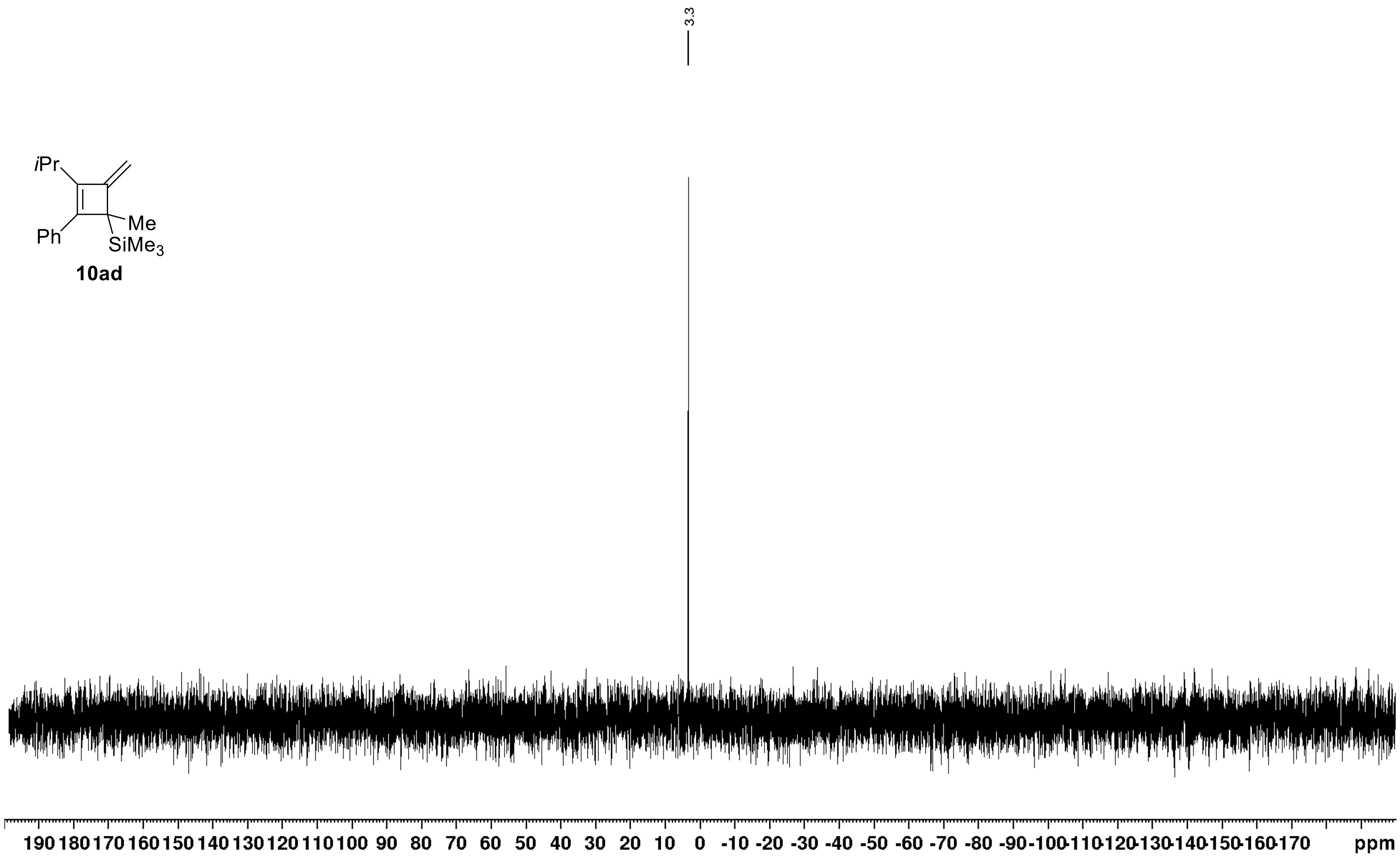

6 References

[S1] Omann, L.; Pudasaini, B.; Irran, E.; Klare, H. F. T.; Baik, M.-H.; Oestreich, M., Thermodynamic Versus Kinetic Control in Substituent Redistribution Reactions of Silylium Ions Steered by the Counteranion. *Chem. Sci.* **2018,** *9*, 5600−5607.

[S2] Harris, R. K.; Becker, E. D.; Cabral de Menezes, S. M.; Goodfellow, R.; Granger, P., NMR Nomenclature. Nuclear Spin Properties and Conventions for Chemical Shifts (IUPAC Recommendations 2001). *Pure Appl. Chem.* **2001,** *73*, 1795−1818.

[S3] Danheiser, R. L.; Carini, D. J.; Fink, D. M.; Basak, A., Scope and Stereochemical Course of the (Trimethylsilyl)Cyclopentene Annulation. *Tetrahedron* **1983,** *39*, 935−947.

[S4] Danheiser, R. L.; Tsai, Y.-M.; Fink, D. M., A General Method for the Synthesis of Allenylsilanes: 1-Methyl-1-(Trimethylsilyl)Allene. *Org. Synth.* **1988,** *66*, 1.

[S5] Westmijze, H.; Vermeer, P., A New and General Route to 1-Trimethylsilyl-1,2-alkadienes. *Synthesis* **1979,** 390−392.

[S6] Zuo, H.; Qu, Z.-W.; Kemper, S.; Klare, H. F. T.; Grimme, S.; Oestreich, M., Silylium-Ion-Promoted (3 + 2) Annulation of Allenylsilanes with Internal Alkynes Involving a Pentadienyl-to-Allyl Cation Electrocyclization. *J. Am. Chem. Soc.* **2024,** *146*, 31377−31383.
